# KCNK1 promotes proliferation and metastasis of breast cancer cells by activating lactate dehydrogenase A (LDHA) and up-regulating H3K18 lactylation

**DOI:** 10.1371/journal.pbio.3002666

**Published:** 2024-06-21

**Authors:** Xiangchan Hou, Jiawei Ouyang, Le Tang, Pan Wu, Xiangying Deng, Qijia Yan, Lei Shi, Songqing Fan, Chunmei Fan, Can Guo, Qianjin Liao, Yong Li, Wei Xiong, Guiyuan Li, Zhaoyang Zeng, Fuyan Wang

**Affiliations:** 1 NHC Key Laboratory of Carcinogenesis and Hunan Key Laboratory of Cancer Metabolism, Hunan Cancer Hospital and Affiliated Cancer Hospital of Xiangya School of Medicine, Central South University, Changsha, Hunan, China; 2 Key Laboratory of Carcinogenesis and Cancer Invasion of the Chinese Ministry of Education, Cancer Research Institute, Central South University, Changsha, Hunan, China; 3 Department of Pathology, Xiangya Hospital, Central South University, Changsha, Hunan, China; 4 Department of Pathology, the Second Xiangya Hospital, Central South University, Changsha, Hunan, China; 5 Department of Medicine, Dan L Duncan Comprehensive Cancer Center, Baylor College of Medicine, Houston, Texas, United States of America; Consejo Nacional de Investigaciones Científicas y Técnicas: Consejo Nacional de Investigaciones Cientificas y Tecnicas, ARGENTINA

## Abstract

Breast cancer is the most prevalent malignancy and the most significant contributor to mortality in female oncology patients. Potassium Two Pore Domain Channel Subfamily K Member 1 (KCNK1) is differentially expressed in a variety of tumors, but the mechanism of its function in breast cancer is unknown. In this study, we found for the first time that KCNK1 was significantly up-regulated in human breast cancer and was correlated with poor prognosis in breast cancer patients. KCNK1 promoted breast cancer proliferation, invasion, and metastasis in vitro and vivo. Further studies unexpectedly revealed that KCNK1 increased the glycolysis and lactate production in breast cancer cells by binding to and activating lactate dehydrogenase A (LDHA), which promoted histones lysine lactylation to induce the expression of a series of downstream genes and LDHA itself. Notably, increased expression of LDHA served as a vicious positive feedback to reduce tumor cell stiffness and adhesion, which eventually resulted in the proliferation, invasion, and metastasis of breast cancer. In conclusion, our results suggest that KCNK1 may serve as a potential breast cancer biomarker, and deeper insight into the cancer-promoting mechanism of KCNK1 may uncover a novel therapeutic target for breast cancer treatment.

## Abstract 2

KCNK1 is a potassium channel differentially expressed in many tumors, but the mechanisms underlying its function in breast cancer remain unclear. This study shows that KCNK1 is overexpressed in breast cancer promoting proliferation, invasion and metastasis by increasing glycolysis and activating Lactate Dehydrogenase A.

## Introduction

Ion channels are a diverse class of transmembrane and ion-selective glycoproteins that regulate ion exchange between cells and the environment [1–3]. Based on the modes of activation, ion channels are generally classified as voltage-gated, ligand-gated, and mechanosensitive ion channels [4–7]. Numerous studies have demonstrated that ion channel proteins play critical roles in excitable cells such as neurons [8], cardiomyocytes [9], and muscle cells [10], and are promising drug targets for the treatment of various diseases [11,12]. In addition, many studies have reported that ion channels are closely related to tumor progression, especially potassium ion channels. They can participate in the occurrence and development of tumors in a variety of ways and are closely associated with the proliferation and migration of tumor cells [13–19]. However, it is still unclear how dysregulation of ion channels in non-excitable cells leads to disease, especially in a way that is unrelated to membrane potential or ion flux. Potassium ion channels are the most abundant type of ion channels in cells. They are typically divided into 4 categories: voltage-gated potassium channels, calcium-activated potassium channels, inward-rectifying potassium channels, and tandem pore domain potassium channels. KCNK1 is a member of the tandem pore domain potassium channel superfamily. The typical structure of this family is that they all have 4 transmembrane segments and 2 pore structure domains. They are typically used as “leak channels” to maintain the negative membrane potential and are used to control the excitability of the heart and nerves [20,21]. Some studies have reported that the K2P family is closely related to the occurrence and development of tumors [22]; KCNK9 is significantly overexpressed in breast cancer, lung cancer, and oral cancer [23,24], and KCNK2 may be a potential therapeutic target for pancreatic ductal adenocarcinoma [25].

Breast cancer is one of the most prevalent cancers worldwide, but how ion channels participate in the malignant progression of breast cancer is rarely reported. We searched for ion channel proteins differentially expressed in breast cancer by analyzing expression profiling microarrays and found that the potassium ion channel protein KCNK1 was most markedly differentially expressed in breast cancer and that higher KCNK1 expression was correlated with poorer patient prognosis. It is of great interest to elucidate the molecular mechanism of KCNK1 in promoting breast cancer proliferation, invasion, and metastasis.

In this study, we demonstrated that KCNK1 facilitated the malignant process of breast cancer through an unexpected non-ion channel function. Surprisingly, we discovered that KCNK1 accelerated cellular glycolysis and lactate production through binding to and activating LDHA, which promoted histones lysine lactylation and induced the expression of a series of downstream targets including LDHA itself, leading to the proliferation, invasion, and metastasis of breast cancer. These findings revealed a novel function of KCNK1 in breast cancer and elucidation of the underlying mechanism will shed light on the understanding of breast cancer tumorigenesis.

## Results

### KCNK1 is highly expressed in breast cancer and associated with poor prognosis

**Fig 1:**
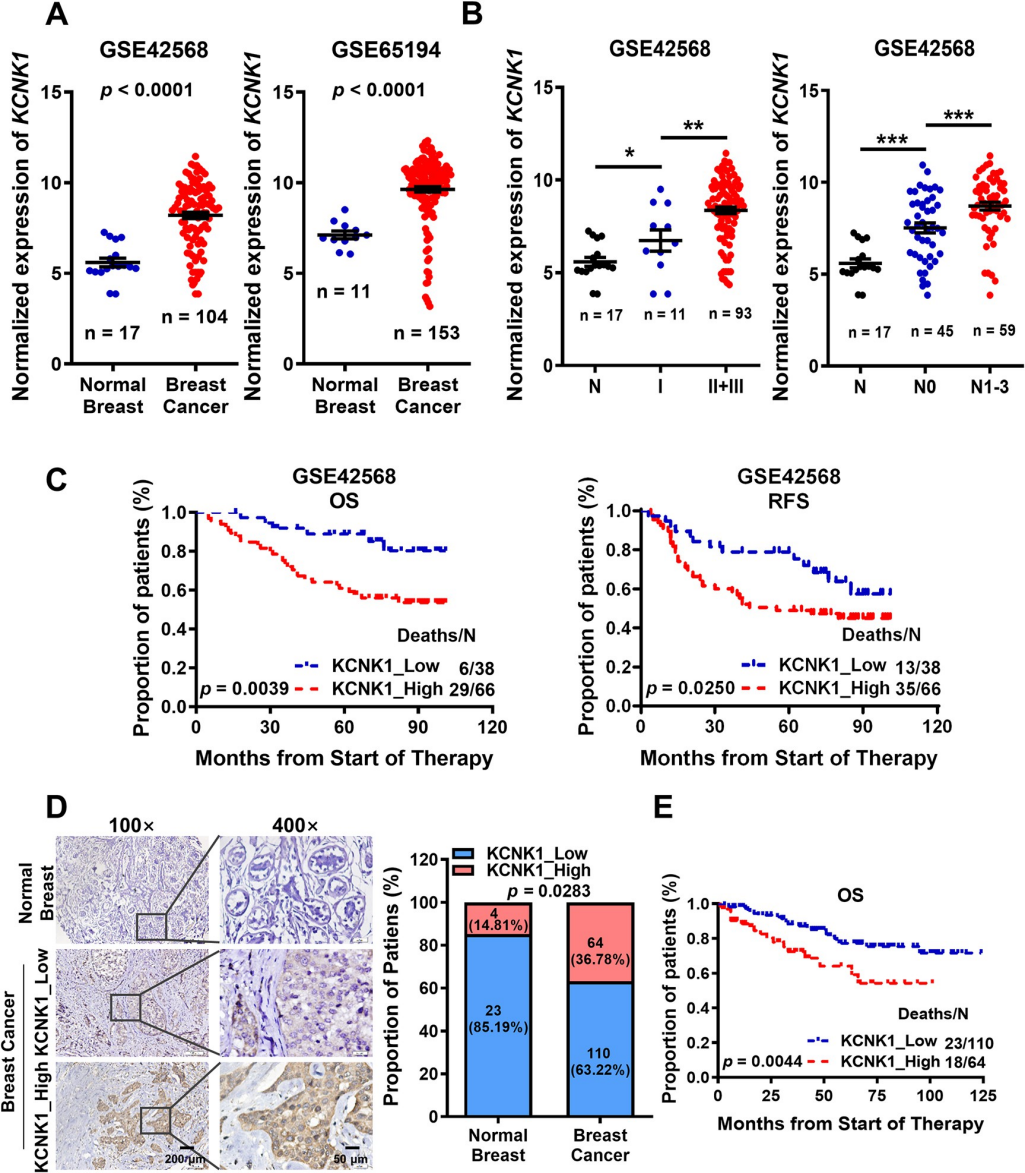
KCNK1 is highly expressed in breast cancer and associated with poor prognosis. **(A)** The expression of KCNK1 in normal breast tissues and breast cancer tissues in GSE42568 and GSE65194. **(B)** Effects of high KCNK1 expression on clinical stages and lymph node metastasis in breast cancer patients in GSE42568 (N0, no regional lymph node involvement; N1, only a few nearby lymph nodes are involved; N2, enlarged ipsilateral axillary lymph nodes fused or adhered to surrounding tissue; N3, distant or more lymph nodes are involved). And *p*-values were calculated by unpaired two-sided *t* test in **A** and **B**. **(C)** Kaplan–Meier analysis showed the effect of high KCNK1 expression on the overall survival and relapse-free survival of breast cancer patients in the GSE42568. **(D)** The expression of KCNK1 was detected by immunohistochemistry in 174 breast cancer tissues and 27 normal breast tissues. Representative field views in matched samples are shown. Data calculated by F-test. **(E)** The effect of high KCNK1 expression (analyzed by immunohistochemistry) on the overall survival of breast cancer patients. Source data are provided as S1 Data.

To screen ion channel proteins differentially expressed in breast cancer, 2 datasets (GSE42568 [26] and GSE65194 [27]) were downloaded from the GEO database (S1 Table), which was based on Affymetrix HG-U133 Plus 2.0 array and contained 193 ion channel genes. Three ion channel genes KCNK1, CLCN3, and CACNB3, which were overexpressed in breast cancer, and another ion channel gene ANO6, which was underexpressed in breast cancer, were identified by significance analysis of microarrays (SAM; Figs 1A, S1A, and S1B). Further analysis showed that KCNK1 was positively associated with clinical stage, lymph node metastasis, poor overall survival, and poor relapse-free survival in breast cancer patients (Fig 1B and 1C). In contrast, the expression of CLCN3, CACNB3, and ANO6 in breast cancer did not correlate significantly with patient prognosis (S1C and S1D Fig). To further confirm the expression of KCNK1 in breast cancer, immunohistochemistry was performed in 27 normal breast tissues and 174 breast cancer tissues (S2 Table). The results showed that the expression of KCNK1 was indeed elevated in clinical breast cancer samples (Fig 1D). Moreover, higher expression of KCNK1 was significantly correlated with poorer overall survival of patients (Fig 1E). These results strongly suggest that KCNK1 may be a novel oncogene and promotes breast cancer tumorigenesis. We used GSE65194 to analyze and found that KCNK1 was significantly overexpressed in all breast cancer subtypes compared to normal tissue (S1E Fig). We also used GSE42568 to analyze and found that there was no significant difference in the expression of KCNK1 between the groups with high and low expression of ER (estrogen receptor), PR (progesterone receptor), and HER-2 (human epidermal growth factor receptor-2) (S1F Fig). These analyses suggest that the expression of KCNK1 is not related to the breast cancer subtypes.

### KCNK1 promotes the proliferation, migration, and invasion of breast cancer cells

**Fig 2:**
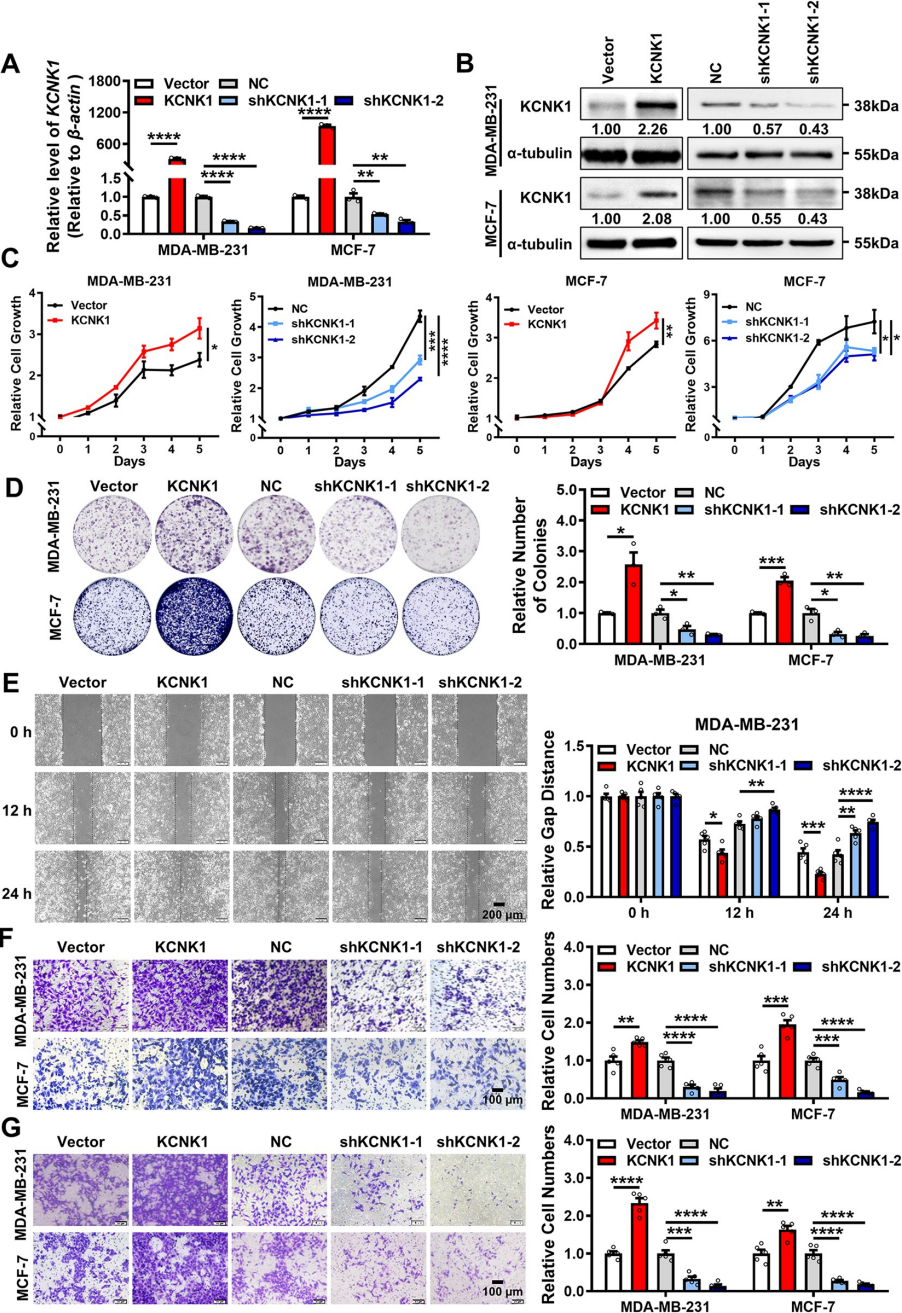
KCNK1 promotes the proliferation, migration, and invasion of breast cancer cells. **(A**, **B)** The overexpression and knockdown efficiencies of KCNK1 were detected by qRT-PCR (**A**) and western blotting assays (**B**) in MDA-MB-231 and MCF-7 cells. **(C**, **D)** The effect of KCNK1 on the proliferation of MDA-MB-231 and MCF-7 was detected by MTT assays (**C**) and clone formation assays (**D**). **(E)** Wound healing experiments were performed after KCNK1 overexpression or shRNAs knockdown in MDA-MB-231 cells. **(F)** The effect of KCNK1 on the migration ability of MDA-MB-231 and MCF-7 was detected in transwell experiments without Matrigel. **(G)** The effect of KCNK1 on the invasion ability of MDA-MB-231 and MCF-7 was detected in transwell experiments with Matrigel. All experiments were triplicated. Data are presented as mean ± SD, analyzed by unpaired two-sided *t* tests. Source data are provided as S1 Data.

To explore the role of KCNK1 in breast cancer, KCNK1 was overexpressed or knocked down in MDA-MB-231, MDA-MB-468, MCF-7, and T47D cells by transfecting the KCNK1 overexpression construct or 2 KCNK1-specific shRNAs, respectively (Figs 2A, 2B, S2A, and S2B). Results of MTT and colony formation assays showed that overexpression of KCNK1 promoted the proliferation of breast cancer cells, whereas KCNK1 knockdown inhibited breast cancer cell proliferation (Figs 2C, 2D, S2C, and S2D). Wound healing and transwell migration experiments demonstrated that the migration ability of breast cancer cells was enhanced after overexpression of KCNK1 and decreased after KCNK1 knockdown (Figs 2E, 2F, S2E, and S2F). Transwell invasion assays further confirmed that overexpression of KCNK1 promoted the invasion of breast cancer cells, while knockdown of KCNK1 inhibited its invasion (Figs 2G and S2G). Collectively, the above results clearly indicate that KCNK1 promotes the proliferation, migration, and invasion of breast cancer cells in vitro.

### KCNK1 promotes metabolic reprogramming in breast cancer cells via binding to and activating LDHA

**Fig 3:**
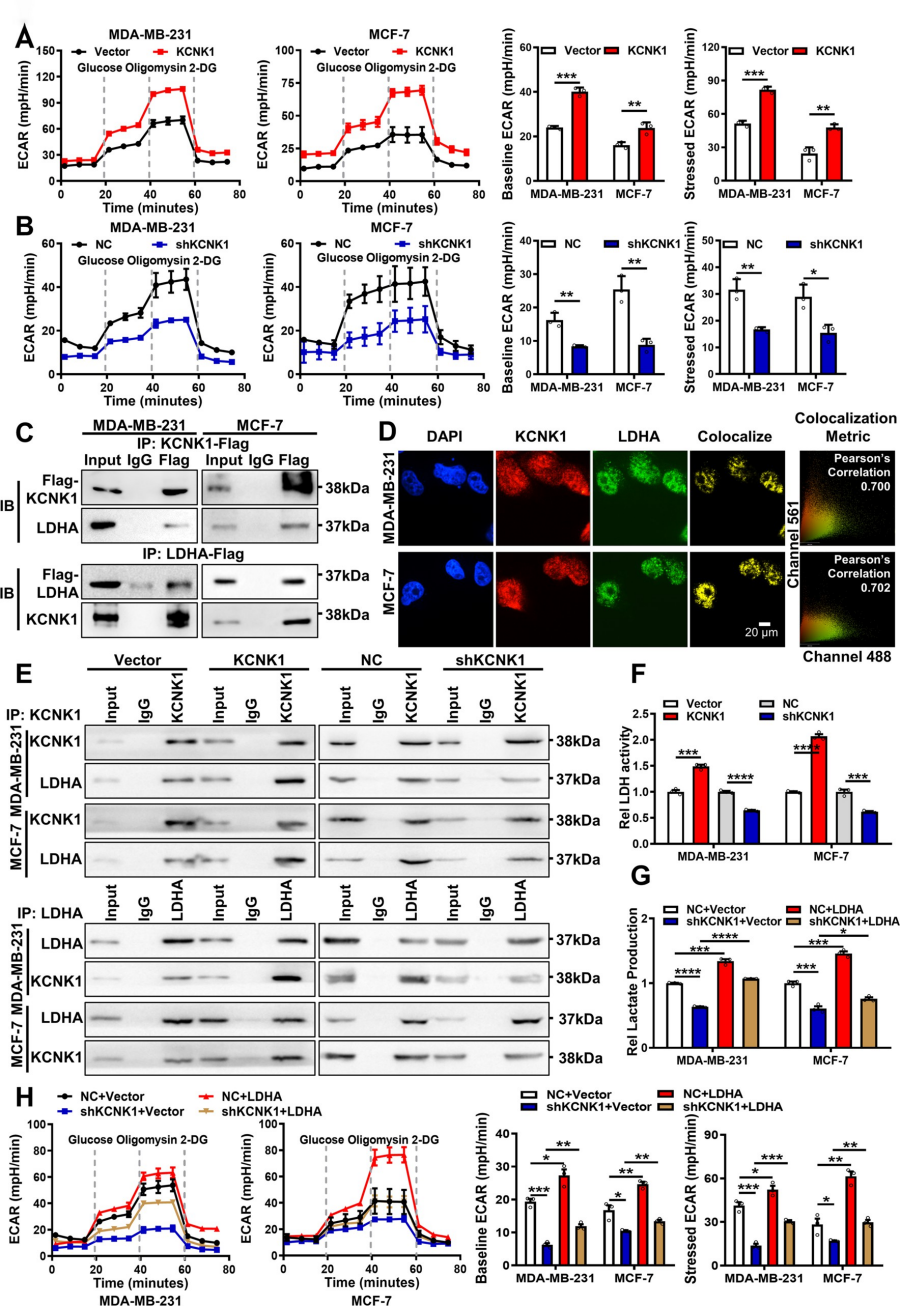
KCNK1 promotes metabolic reprogramming in breast cancer cells via binding to and activating LDHA. **(A)** The ECAR was measured by the Seahorse XF assays in MDA-MB-231 and MCF-7 cells after KCNK1 overexpression (left); baseline ECAR and stressed ECAR were calculated based to the total ECAR (right). **(B)** The ECAR was measured by the Seahorse XF assays in MDA-MB-231 and MCF-7 cells after KCNK1 knockdown (left); baseline ECAR and stressed ECAR were calculated based to the total ECAR (right). **(C)** The interaction between KCNK1 and LDHA was examined by immunoprecipitation using an anti-Flag antibody in MDA-MB-231 and MCF-7 cells transfected with Flag-KCNK1 or Flag-LDHA vector, followed by western blotting using the anti-KCNK1 or anti-LDHA antibodies. **(D)** Immunofluorescence experiments showed that KCNK1 and LDHA were co-localized in MDA-MB-231 and MCF-7 cells. DAPI-stained nucleus: blue; anti- KCNK1: red; anti-LDHA: green; the colocalize image represents the overlap of KCNK1 and LDHA (scale bar: 20 μm). **(E)** Quantitative Co-IP experiments showed that KCNK1 overexpression enhanced their binding capacity while KCNK1 knockdown reduced the capacity. **(F)** The lactate dehydrogenase activity was detected in breast cancer cells after overexpression or knockdown of KCNK1. **(G)** The lactate production ability of breast cancer cells was detected after KCNK1 knockdown, LDHA overexpression, and co-transfection of shKCNK1 and LDHA overexpression vectors. **(H)** The ECAR was measured by the Seahorse XF assay in MDA-MB-231 and MCF-7 cells after KCNK1 knockdown, LDHA overexpression, or co-transfection of shKCNK1 and LDHA overexpression vectors (left); baseline ECAR and stress ECAR were calculated from total ECAR (right). All experiments were triplicated. Data are presented as mean ± SD, analyzed by unpaired two-sided *t* tests. Source data are provided as S1 Data. ECAR, extracellular acidification rate; LDHA, lactate dehydrogenase A.

During the experiment, we noticed that when KCNK1 was overexpressed in MDA-MB-231 and MCF-7 cells, the cell culture medium turned slightly yellow, indicating an acidic extracellular environment, whereas knockdown of KCNK1 with shRNA resulted in a slightly red medium when compared with the control, suggesting a basic extracellular environment (S3A Fig). We speculated whether KCNK1 caused pH change in the extracellular environment through modulating hydrogen-potassium ion exchange. Indeed, levels of lactate were also changed accordingly after overexpression or knockdown of KCNK1, suggesting that KCNK1 modulated the extracellular microenvironment through regulating the production of lactate (S3B Fig). However, Quinine, a KCNK1 inhibitor, did not affect the KCNK1-induced color change of the medium (S3A Fig), or the proliferation (S3C and S3D Fig), migration (S3E and S3F Fig), and invasion (S3G Fig) of breast cancer cells. Meanwhile, we also conducted clamp experiments to detect the effects of overexpression or knockdown of KCNK1 on the resting potential of breast cancer cells. According to the following experimental results, we found that overexpression or knockdown of KCNK1 did not affect the resting potentials of MDA-MB-231 and MCF-7 cells, and the resting potential of these cells did not change significantly after quinine treatment (S3H and S3I Fig), which indicated that the effect of KCNK1 on the malignant phenotype of breast cancer cells is independent of its voltage effect. The results suggested that KCNK1 might be involved in the malignant process of breast cancer through non-ion channel function. Lactate, a product of cellular glycolysis, is the major contributor to the acidic microenvironment in cancer cells [28–31]. To test whether KCNK1 affected glycolysis in breast cancer cells, glycolysis rate (extracellular acidification rate, ECAR) was measured by Seahorse XF Analyzer. The results showed that overexpression of KCNK1 significantly increased the glycolysis of MDA-MB-231 and MCF-7 cells, including the basal and the maximal glycolysis, and the opposite results were obtained after KCNK1 knockdown (Fig 3A and 3B).

To identify potential downstream targets of KCNK1 in breast cancer, liquid chromatography-tandem mass spectrometry (LC-MS/MS) was performed after immunoprecipitation of KCNK1 in MDA-MB-231 cells (S3 Table). Lactate dehydrogenase A (*LDHA*) emerged as the top candidate due to its high LC-MS/MS score and pivotal relevance to the glycolysis process. Co-IP experiments confirmed the direct interaction between KCNK1 and LDHA (Fig 3C). Immunofluorescence also revealed the co-localization of KCNK1 and LDHA in cells (Fig 3D). Moreover, we further confirmed through Co-IP experiments that the binding of KCNK1 and LDHA increased after overexpressing KCNK1, while the binding of them decreased after knocking down KCNK1 (Fig 3E). Importantly, overexpression of KCNK1 enhanced the enzymatic activity of LDHA, while knockdown of KCNK1 inhibited it (Fig 3F). Furthermore, overexpression of LDHA rescued lactate production which was inhibited by knockdown of KCNK1 in MDA-MB-231 and MCF-7 cells (Fig 3G). The results of seahorse assay also confirmed that overexpression of LDHA reversed the inhibitory effect of KCNK1 knockdown on glycolysis including basal and maximal glycolysis in MDA-MB-231 and MCF-7 cells (Fig 3H).

**Fig 4:**
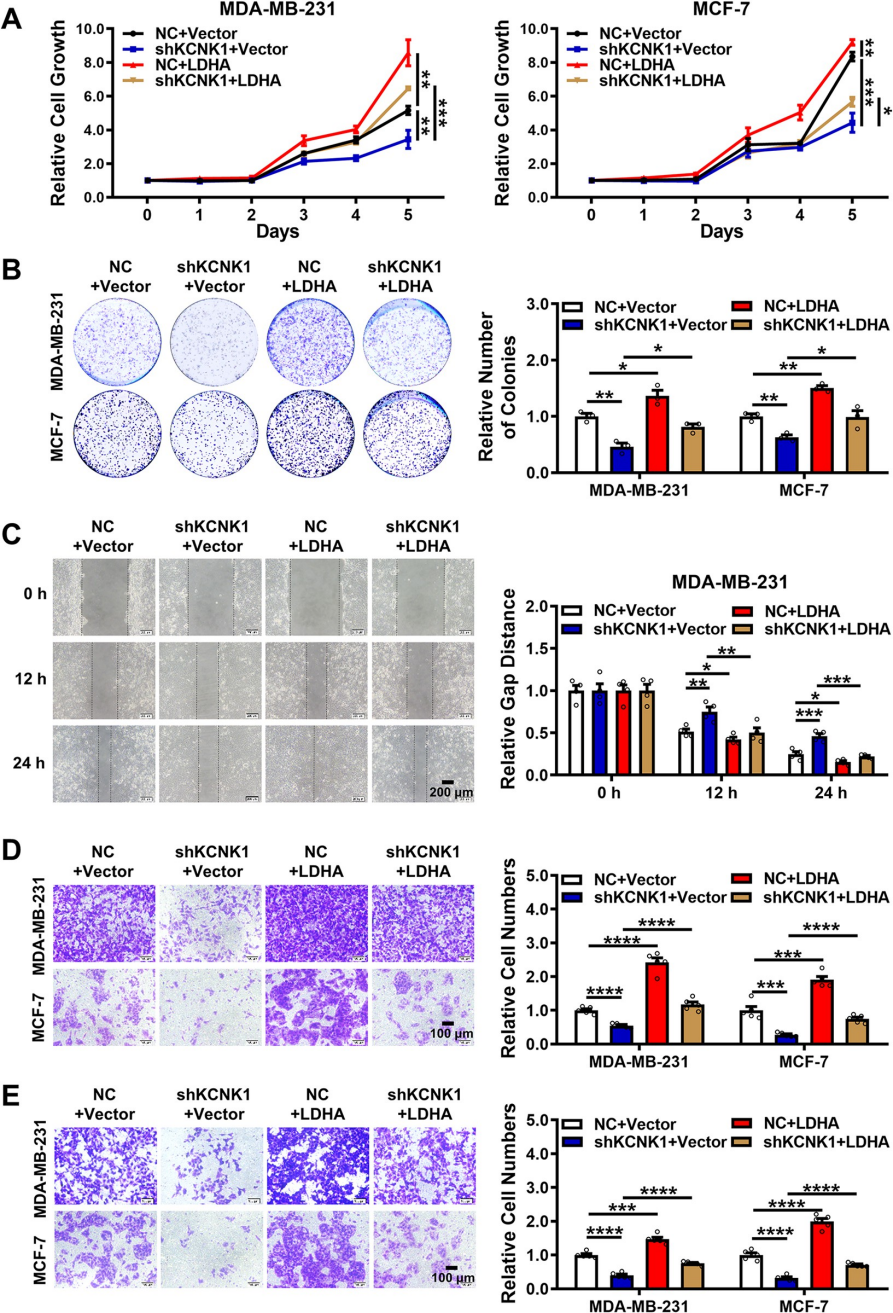
KCNK1 promotes the proliferation, migration, and invasion of breast cancer cells via LDHA. **(A**, **B)** The proliferation ability of MDA-MB-231 and MCF-7 cells were detected by MTT assays (**A**) and clone formation assays (**B**) after KCNK1 knockdown, LDHA overexpression, or co-transfection of shKCNK1 and LDHA overexpression vectors. **(C)** Wound healing experiments were performed to detect the migration ability of MDA-MB-231 cells after KCNK1 knockdown, LDHA overexpression, or co-transfection of shKCNK1 and LDHA overexpression vectors. **(D)** Transwell experiments without Matrigel were performed to detect the migration ability of MDA-MB-231 and MCF-7 cells after KCNK1 knockdown, LDHA overexpression, or co-transfection of shKCNK1 and LDHA overexpression vectors. **(E)** Transwell experiments with Matrigel were performed to detect the invasion ability of MDA-MB-231 and MCF-7 cells after KCNK1 knockdown, LDHA overexpression, or co-transfection of shKCNK1 and LDHA overexpression vectors. All experiments were triplicated. Data are presented as mean ± SD, analyzed by unpaired two-sided *t* tests. Source data are provided as S1 Data. LDHA, lactate dehydrogenase A.

In addition, MTT and colony formation assays revealed that overexpression of LDHA rescued the inhibitory effect of KCNK1 knockdown on the proliferation of breast cancer cells (Figs 4A, 4B, S4A, and S4B). Wound healing and transwell migration assays also showed that LDHA reversed the inhibitory effect of KCNK1 knockdown on the migration ability of breast cancer cells (Figs 4C, 4D, S4C, and S4D). Transwell invasion assays also demonstrated that overexpression of LDHA rollbacked the attenuated invasion ability of breast cancer cells induced by the knockdown of KCNK1 (Figs 4E and S4E). All these data strongly support that LDHA mediated the functions of KCNK1 on the progression of breast cancer.

We also used siRNA to knockdown the expression of LDHA and the inhibitor GSK to inhibit the activity of LDHA. MTT and colony formation experiments showed that the proliferation ability of cancer cells overexpressing KCNK1 was significantly enhanced. SiRNA or GSK significantly inhibited the proliferation of breast cancer cells. At the same time, using siRNA or GSK to treat cells overexpressing KCNK1 significantly blocked the promoting effect of KCNK1 on the proliferation of cancer cells (S5A, S5B, S6A, S6B, S7A, S7B, S8A, and S8B Figs). Wound healing and transwell migration assays showed that siRNA or GSK treatment of cells significantly reduced the promoting effect of KCNK1 on the migration of cancer cells (S5C, S5D, S6C, S6D, S7C, S7D, S8C, and S8D Figs). Transwell invasion assays showed that siRNA or GSK treatment of cells significantly inhibited the promoting effect of KCNK1 on the invasion of cancer cells (S5E, S6E, S7E, and S8E Figs).

### KCNK1 promotes H3K18 lactylation in breast cancer cells via LDHA

Recently, lactate-driven histone lysine lactylation has been proposed as a novel epigenetic modification involved in transcriptional regulation and activation of gene expression [32]. To examine whether KCNK1 affected pan histone lactylation and H3K18 lactylation (H3K18la) in breast cancer cells, histone was extracted and western blotting assays were performed. The results revealed that overexpression of KCNK1 augmented pan histone and H3K18 lactylation in breast cancer cells, whereas pan histone and H3K18 lactylation were significantly inhibited after KCNK1 knockdown (S9A Fig), suggesting that KCNK1 was indeed involved in regulating histone lactylation in breast cancer cells.

**Fig 5:**
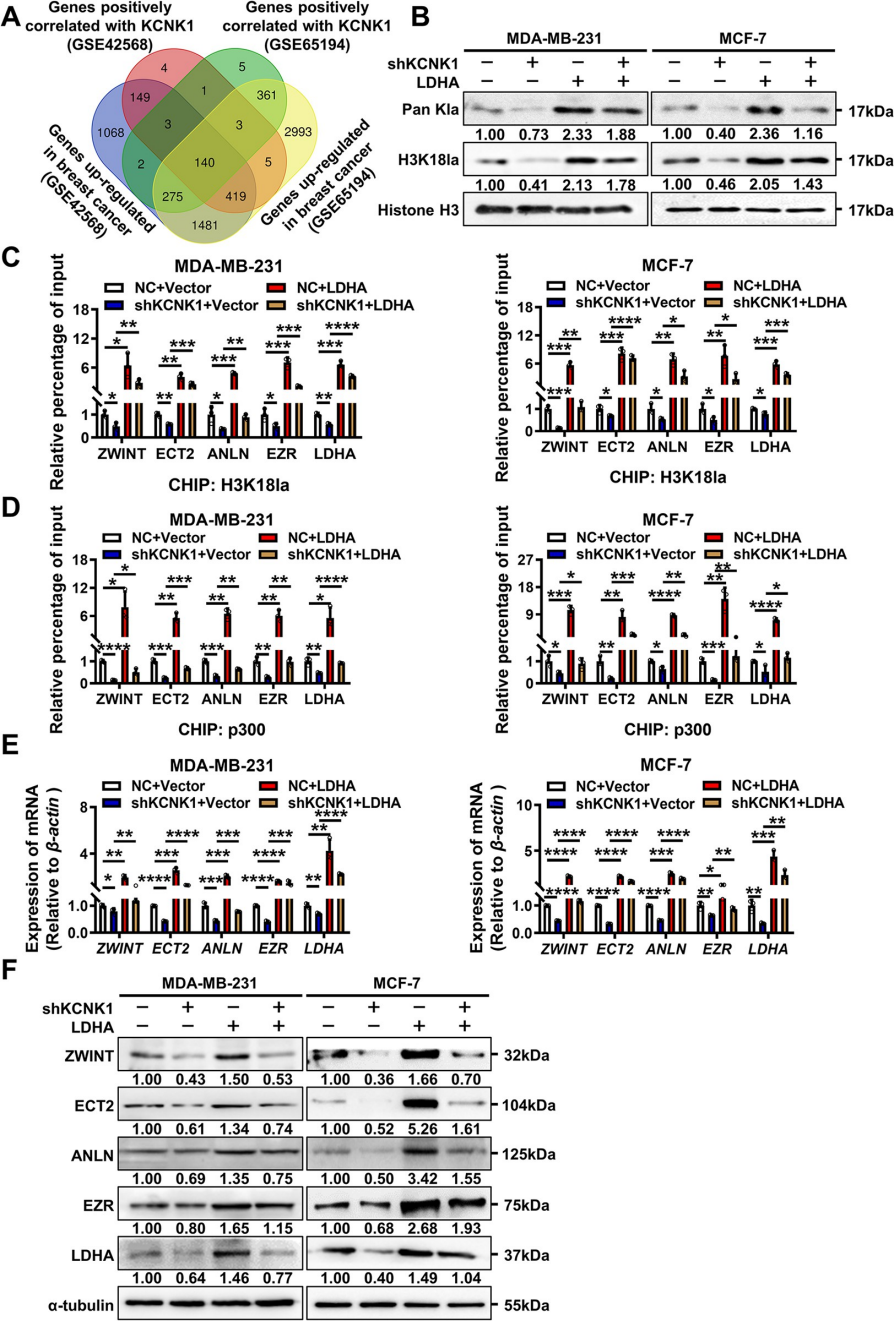
KCNK1 promotes H3K18 lactylation in breast cancer cells via LDHA. **(A)** KCNK1-associated molecules were screened in 2 datasets GSE42568 and GSE65194. **(B)** Western blotting was performed to assess the levels of pan histone and H3K18 site lactylation in breast cancer cells transfected with shKCNK1 or the LDHA overexpression vector. **(C)** ChIP-qPCR assay showed the H3K18la status in ZWINT, ECT2, ANLN, EZR, and LDHA genomic regions was regulated by KCNK1 or LDHA in MDA-MB-231 and MCF-7 cells. **(D)** ChIP-qPCR assay showed the p300 enrichment in ZWINT, ECT2, ANLN, EZR, and LDHA genomic regions were changed in MDA-MB-231 and MCF-7 cells after transfection with the LDHA overexpression vector or along with shKCNK1. **(E)** The expression of ZWINT, ECT2, ANLN, EZR, and LDHA was examined in MDA-MB-231 and MCF-7 cells using qRT-PCR. **(F)** The expression of ZWINT, ECT2, ANLN, EZR, and LDHA was examined in breast cancer cells using western blotting. All experiments were triplicated. Data are presented as mean ± SD, analyzed by unpaired two-sided *t* tests. Source data are provided as S1 Data. LDHA, lactate dehydrogenase A.

To identify potential downstream targets of KCNK1-induced histone lactylation, 2 datasets (GSE42568 and GSE65194) were reanalyzed and 140 genes were up-regulated in breast cancer and also positively correlated with KCNK1 expression (Fig 5A). The H3K18la levels of these 140 genes were analyzed in the published lactylation profile of the H3K18 after inhibition of glycolysis [33]. The H3K18la levels of some genes, including ZW10 Interacting Kinetochore Protein (ZWINT), Epithelial Cell Transforming 2 (ECT2), Anillin Actin Binding Protein (ANLN), Ezrin (EZR) enriched in the proliferation and cytoskeleton-related pathways, were selected for further validation (S4 Table). ChIP-qPCR assays showed that KCNK1 changed the enrichment of the H3K18la signals of ZWINT, ECT2, ANLN, and EZR (S9B Fig). Lactate-driven histone lysine lactylation was catalyzed by p300 [34,35]. ChIP-qPCR experiment showed that KCNK1 promoted the enrichment of p300 on ZWINT, ECT2, ANLN, and EZR, and the enrichment decreased after KCNK1 knockdown (S9C Fig). As expected, both mRNA and protein levels of ZWINT, ECT2, ANLN, and EZR increased significantly after KCNK1 overexpression, while KCNK1 knockdown inhibited the expression of these downstream targets (S9D and S9E Fig).

Interestingly, overexpression of LDHA elevated the intracellular pan histone lactylation and H3K18la modification in breast cancer (S10A Fig). Previous studies have reported that the transcription of LDHA was regulated by H3K18la [32]. Results of ChIP-qPCR indicated that levels of H3K18la on ZWINT, ECT2, ANLN, EZR, and LDHA increased significantly after LDHA overexpression (S10B Fig). The binding levels with p300 also did (S10C Fig). Consistently, qRT-PCR and western blotting analysis demonstrated that the expression levels of ZWINT, ECT2, ANLN, EZR, and LDHA increased significantly after LDHA overexpression (S10D and S10E Fig). These data strongly suggested that LDHA served as a positive feedback driving intracellular pan histone lactylation and H3K18la.

To test if KCNK1 affected the pan histone and H3K18 lactylation through LDHA in breast cancer cells, western blotting assay was employed after co-transfection of LDHA and KCNK1-shRNA vectors in breast cancer cells. The results showed that overexpression of LDHA blocked the inhibitory effect of KCNK1 knockdown on pan histone lactylation and H3K18la (Fig 5B). ChIP-qPCR also showed that overexpression of LDHA blocked the inhibitory effect of KCNK1 knockdown on H3K18la and p300 binding of ZWINT, ECT2, ANLN, EZR, and LDHA in breast cancer cells (Fig 5C and 5D). In addition, results of qRT-PCR and western blotting demonstrated that overexpression of LDHA rescued the expression of downstream targets reduced by knockdown of KCNK1 in breast cancer cells (Fig 5E and 5F).

Taken together, the above results clearly demonstrated that mediated through LDHA, KCNK1 overexpression significantly enhanced cellular aerobic glycolysis, promoted lactate production, augmented histone lactylation, and induced the transcription of downstream targets including LDHA itself, which finally led to the proliferation, invasion, and metastasis of breast cancer cells.

### KCNK1 reduces the stiffness and adhesion of breast cancer cells

**Fig 6:**
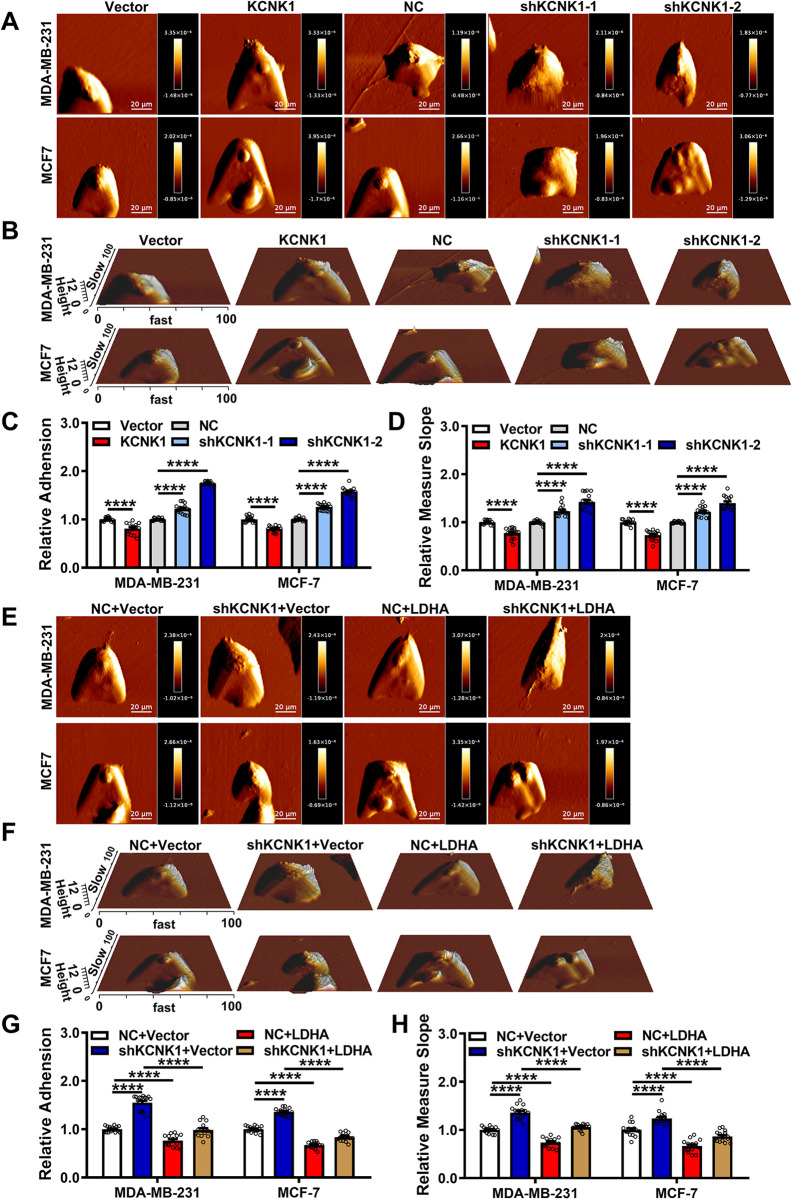
KCNK1 reduces the stiffness and adhesion of breast cancer cells. **(A**, **B)** Representative AFM deflection images (**A**) and 3D height distribution maps (**B**) of MDA-MB-231 cells and MCF-7 cells in the control group, KCNK1 overexpression group, and KCNK1 knockdown group. Cells were fixed in a petri dish and were selected randomly for AFM detection. **(C**, **D)** The adhesion (**C**) and stiffness (**D**) of the scanned cells in the control group, KCNK1 overexpression group, and KCNK1 knockdown group were statistically analyzed and plotted based on the JPK image processing score. Overexpression of KCNK1 decreased the adhesion and stiffness of breast cancer cells, and knockdown of KCNK1 increased the adhesion and stiffness of breast cancer cells. **(E**, **F)** Representative AFM deflection images (**E**) and 3D height distribution maps (**F**) of MDA-MB-231 cells and MCF-7 cells in the control group, KCNK1 knockdown group, LDHA overexpression group, and co-transfection of shKCNK1 and LDHA overexpression vectors group. The cells of each group were fixed in a Petri dish and were selected randomly for AFM detection. **(G**, **H)** The adhesion (**G**) and stiffness (**H**) of scanned cells in the control group, KCNK1 knockdown group, LDHA overexpression group, and co-transfection of shKCNK1 and LDHA overexpression vectors group were statistically analyzed and plotted based on JPK image processing score. Data are presented as mean ± SD, analyzed by unpaired two-sided *t* tests. Source data are provided as S1 Data. AFM, atomic force microscopy; LDHA, lactate dehydrogenase A.

Among the downstream target genes regulated by KCNK1, ANLN and EZR are involved in cytoskeleton assembly, cell adhesion, and motility, so atomic force microscopy (AFM) was employed to assess the biophysical properties of breast cancer cells. The surface adhesion and stiffness were measured from the force curve obtained at each point using AFM. The results showed that overexpression of KCNK1 reduced cell adhesion and stiffness, indicating that cells were more readily detached from their nests and their deformation and invasion abilities were enhanced by KCNK1. In contrast, knockdown of KCNK1 enhanced the adhesion and stiffness of breast cancer cells, indicating an attenuated ability to invasion and metastasis (Fig 6A–6D). In addition, the surface adhesion and stiffness of cells were measured using the AFM in MDA-MB-231 and MCF-7 cells after co-transfection with the KCNK1-shRNA and LDHA overexpression vectors. The data showed that overexpression of LDHA reduced the adhesion and stiffness of breast cancer cells that were enhanced by KCNK1 knockdown (Fig 6E–6H).

To further prove the relationship between KCNK1 and the invasion and migration of breast cancer cells, we conducted a 3D culture experiment. It can be seen that the number of tentacles extended by the cell cluster after overexpressing KCNK1 increased significantly, proving that the cell’s invasion and migration ability increased. After knocking down KCNK1, the number of scattered tentacles of the cells was less than that of the control group, proving that the cell’s invasion ability was weakened (S11A Fig). At the same time, we also performed immunofluorescence on multiple markers that are highly correlated with cell invasion and migration. The experimental results showed that compared with the control group, the aggregation and strength of the cytoskeleton were significantly enhanced after KCNK1 overexpression (S11B Fig), the expression of FAK, RhoA, and Rock1 were increased considerably (S11C–S11E Fig), and the opposite results were obtained after knocking down KCNK1. These results confirmed that KCNK1 can significantly promote the invasion and migration of breast cancer cells.

To investigate whether KCNK1 affects the invasion and migration of breast cancer cells through LDHA, we knocked down KCNK1 in breast cancer cells while overexpressing LDHA. The results showed that the number of pseudopodia in breast cancer cells was lower after knocking down KCNK1, while the number of pseudopodia in cells increased after overexpressing LDHA (S12A Fig). The F-actin structure was looser and the signals of FAK, RhoA, and Rock1 were weaker after knocking down KCNK1. Overexpressing LDHA resulted in a denser F-actin structure and enhanced expression of FAK, RhoA, and Rock1 (S12B–S12E Fig). Overexpressing LDHA in cells with knocked down KCNK1 significantly restored the inhibitory effects caused by knocking down KCNK1 (S12A–S12E Fig). These results indicated that KCNK1 promoted the invasion and migration through LDHA.

### KCNK1 promotes breast cancer cells growth and metastasis in vivo

**Fig 7:**
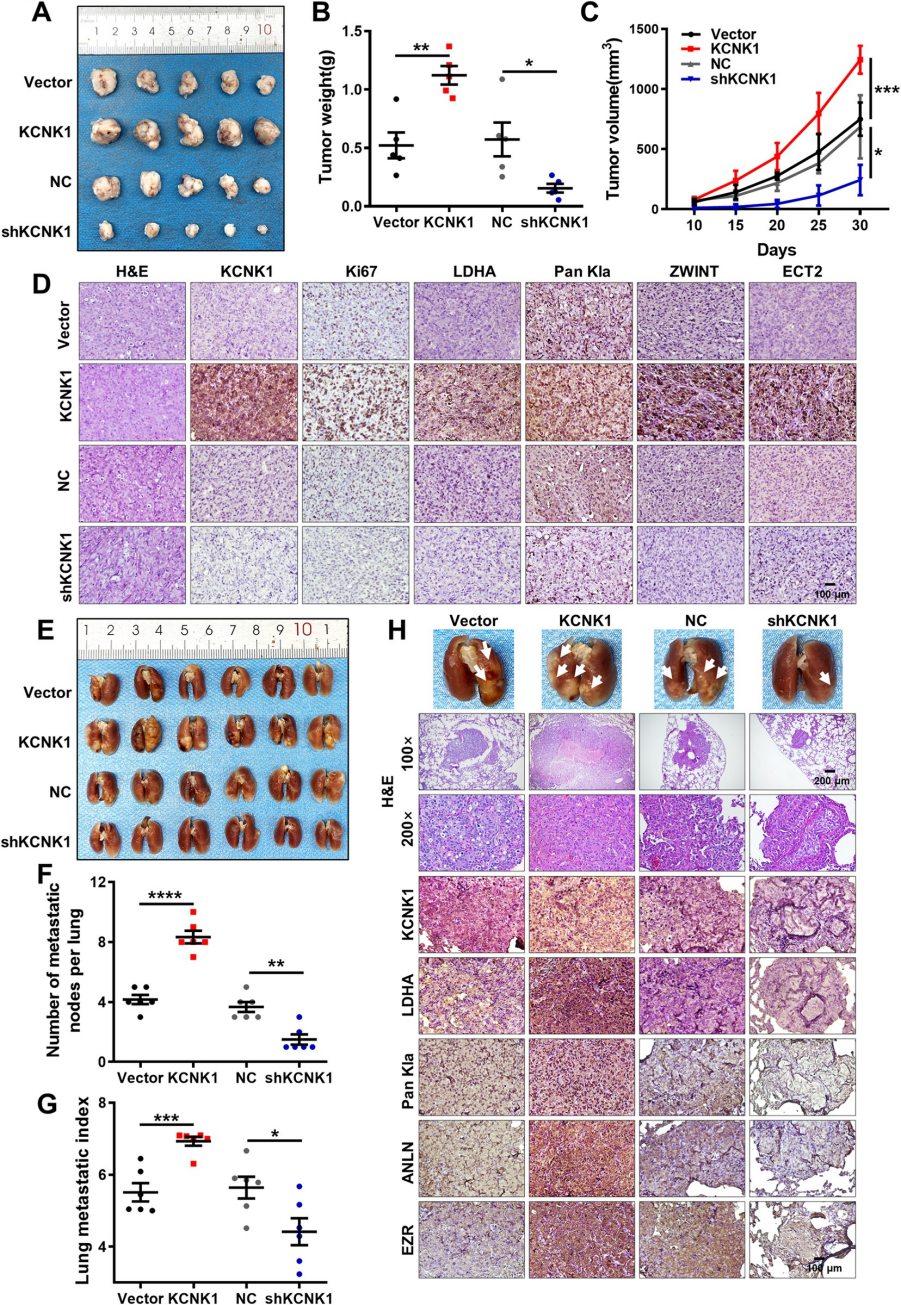
KCNK1 promotes breast cancer cells growth and metastasis in vivo. **(A)** Images of subcutaneous tumors in nude mice. Mice were injected with 2 × 10^6^ MDA-MB-231 cells after knockdown or overexpression of KCNK1. **(B**, **C)** Tumor volumes (**B**) and tumor weights (**C**) were measured for each group (*n* = 5 per group). Data were represented as mean ± SD. **(D)** Representative images of the expression of KCNK1, Ki67, LDHA, Pan Kla, ZWINT, and ECT2 expression in subcutaneous tumors by immunohistochemistry (200× , scale bar: 100 μm). **(E)** Image of visible nodules on the lung surface. **(F)** The number of lung metastatic nodules on each lung surface was quantified. Data were represented as mean ± SD (each point represents 1 mouse; *n* = 6 per group). **(G)** The lung metastasis index was quantified for each mice. Data are expressed as mean ± SD (each point represents 1 mouse; *n* = 6 per group). **(H)** Image of visible nodules on the lung surface and representative images of KCNK1, LDHA, Pan Kla, ANLN, and EZR expression in lung nodules by immunohistochemistry (100× , scale bar: 200 μm; 200× , scale bar: 100 μm). Unpaired two-sided *t* tests were used to analyze the data. Source data are provided as S1 Data. LDHA, lactate dehydrogenase A.

To investigate the effect of KCNK1 on breast cancer cell proliferation and metastasis in vivo, xenograft models were established through inoculating MDA-MB-231 cells after transfecting of the KCNK1 overexpression or shKCNK1 vectors by subcutaneous or tail vein injection, respectively. The subcutaneous tumor model showed that the tumor volume of the nude mice in the KCNK1 overexpression group was larger compared with that of the control group, whereas it was smaller in the KCNK1 knockdown group (Figs 7A–7C and S13A). Immunohistochemical staining showed that expression of Ki-67, LDHA, Pan Kla, ZWINT, and ECT2 in the KCNK1 overexpression group was significantly higher than that in the KCNK1 knockdown group (Figs 7D and S13B). The lung metastasis model showed that metastatic nodules and lung metastatic index were significantly reduced in the KCNK1 knockdown group and increased in the KCNK1 overexpression group, compared with the control group (Fig 7E–7G). HE staining and immunohistochemical staining of lung nodules also revealed that the number of lung metastases and expression of LDHA, Pan Kla, ANLN, and EZR in the KCNK1 overexpression group was significantly higher than that in the KCNK1 knockdown group (Figs 7H and S13C). Taken together, the above results suggested that KCNK1 promoted the proliferation and metastasis of breast cancer in vivo.

### KCNK1 and downstream proteins are positively correlated in clinical breast cancer samples

**Fig 8:**
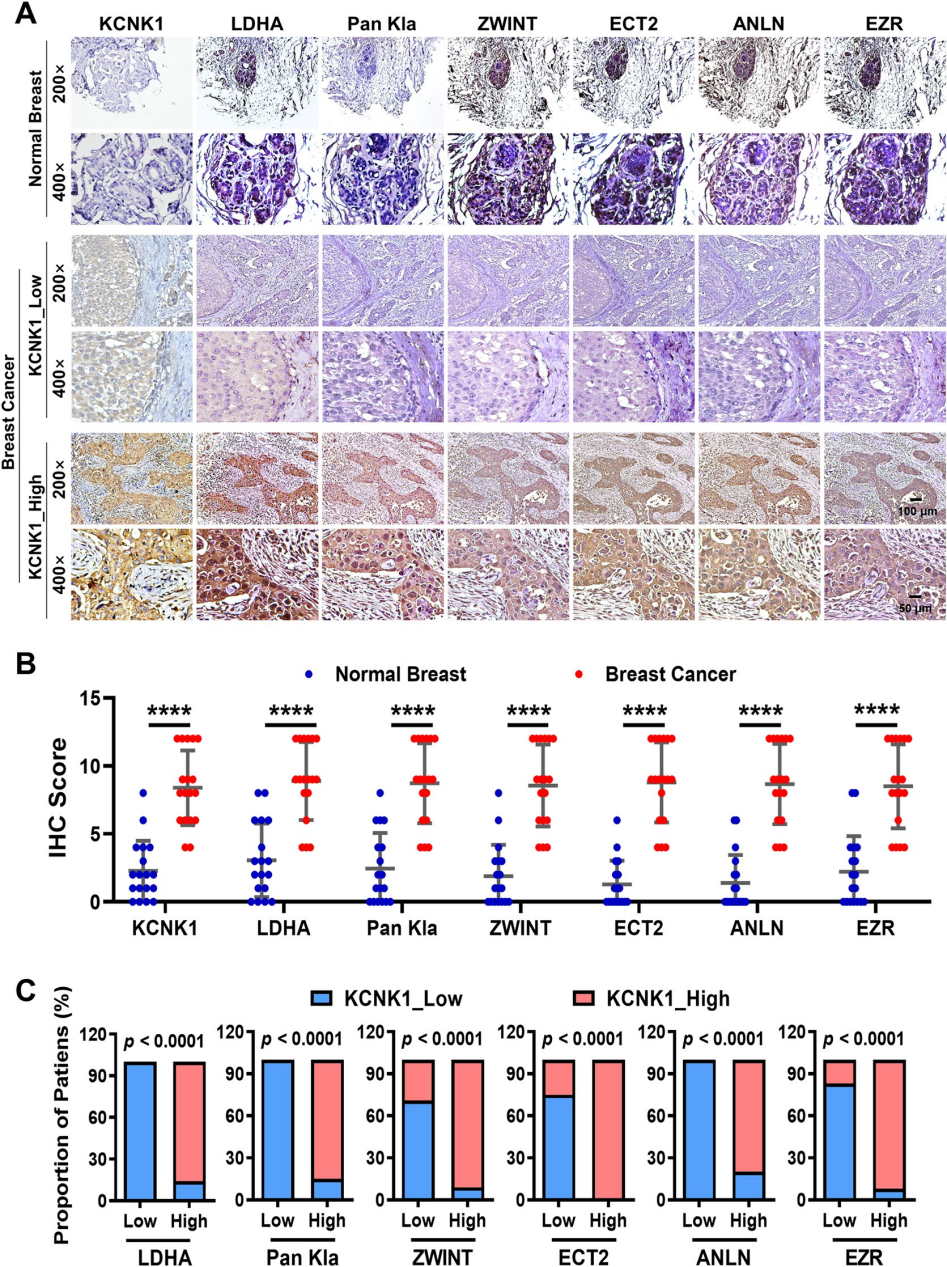
KCNK1 and downstream proteins are positively correlated in clinical breast cancer samples. **(A)** Immunohistochemistry was performed to detect the expression of KCNK1, LDHA, Pan Kla, ZWINT, ECT2, ANLN, and EZR in 18 pairs of breast cancer and adjacent breast epithelial tissues. **(B)** The statistical analysis of KCNK1, LDHA, Pan Kla, ZWINT, ECT2, ANLN, and EZR expression in 18 pairs of breast cancer and adjacent breast epithelial tissues. Unpaired two-sided *t* tests were used to analyze the data. **(C)** The percentage of KCNK1 expression levels in clinical breast cancer samples with high or low expression of LDHA, Pan Kla, ZWINT, ECT2, ANLN, and EZR. Data calculated by F-test. Source data are provided as S1 Data. LDHA, lactate dehydrogenase A.

To assess the expression and correlation among KCNK1, LDHA, Pan Kla, ZWINT, ECT2, ANLN, and EZR, immunohistochemistry was performed using 18 pairs of paraffin-embedded breast cancer and adjacent normal breast tissue samples (S2 Table), the data showed that KCNK1 was highly differentially expressed in breast cancer samples, and the expression of LDHA, Pan Kla, ZWINT, ECT2, ANLN, and EZR were also higher than that in those paired adjacent normal breast tissues (Fig 8A and 8B). KCNK1 expression was positively correlated with that of LDHA, Pan Kla, ZWINT, ECT2, ANLN, and EZR (Fig 8C), reconfirming that KCNK1 played an important role in the proliferation and metastasis of breast cancer.

## Discussion

Potassium channels are one of the most abundant ion channels in cells, which can regulate the permeability of potassium ions, maintain the membrane potential, and modulate osmotic pressure upon environmental cues [36,37]. It has been reported that KCNJ10 [38], KCNA5 [39], and KCNN4 [40] can affect the proliferation and invasive ability of gliomas and glioblastomas by altering the cell membrane potential in excitatory cell-derived tumors. Meanwhile, it has been shown that KCNH1 and KCNH2 accelerate the proliferation of non-excitable cell-derived cancer cells, which has nothing to do with their ion conduction function [41,42]. In addition, multiple potassium channels synergistically regulate cell motility through communicating with other signaling molecules [43,44]. However, whether KCNK1 executes its biological function through non-ion channel function remains elusive. In this study, we identified for the first time that the expression of KCNK1 was remarkably up-regulated in breast cancer, which was closely associated with poor prognosis of breast cancer patients (Fig 1). KCNK1 promoted proliferation, invasion, and metastasis of breast cancer cells in vitro and vivo. Nevertheless, the proliferation, invasion, and migration of breast cancer were not affected after treatment with KCNK1 inhibitor, indicating that KCNK1 regulated breast tumorigenesis through non-ion channel function (Fig 2).

Further studies unexpectedly revealed that KCNK1 contributed to the malignant phenotype of breast cancer by binding to and activating LDHA (Figs 3 and 4). LDHA, a crucial rate-limiting enzyme in the glycolytic pathway, plays a vital role in facilitating the malignant process of tumors by regulating their energy metabolism [45]. Notably, its catalytic product, lactate, the final product of cellular glycolysis, not only fulfills important functions in various physiological processes but also exhibits an intimate association with tumorigenesis [46–50]. In 2019, Zhao and colleagues first reported that lactate was the crucial raw material for lactylation of histone lysines, a novel posttranslational protein modification, which activated gene expression mainly through lactylating H3K18 [32,51–54]. Subsequently, p300 was identified as the major player in the histone lactylation process, catalyzing the transfer of lactyl group to histone and facilitating H3K18la modification [34,35,52].

The breast cancer expression profiling datasets GSE42568 and GSE65194 were then analyzed and it was found that 140 genes were up-regulated and positively correlated with KCNK1 expression in breast cancer cells. Meanwhile, Renbing Jia and colleagues reported the lactylation profile of the H3K18 after inhibition of glycolysis [33]. Upon cross examination, we selected the top 20 genes with the most dramatic differences in H3K18la modification after glycolysis inhibition from the above 140 candidate genes. Of these 20 genes, ZWINT, ECT2, ANLN, and EZR were linked to cell proliferation, invasion, and metastasis. We demonstrated experimentally that the expression of ZWINT, ECT2, ANLN, and EZR was up-regulated upon overexpression of KCNK1, and ChIP-qPCR confirmed that overexpression of KCNK1 enhanced the H3K18la modification of these 4 genes, along with significant enrichment of p300 on these genes. Our data further demonstrated that KCNK1 induced the metabolic reprogramming of breast cancer cells via LDHA, which accelerated lactate production, catalyzed lactylation modifications of a series of downstream target genes, and thus promoted the malignant progression of breast cancer (Figs 5 and 6).

The kinetochore protein ZWINT is required for kinetochore formation and cell division [55]. ECT2 is expressed in a cell cycle-dependent manner and has an essential role in cytokinesis [56,57]. ANLN is an actin-binding protein that functions in cell growth and migration [58,59]. EZR acts as an intermediate between the actin cytoskeleton and the plasma membrane, which plays a key role in cell adhesion and migration [60,61]. ANLN and EZR have been demonstrated to play crucial roles in cell migration. They exert their influence by regulating actin, which governs the dynamic assembly of the cytoskeleton. This, in turn, affects the biophysical properties of tumor tissues, a critical factor in the malignant transformation and metastasis of tumors [62–66]. In fact, the biomechanical properties of tumor cells are also important factors that affect the biophysical properties of tumor tissues and are essential for the malignant transformation and metastasis of tumors [67,68]. For example, studies on melanoma and breast cancer cells have found that as the metastatic potential increases, cell stiffness also increases [69,70]. Our previous studies have also shown that LINC00472 can inhibit the migration and invasion of lung adenocarcinoma by regulating cell stiffness [71]. Numerous studies have shown that reduced tumor cell stiffness tends to give the cells a greater capacity for deformation and decreased cell adhesion facilitates their migration across various barriers and detachment from surrounding tissues to acquire a higher metastatic capacity [72–74]. AFM (atomic force microscopy) by micro-cantilever scanning of samples is a reliable quantitative measurement method for cell mechanics studies [75,76]. The cytoskeleton is an important participant in biophysics and is the main object of AFM detection. Extensive AFM imaging studies have shown that the aggregation and assembly of microfilaments, microtubules, and intermediate filaments are essential for the physical and dynamic properties of cells [77]. They affect cell adhesion and mechanical signal transduction [78]. Treatment with cell relaxin can block cytoskeleton polymerization and affect multiple biomechanical parameters of cells [79–81]. Overall, in a variety of cancer cell types, biomechanical measurements can be considered as biomarkers of cell transformation and tumor progression [82–84]. We detected by AFM that KCNK1 overexpression made cells softer and weakened the adhesion, which might be the reason for the enhanced invasion and metastasis of breast cancer cells (Fig 6). Our study revealed for the first time the relationship between the dysregulation of potassium channels and the biophysical properties of breast cancer cells and their malignant progression.

Importantly, we discovered that LDHA was also one of the downstream target genes undergoing H3K18la modifications and was regulated by KCNK1 (Fig 5). KCNK1 binds to and activates LDHA, promotes lactate production, and catalyzes H3K18la modification of target genes in breast cancer cells, and also raises the level of histone lactylation modification of LDHA itself, which further sustained LDHA expression in a positive feedback loop. This positive feedback mechanism may be responsible for the persistent enhanced lactate production in breast cancer cells with high KCNK1 expression, which consequentially promoted breast carcinogenesis (Figs 7 and 8). But the way in which KCNK1 interacts with LDHA, especially on the 3D level, and how this interaction affects the function and activity of LDHA, warrant further investigation.

In conclusion, in this work, we demonstrate for the first time that the potassium channel protein KCNK1 is highly differentially expressed in breast cancer and promotes proliferation, invasion, and metastasis of breast cancer in a non-ion channel-dependent manner. Unexpectedly, KCNK1 binds to and activates LDHA, facilitates the process of glycolysis, catalyzes a new epigenetic modification–histone lysine lactylation, and stimulates the expression of a series of downstream genes related to cell proliferation, invasion, and metastasis, resulting in change of biophysical properties of breast cancer cells. Importantly, activated LDHA serves as a positive feedback, thereby sustaining the malignant progression of breast cancer. During the development of malignant tumors, the acquisition of enhanced proliferative, invasive, and metastatic capacities is necessary for the malignant transformation of epithelial cells. This is a very complex biological change and numerous genes may play important biological functions. However, whether KCNK1 induces malignant transformation of breast cells, from normal cells to malignant transformation, still needs further in-depth and extensive research. KCNK1 and its downstream genes may serve as new molecular markers for the initial diagnosis and clinical prognosis of breast cancer. Further insight into the KCNK1-LDHA pathway should shed light on the design of new targeted drugs for breast cancer treatment.

## Methods

### Gene expression data and clinical samples

Two breast cancer gene expression datasets (GSE42568 and GSE65194) were downloaded from the Gene Expression Omnibus (GEO) database and analyzed using the Significance Analysis of Microarrays (SAM) software (S1 Table).

A total of 174 breast cancer tissues and 27 normal breast tissues were used to assess the expression of KCNK1 by immunohistochemistry (S2 Table), and paraffin-embedded samples from 18 paired breast cancer and adjacent breast tissues were used to detect the expression of LDHA, Pan Kla, ZWINT, ANLN, ECT2, and EZR by immunohistochemistry (S2 Table). All the clinical tissue samples were provided by Xiangya Second Hospital of Central South University. The use of all samples was authorized by the Ethics Committee of Central South University (license number 2021-CR0052), and informed consent was obtained from all patients.

### Cell culture, plasmids, and transfection

MDA-MB-231 and MCF-7 were cultured in complete Dulbecco’s modified Eagle’s medium (DMEM) supplemented with 10% fetal bovine serum (FBS) under 5% CO_2_ at 37°C, and stored in liquid nitrogen with serum-free cell cryopreservation solution (New Cell & Molecular Biotech, China).

The full-length KCNK1 was inserted into the KpnI and XbaI sites of pCMV3-C-FLAG vector (Sino Biological Inc., China) to obtain the overexpression construct. A shRNA fragment was inserted into the EcoRI and BamHI sites of PLVshRNA-EGFP (2A) Puro vector (Inovogen, China) for the production of the shKCNK1 lentiviral particles (S5 Table). The control and siRNA targeting LDHA were purchased from RiboBio Co., Ltd. (Guangzhou, China). Neofect transfection reagent (Invitrogen, United States of America) was used to transfect the KCNK1-overexpression vector or the control empty vector in OptiDMEM medium (BasalMedia, China). Alternatively, HEK293T cells were transfected with either the shRNA or the scramble control vector. The culture medium containing viral particles was harvested 60 h later and was used to infect breast cancer cell lines and then selected with puromycin. Hiperfect (Qiagen, Hilden, Germany) was used to transfect siRNA. The LDHA inhibitor GSK2837808A (MedChem Express, USA) was used in breast cancer cells.

### RNA isolation and qRT-PCR

The cellular RNA was extracted using Trizol reagent (Invitrogen, USA) and reverse transcribed into cDNA by Quantscript RT kit (ABM, Canada). A 5× All-In-One MasterMix kit (ABM, Canada) was used for quantitative real-time PCR. A CFX96 real-time PCR detection system (Bio-Rad, USA) was used to detect the relative expression level of genes. The primers used are listed in S5 Table.

### Western blotting

Breast cancer cells were lysed on ice in RIPA buffer (Beyotime, China). The protein concentration was determined by BCA protein assay kit (Pierce, USA). A total of 50 μg of cell lysate was separated by 10% sodium dodecyl sulfate-polyacrylamide gel electrophoresis (SDS-PAGE), transferred onto a polyvinylidene fluoride (PVDF) membrane (Millipore, USA), and blocked with 5% skim milk for 2 h at room temperature. The membrane was then incubated with primary antibodies at 4°C overnight and washed with 1× PBST (repeated 3 times) the next day. After that, the membranes were incubated with horseradish peroxidase (HRP)-conjugated secondary antibodies at room temperature for 2 h and the signals were detected with ECL reagent. The antibodies used are listed in S6 Table.

### MTT assay

A total of 800 cells per well were inoculated into 96-well plate. Cells were incubated with 0.5 mg/ml filtered sterile MTT (Beyotime, China) at 37°C for 4 h at indicated time point. Then, the media was removed and replaced with 200 μl DMSO. The absorbance of the sample at 490 nm was detected with a multimode microplate reader.

### Colony formation assay

Cells were inoculated in 12-well plate with 1,500 cells per well. Cell colonies were fixed with 4% paraformaldehyde for 15 min when they were just about to visible by naked eye (no less than 50 cells per clone) and then visualized by crystal violet staining.

### Transwell assay

DMEM medium supplemented with 20% FBS was added to the bottom chambers, and then cells suspended in the DMEM medium were seeded into the top chambers which were coated with Matrigel. The plate was then incubated at 37°C until cells reached the bottom of the plate. The plate was then removed from the chamber, washed twice with saline, fixed with 4% paraformaldehyde, and stained with crystal violet. The Matrigel and the cells on the upper surface of the chamber were then wiped off. Cells on the bottom of the chamber were counted under an inverted phase-contrast microscope.

### Wound healing assay

Cells in 6-well plate with 80% to 90% confluence were wounded by using a sterilized pipet tip to make a straight scratch. Cells were further incubated in DMEM medium with 1% FBS, and 5% hydroxyurea was added to inhibit cell division. Images were captured at 0 h, 12 h, and 24 h after wounding until fully healed.

### Glycolysis assay

For the glycolysis assay, a Glycolysis Stress Test Kit (Agilent, USA) was used to measure the extracellular acidification rate following the manufacturer’s instructions (Seahorse Bioscience, USA).

### Immunofluorescence

Cells were inoculated onto Cover Glasses (NEST Biotechnology Co. Ltd, China), left to stretched, fixed in 4% paraformaldehyde for 20 min, penetrated with 0.25% Triton X-100, and blocked in 5% bovine serum albumin for 1 h. Then, cells were incubated with primary antibodies at 4°C overnight and exposed to fluorochrome-labeled second antibodies for 1 h at room temperature. After staining with DAPI for 5 min, cells were imaged under confocal laser scanning microscope (PerkinElmer, USA). The antibodies used are listed in S6 Table.

### Measurement of LDH activity and lactate production

CheKine Lactate Dehydrogenase (LDH) Colorimetric Assay Kit (Abbkine Scientific, USA) and CheKine Lactate Assay Kit (Abbkine Scientific, USA) were used to measure the LDH activity and lactate production.

### Histone extraction and chromatin immunoprecipitation assay (ChIP)

EpiQuik Total Histone Extraction Kit (Epigentek, USA) was used to isolate histones from cells following the manufacturer’s instructions.

For ChIP assay, cells were fixed with 1% formaldehyde for 10 min and the fixation was stopped with 0.125 M glycine. Then, the cell lysis buffer was added and the samples were sonicated to generate 200 to 1,000 bp DNA fragments. The resulting cell lysates were immunoprecipitated using indicated antibodies and analyzed via ChIP-qPCR. The primers used are shown in S5 Table.

### Atomic force microscopy (AFM)

Single breast cancer cell was measured in QI mode by using atomic force microscope JPK NanoWizard 4 XP BioScience (JPK Instruments, Germany). The silicon nitride V-shaped probe was produced by AppNano (California, USA) with a cantilever length of 100 μm and a spring constant of 0.292 N/m. Indentation was carried out at a loading and retraction speed of approximately 3 μm/s. Cells adhered to the culture dish were washed 3 times with PBS and then fixed with 2% glutaraldehyde for 45 s, followed by fixation with 4% polymethanol for 20 min. Finally, cells were washed more than 6 times with PBS, then covered with an appropriate amount of PBS and scanned with AFM. To better simulate the physiological deformation of the cells, the indentation depth was chosen to be at least 1 mm. Images were analyzed using the JPK software.

### Animal experiments

Four-week-old female BALB/c nude mice were purchased from the Experimental Animal Center of Central South University (Changsha, China) and housed in an SPF-free barrier environment. All animal experiments were approved by the Institutional Animal Care and Use Committee (IACUC) of Central South University (No.2021sydw0097).

For subcutaneous tumor model, nude mice were randomly assigned into 4 groups (*n* = 5). Then, each nude mouse was subcutaneously injected with 2 × 10^6^ MDA-MB-231 cells. Tumor growth was monitored every 3 days. Tumor size was assessed by measuring the largest perpendicular diameters, and tumor volume was calculated by: V = 1/2 × (length) × (width) × (width). One month later, mice were euthanized and the tumors were removed and weighed. For lung metastasis model, nude mice were randomly divided into 4 groups (*n* = 6). Each nude mouse was injected with 2 × 10^6^ MDA-MB-231 cells via the tail vein. Eight weeks later, nude mice were sacrificed. Lungs were removed, weighed, imaged, and the number of nodules on the surface of the lungs was tabulated to assess tumor metastasis. Lung tissues were then subjected to gradient dehydration, sectioned, embedded in paraffin, and then stained with HE for histological examination.

### Immunohistochemistry and hematoxylin-eosin staining (HE)

For immunohistochemistry, Tissues were heated at 65°C for 3 h, then dewaxed and hydrated, and the samples were subjected to EDTA-mediated high-temperature antigen retrieval. The samples were then incubated overnight at 4°C with primary antibodies. After washing 3 times, the second antibody was then added and incubated at room temperature for 2 h. The staining was scored according to the staining intensity and the distribution of stained cells. Distribution was evaluated as none (0), ≤10% (1), 10%–50% (2), 50%–80% (3), and >80% (4). The intensity was evaluated as none (0), weak (1), moderate (2), and strong (3). The final staining scores were calculated as the product of staining intensity and the percentage of stained cells. The antibodies used are listed in S6 Table. For HE staining, paraffin embedded mouse tissue sections were stained with hematoxylin solution for nuclei and eosin solution for cytoplasm.

### Statistical analysis

Statistical analyses were performed using GraphPad Prism 8.0 software. Differences between groups were analyzed using the Student’s *t* test. A two-tailed value of *p* < 0.05 was considered statistically significant. *, *p* < 0.05; **, *p* < 0.01; ***, *p* < 0.001; ****, *p* < 0.0001.

### Ethics approval and consent to participate

All animal protocols were approved by the Institutional Animal Care and Use Committee at Central South University (No.2021sydw0097).

## Supporting information

S1 Graphical Abstract Schematic diagram of the molecular mechanism of KCNK1 in breast cancer progression. KCNK1 binds to and activates LDHA to accelerate cellular glycolysis and lactate production, which enhances the level of histone lactylation modification of a series of downstream targets LDHA, ZWINT, ECT2, ANLN, and EZR, and promotes genes transcription and reduces stiffness and adhesion of breast cancer cells, all these ultimately lead to the proliferation and metastasis of breast cancer cells. Notably, KCNK1-activated LDHA serves as a positive feedback to sustain this vicious loop.
(TIF)

S1 Raw Images This file contains all raw images of western blot results. (PDF)

S1 Data Excel spreadsheet containing, in separate sheets, the underlying numerical data for figure panels. (XLSX)

S1 Fig Screening of differentially expressed ion channels in breast cancer. **(A)** Schematic workflow for identifying and validating the differentially expressed ion channels in 2 breast cancer datasets. **(B)** The expression of CLCN3, CACNB3, and ANO6 was analyzed in normal breast tissues and breast cancer tissues based on 2 datasets GSE42568 and GSE65194. **(C**, **D)** Kaplan–Meier analysis showed the effect CLCN3, CACNB3, and ANO6 expression on the overall survival (**C**) and relapse-free survival (**D**) of breast cancer patients in GSE42568. **(E)** The expression of KCNK1 was analyzed in different breast cancer subtypes according to dataset GSE65194. **(F)** The expression of KCNK1 was analyzed in breast cancer tissues based on dataset GSE42568. And *p*-values were calculated by unpaired two-sided *t* test in **E** and **F**. Source data are provided as S1 Data.
(TIF)

S2 Fig KCNK1 promotes proliferation, migration, and invasion of breast cancer cells. **(A**, **B)** The overexpression and knockdown efficiencies of KCNK1 were detected by qRT-PCR; (**A**) and western blotting assays (**B**) in MDA-MB-468 and T47D cells. **(C–G)** MTT assays (**C**), clone formation assays (**D**), wound healing (**E**), Transwell experiments without (**F**) or with Matrigel (**G**) were performed after KCNK1 overexpression or shRNAs knockdown in MDA-MB-468 and T47D cells. All experiments were performed in at least triplicate samples. Data were presented as mean ± SD, unpaired two-sided *t* tests were used to analyze the data. Source data are provided as S1 Data.
(TIF)

S3 Fig Quinine has no role in KCNK1 promotes proliferation, migration, and invasion of breast cancer cells. **(A**) Images of the medium color change the overexpression/knockdown of KCNK1 or treatment with Quinine. **(B)** The lactate production in breast cancer cells was detected after overexpression or knockdown of KCNK1. **(C**, **D)** The proliferation ability of MDA-MB-231 and MCF-7 cells were detected by MTT assays (**C**) and clone formation assays (**D**) after altering KCNK1 expression or treatment with Quinine. **(E)** Wound healing experiments were performed to detect the migration ability of MDA-MB-231 cells after altering KCNK1 expression or treatment with Quinine. **(F)** Transwell experiments without Matrigel were performed to detect the migration ability of MDA-MB-231 and MCF-7 cells after altering KCNK1 expression or treatment with Quinine. **(G)** Transwell experiments with Matrigel were performed to detect the invasion ability of MDA-MB-231 and MCF-7 cells after altering KCNK1 expression or treatment with Quinine. **(H)** Resting potential of breast cancer cells after overexpression or knockdown KCNK1 was measured. **(I)** Resting potential of breast cancer cells before and after quinine treatment was measured. All experiments were performed in at least triplicate samples. Data were presented as mean ± SD, unpaired two-sided *t* tests were used to analyze the data. Source data are provided as S1 Data.
(TIF)

S4 Fig KCNK1 promotes the proliferation, migration, and invasion of breast cancer cells via LDHA. **(A–E)** MTT assays (**A**), clone formation assays (**B**), wound healing (**C**), Transwell experiments without (**D**) or with Matrigel (**E**) were performed after KCNK1 knockdown, LDHA overexpression, or co-transfection of shKCNK1 and LDHA overexpression vectors in MDA-MB-468 and T47D cells. All experiments were performed in at least triplicate samples. Data were presented as mean ± SD, unpaired two-sided *t* tests were used to analyze the data. Source data are provided as S1 Data.
(TIF)

S5 Fig Knockdown LDHA reversed the promotion of breast cancer cells phenotype by KCNK1 overexpression. **(A–E)** MTT assays (**A**), clone formation assays (**B**), wound healing (**C**), Transwell experiments without (**D**) or with Matrigel (**E**) were performed after KCNK1 overexpression, LDHA knockdown, or co-transfection of siLDHA and KCNK1 overexpression vectors in MDA-MB-231 and MCF-7 cells. All experiments were performed in at least triplicate samples. Data were presented as mean ± SD, unpaired two-sided *t* tests were used to analyze the data. Source data are provided as S1 Data.
(TIF)

S6 Fig Knockdown LDHA reversed the promotion of KCNK1 overexpression on breast cancer cells phenotype. **(A–E)** MTT assays (**A**), clone formation assays (**B**), wound healing (**C**), Transwell experiments without (**D**) or with Matrigel (**E**) were performed after KCNK1 overexpression, LDHA knockdown, or co-transfection of siLDHA and KCNK1 overexpression vectors in MDA-MB-468 and T47D cells. All experiments were performed in at least triplicate samples. Data were presented as mean ± SD, unpaired two-sided *t* tests were used to analyze the data. Source data are provided as S1 Data.
(TIF)

S7 Fig LDHA inhibitor reversed the promotion of breast cancer cells phenotype by KCNK1 overexpression. **(A–E)** MTT assays (**A**), clone formation assays (**B**), wound healing (**C**), Transwell experiments without (**D**) or with Matrigel (**E**) were performed after KCNK1 overexpression, treatment with GSK, or treatment with GSK in parallel with KCNK1 overexpression in MDA-MB-231 and MCF-7 cells. All experiments were performed in at least triplicate samples. Data were presented as mean ± SD, unpaired two-sided *t* tests were used to analyze the data. Source data are provided as S1 Data.
(TIF)

S8 Fig LDHA inhibitor reversed the promotion of KCNK1 overexpression on breast cancer cells phenotype. **(A–E)** MTT assays (**A**), clone formation assays (**B**), wound healing (**C**), Transwell experiments without (**D**) or with Matrigel (**E**) were performed after KCNK1 overexpression, treatment with GSK, or treatment with GSK in parallel with KCNK1 overexpression in MDA-MB-468 and T47D cells. All experiments were performed in at least triplicate samples. Data were presented as mean ± SD, unpaired two-sided *t* tests were used to analyze the data. Source data are provided as S1 Data.
(TIF)

S9 Fig KCNK1 promotes H3K18 lactylation of breast cancer cells. **(A)** The pan histone and H3K18 site lactylation levels were detected in breast cancer cells after overexpression or knockdown of KCNK1 by western blotting assays. **(B)** ChIP-qPCR assay showed the H3K18la status in ZWINT, ECT2, ANLN, EZR, and LDHA genomic regions in MDA-MB-231 and MCF-7 cells after overexpression or knockdown of KCNK1. **(C)** ChIP-qPCR assay showed the p300 enrichment in ZWINT, ECT2, ANLN, EZR, and LDHA genomic regions in MDA-MB-231 and MCF-7 cells after overexpression or knockdown of KCNK1. **(D)** The expression of ZWINT, ECT2, ANLN, EZR, and LDHA was examined in MDA-MB-231 and MCF-7 cells using qRT-PCR after overexpression or knockdown of KCNK1. **(E)** The expression of KCNK1 downstream targets was examined in breast cancer cells using western blotting after KCNK1 overexpression or knockdown. All experiments were performed in at least triplicate samples. Data were presented as mean ± SD, unpaired two-sided *t* tests were used to analyze the data. Source data are provided as S1 Data.
(TIF)

S10 Fig LDHA promotes H3K18 lactylation in breast cancer cells. **(A)** Western blotting was used to detect pan histone and H3K18 site lactylation levels in LDHA overexpressed breast cancer cells. **(B)** ChIP-qPCR assay showed the H3K18la status in ZWINT, ECT2, ANLN, EZR, and LDHA genomic regions in MDA-MB-231 and MCF-7 cells after overexpression of LDHA. **(C)** ChIP-qPCR assay showed the p300 enrichment in ZWINT, ECT2, ANLN, EZR, and LDHA genomic regions in MDA-MB-231 and MCF-7 cells after overexpression of LDHA. **(D)** Expression of ZWINT, ECT2, ANLN, EZR, and LDHA was detected in MDA-MB-231 and MCF-7 cells after overexpression of LDHA using qRT-PCR. **(E)** Expression of ZWINT, ECT2, ANLN, EZR, and LDHA was detected in breast cancer cells after overexpression of LDHA using western blotting. All experiments were performed in at least triplicate samples. Data were presented as mean ± SD, unpaired two-sided *t* tests were used to analyze the data. Source data are provided as S1 Data.
(TIF)

S11 Fig KCNK1 overexpression is significantly associated with breast cancer cells invasion and metastasis markers. **(A)** A 3D culture model formed in Matrigel after overexpression or knockdown of KCNK1 in MDA-MB-231 cells. White arrows indicate scattered protrusions formed on the spheroid surfaces. And *p*-values were calculated by unpaired two-sided *t* test. **(B–E)** The F-actin, FAK, RhoA, and Rock1 protein was detected by IF after KCNK1 overexpression or knockdown. DAPI: blue; F-actin, FAK, RhoA, and Rock1: red.
(TIF)

S12 Fig KCNK1 affects breast cancer cell phenotype via LDHA. **(A)** A 3D culture model formed in Matrigel after KCNK1 knockdown, LDHA overexpression, or co-transfection of shKCNK1 and LDHA overexpression vectors in MDA-MB-231 cells. White arrows indicate scattered protrusions formed on the spheroid surfaces. And *p*-values were calculated by unpaired two-sided *t* test. **(B–E)** The F-actin, FAK, RhoA, and Rock1 protein was detected by IF after KCNK1 knockdown, LDHA overexpression, or co-transfection of shKCNK1 and LDHA overexpression vectors. DAPI: blue; F-actin, FAK, RhoA, and Rock1: red.
(TIF)

S13 Fig KCNK1 promotes downstream genes expression in vivo. **(A)** Images of subcutaneous tumorigenic mice after injection with MDA-MB-231 cells transfected with the KCNK1 overexpression or shRNA vectors. **(B)** The statistical analysis of KCNK1, Ki67, LDHA, Pan Kla, ZWINT, and ECT2 expression in subcutaneous tumors tissues. **(C)** The statistical analysis of KCNK1, LDHA, Pan Kla, ANLN, and EZR expression in lung metastatic nodules. Data were presented as mean ± SD, unpaired two-sided *t* tests were used to analyze the data. Source data are provided as S1 Data.
(TIF)

S1 Table The information of 2 GEO datasets. (XLSX)

S2 Table Clinicopathological data and their proteins expression levels measured by immunohistochemistry. (XLSX)

S3 Table The top 20 of the potential interacting proteins of KCNK1 in MDA-MB-231 cells after overexpression of KCNK1 identified by the LC-MS/MS spectrometry. (XLSX)

S4 Table The top 20 of the potential H3K18la-modified genes regulated by KCNK1. (XLSX)

S5 Table List of shRNAs, qRT-PCR, and ChIP-qPCR primers sequences. (XLSX)

S6 Table List of antibodies for immunohistochemistry, western blotting, and immunofluorescence. (XLSX)

## Abbreviations

AFM: atomic force microscopy
ChIP: chromatin immunoprecipitation assay
DMEM: Dulbecco’s modified Eagle’s medium
ECAR: extracellular acidification rate
ER: estrogen receptor
FBS: fetal bovine serum
GEO: Gene Expression Omnibus
HRP: horseradish peroxidase
LDHA: lactate dehydrogenase A
PR: progesterone receptor
PVDF: polyvinylidene fluoride
SAM: Significance Analysis of Microarrays
SDS-PAGE: sodium dodecyl sulfate-polyacrylamide gel electrophoresis
