# Supplementary figures and images for: KCNK1 promotes proliferation and metastasis of breast cancer cells by activating lactate dehydrogenase A (LDHA) and up-regulating H3K18 lactylation
Source: PLoS Biol. 2024 Jun 21;22(6):e3002666. doi: 10.1371/journal.pbio.3002666 (PMC11192366; doi:10.1371/journal.pbio.3002666)

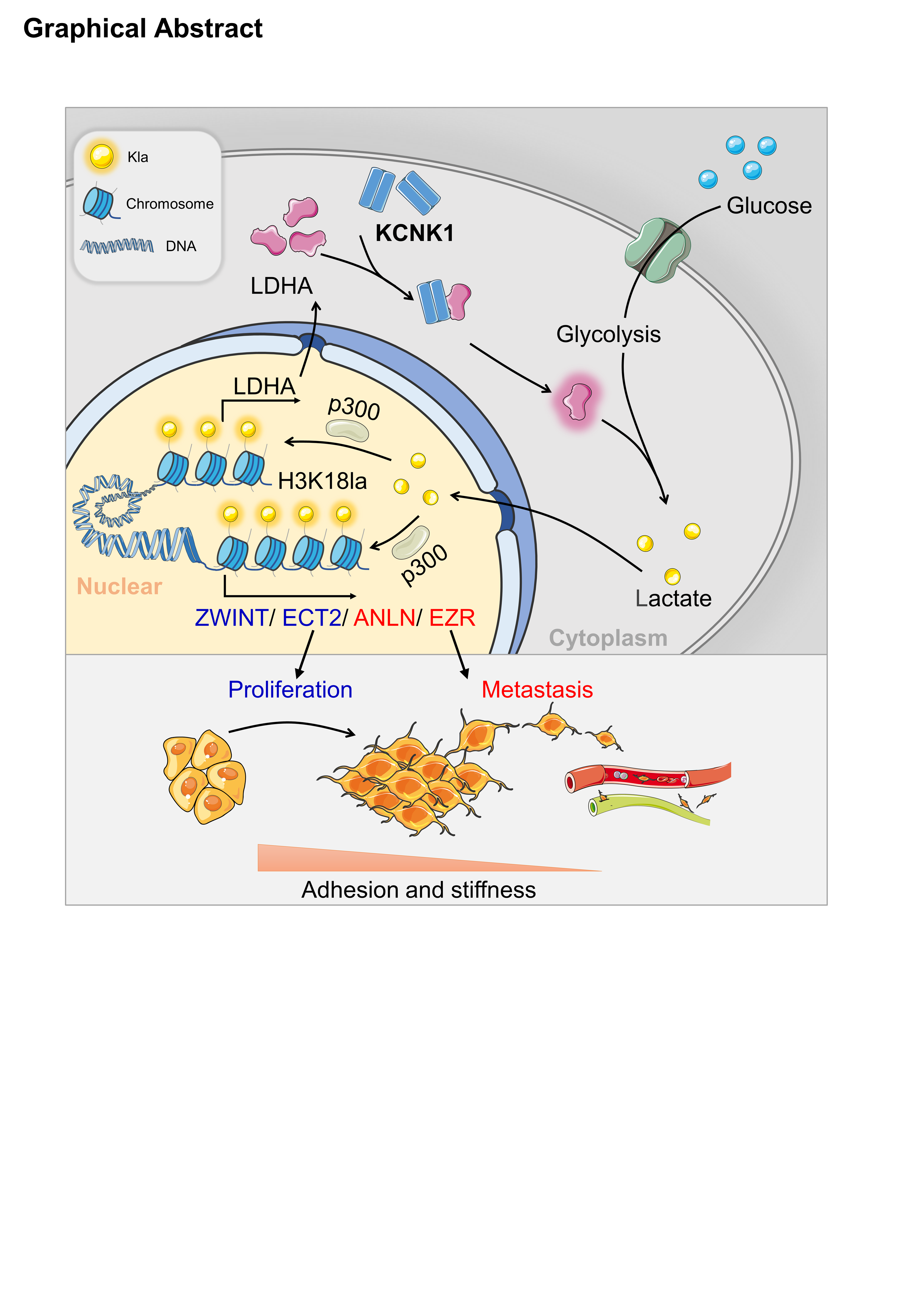

Supplement: S1 Graphical Abstract — KCNK1 binds to and activates LDHA to accelerate cellular glycolysis and lactate production, which enhances the level of histone lactylation modification of a series of downstream targets LDHA, ZWINT, ECT2, ANLN, and EZR, and promotes genes transcription and reduces stiffness and adhesion of breast cancer cells, all these ultimately lead to the proliferation and metastasis of breast cancer cells. Notably, KCNK1-activated LDHA serves as a positive feedback to sustain this vicious loop. (TIF) [file pbio.3002666.s001.tif]

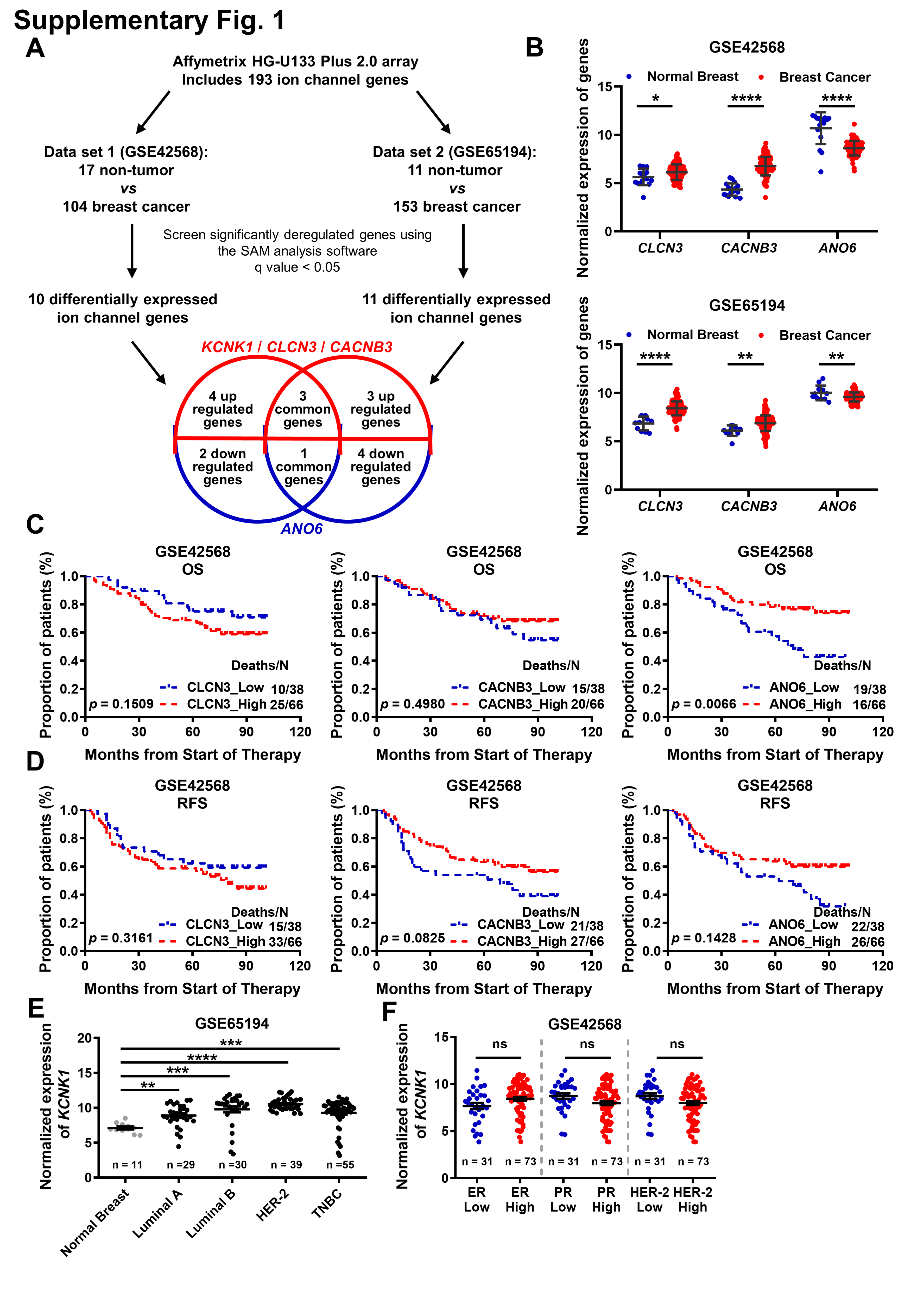

Supplement: S1 Fig — (A) Schematic workflow for identifying and validating the differentially expressed ion channels in 2 breast cancer datasets. (B) The expression of CLCN3, CACNB3, and ANO6 was analyzed in normal breast tissues and breast cancer tissues based on 2 datasets GSE42568 and GSE65194. (C, D) Kaplan–Meier analysis showed the effect CLCN3, CACNB3, and ANO6 expression on the overall survival (C) and relapse-free survival (D) of breast cancer patients in GSE42568. (E) The expression of KCNK1 was analyzed in different breast cancer subtypes according to dataset GSE65194. (F) The expression of KCNK1 was analyzed in breast cancer tissues based on dataset GSE42568. And p-values were calculated by unpaired two-sided t test in E and F. Source data are provided as S1 Data. (TIF) [file pbio.3002666.s004.tif]

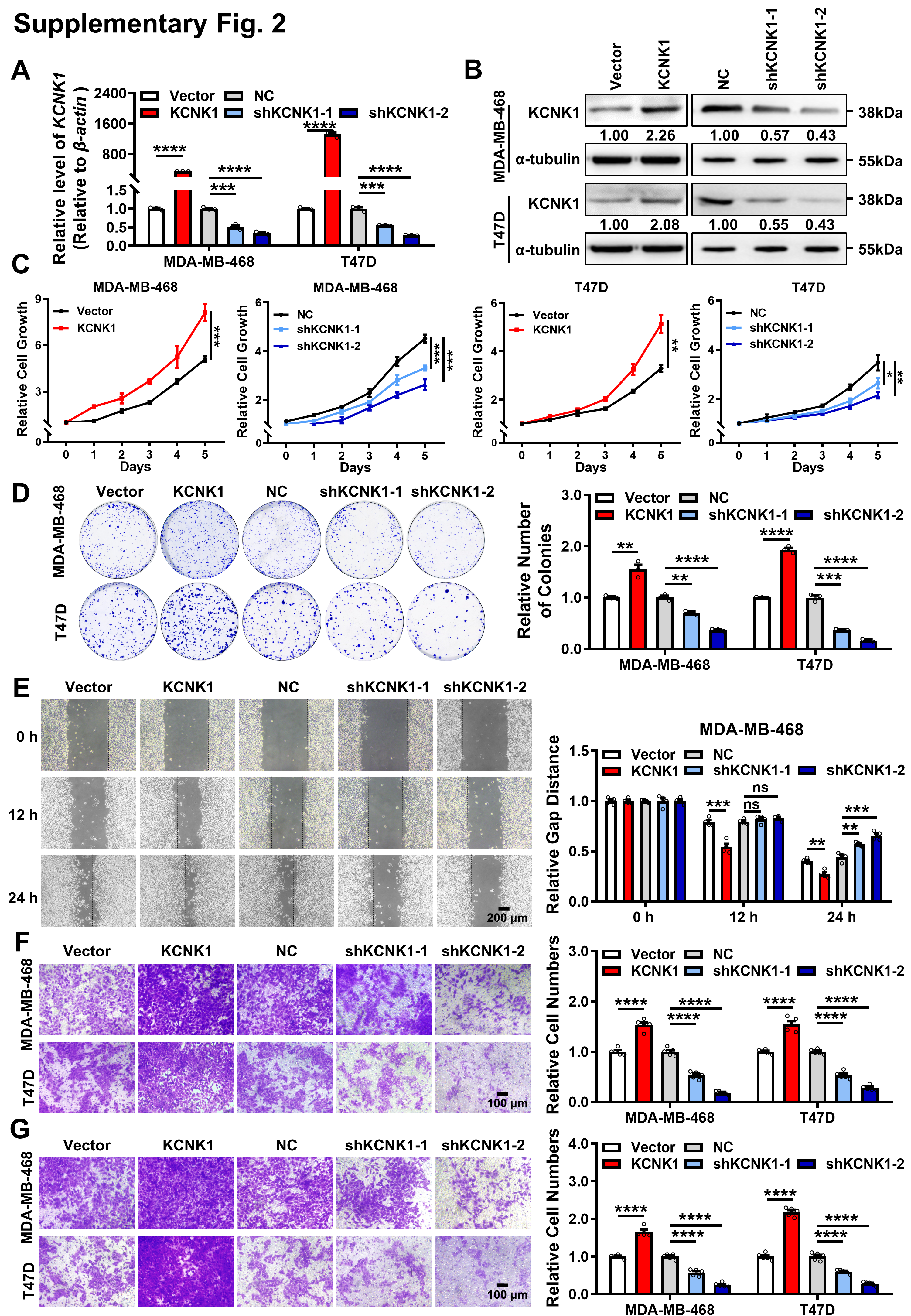

Supplement: S2 Fig — (A, B) The overexpression and knockdown efficiencies of KCNK1 were detected by qRT-PCR; (A) and western blotting assays (B) in MDA-MB-468 and T47D cells. (C–G) MTT assays (C), clone formation assays (D), wound healing (E), Transwell experiments without (F) or with Matrigel (G) were performed after KCNK1 overexpression or shRNAs knockdown in MDA-MB-468 and T47D cells. All experiments were performed in at least triplicate samples. Data were presented as mean ± SD, unpaired two-sided t tests were used to analyze the data. Source data are provided as S1 Data. (TIF) [file pbio.3002666.s005.tif]

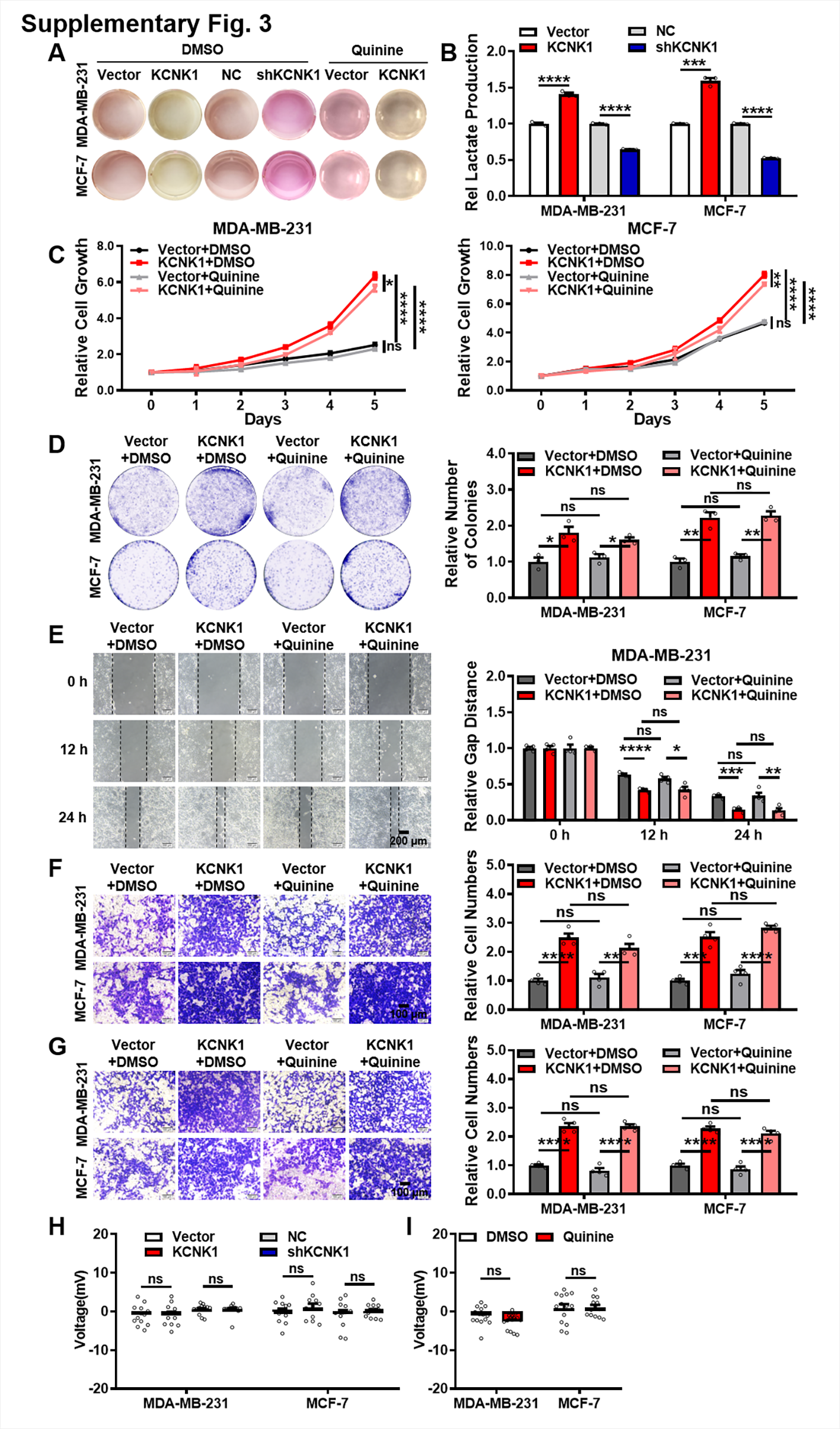

Supplement: S3 Fig — (A) Images of the medium color change the overexpression/knockdown of KCNK1 or treatment with Quinine. (B) The lactate production in breast cancer cells was detected after overexpression or knockdown of KCNK1. (C, D) The proliferation ability of MDA-MB-231 and MCF-7 cells were detected by MTT assays (C) and clone formation assays (D) after altering KCNK1 expression or treatment with Quinine. (E) Wound healing experiments were performed to detect the migration ability of MDA-MB-231 cells after altering KCNK1 expression or treatment with Quinine. (F) Transwell experiments without Matrigel were performed to detect the migration ability of MDA-MB-231 and MCF-7 cells after altering KCNK1 expression or treatment with Quinine. (G) Transwell experiments with Matrigel were performed to detect the invasion ability of MDA-MB-231 and MCF-7 cells after altering KCNK1 expression or treatment with Quinine. (H) Resting potential of breast cancer cells after overexpression or knockdown KCNK1 was measured. (I) Resting potential of breast cancer cells before and after quinine treatment was measured. All experiments were performed in at least triplicate samples. Data were presented as mean ± SD, unpaired two-sided t tests were used to analyze the data. Source data are provided as S1 Data. (TIF) [file pbio.3002666.s006.tif]

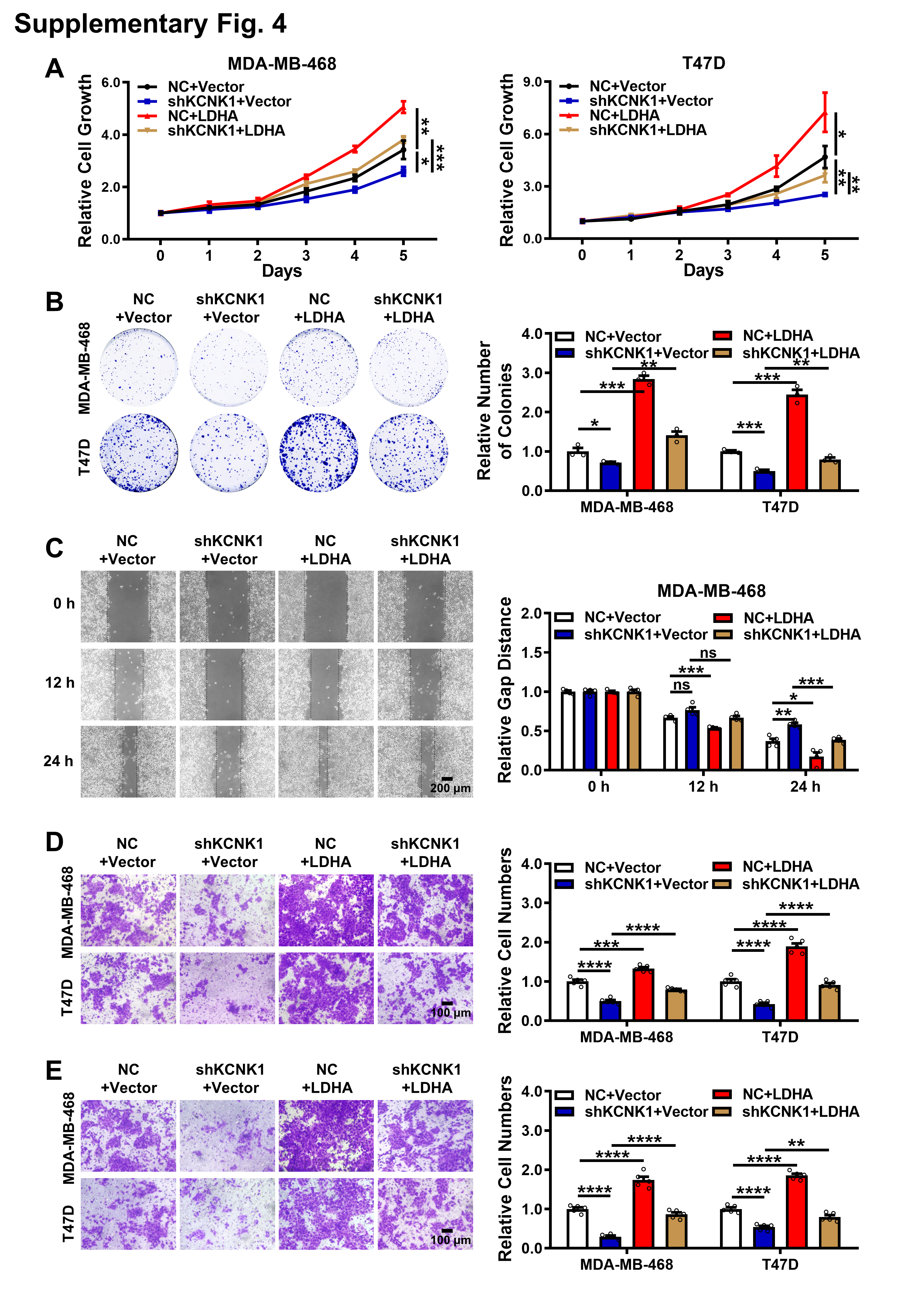

Supplement: S4 Fig — (A–E) MTT assays (A), clone formation assays (B), wound healing (C), Transwell experiments without (D) or with Matrigel (E) were performed after KCNK1 knockdown, LDHA overexpression, or co-transfection of shKCNK1 and LDHA overexpression vectors in MDA-MB-468 and T47D cells. All experiments were performed in at least triplicate samples. Data were presented as mean ± SD, unpaired two-sided t tests were used to analyze the data. Source data are provided as S1 Data. (TIF) [file pbio.3002666.s007.tif]

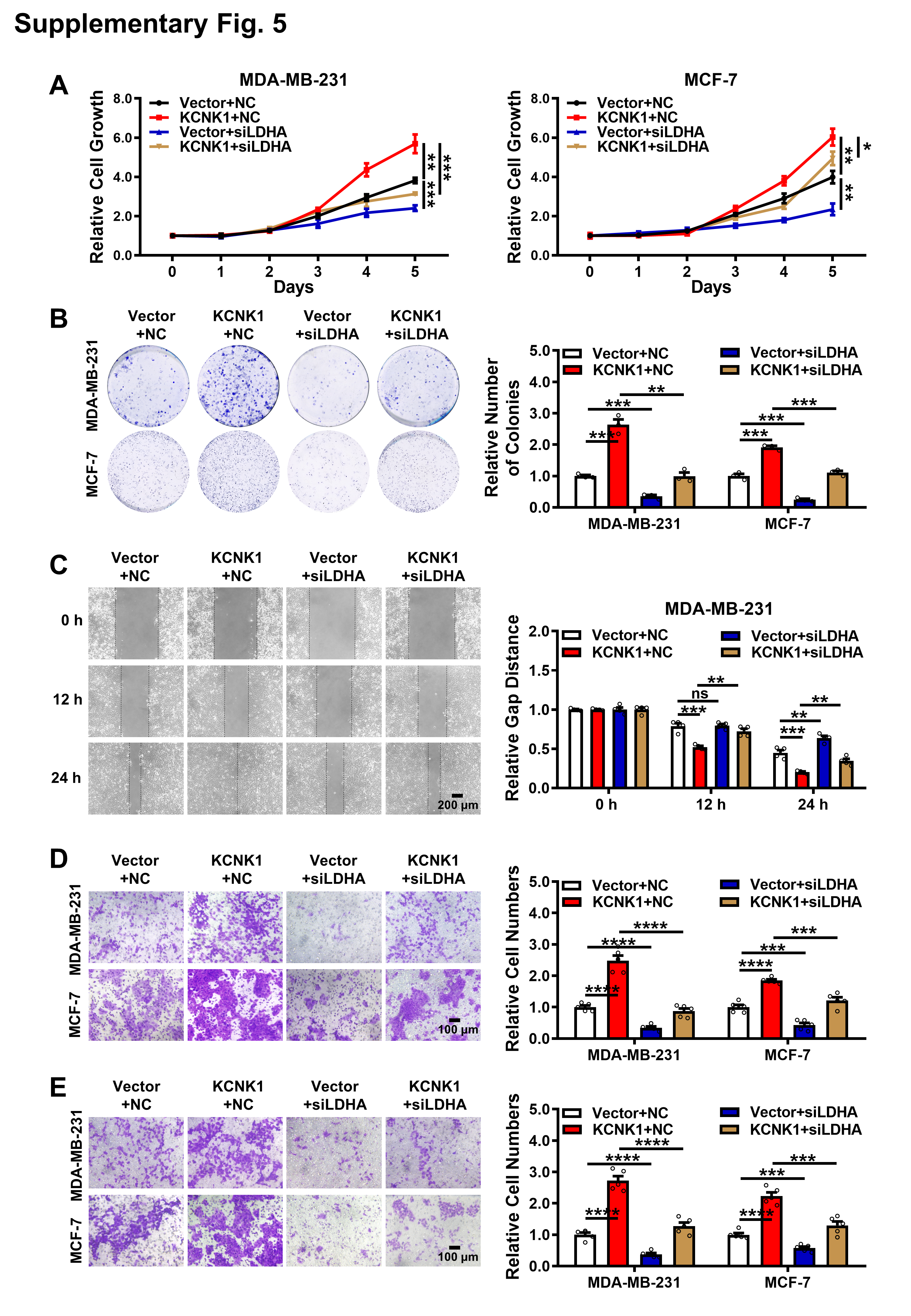

Supplement: S5 Fig — (A–E) MTT assays (A), clone formation assays (B), wound healing (C), Transwell experiments without (D) or with Matrigel (E) were performed after KCNK1 overexpression, LDHA knockdown, or co-transfection of siLDHA and KCNK1 overexpression vectors in MDA-MB-231 and MCF-7 cells. All experiments were performed in at least triplicate samples. Data were presented as mean ± SD, unpaired two-sided t tests were used to analyze the data. Source data are provided as S1 Data. (TIF) [file pbio.3002666.s008.tif]

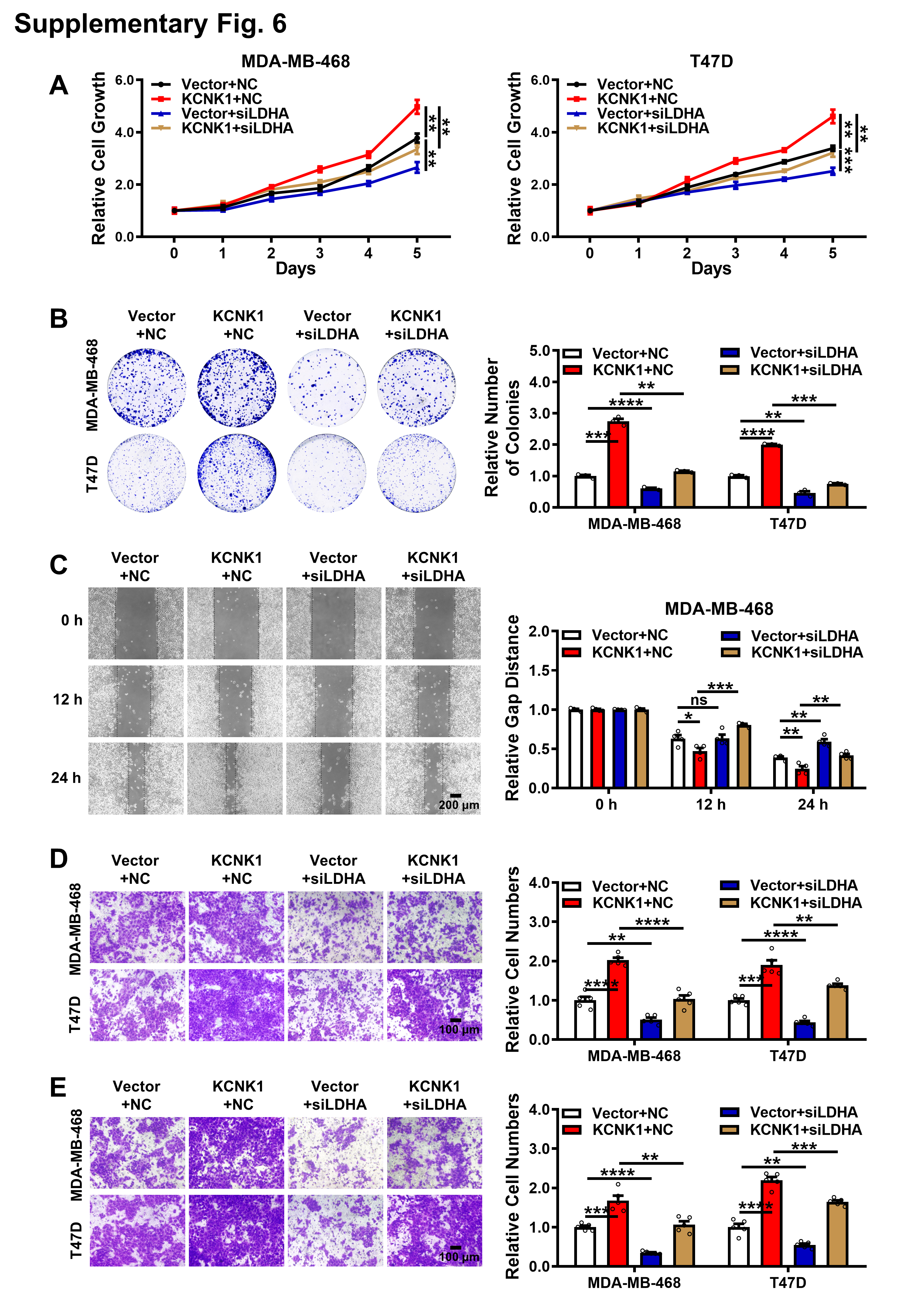

Supplement: S6 Fig — (A–E) MTT assays (A), clone formation assays (B), wound healing (C), Transwell experiments without (D) or with Matrigel (E) were performed after KCNK1 overexpression, LDHA knockdown, or co-transfection of siLDHA and KCNK1 overexpression vectors in MDA-MB-468 and T47D cells. All experiments were performed in at least triplicate samples. Data were presented as mean ± SD, unpaired two-sided t tests were used to analyze the data. Source data are provided as S1 Data. (TIF) [file pbio.3002666.s009.tif]

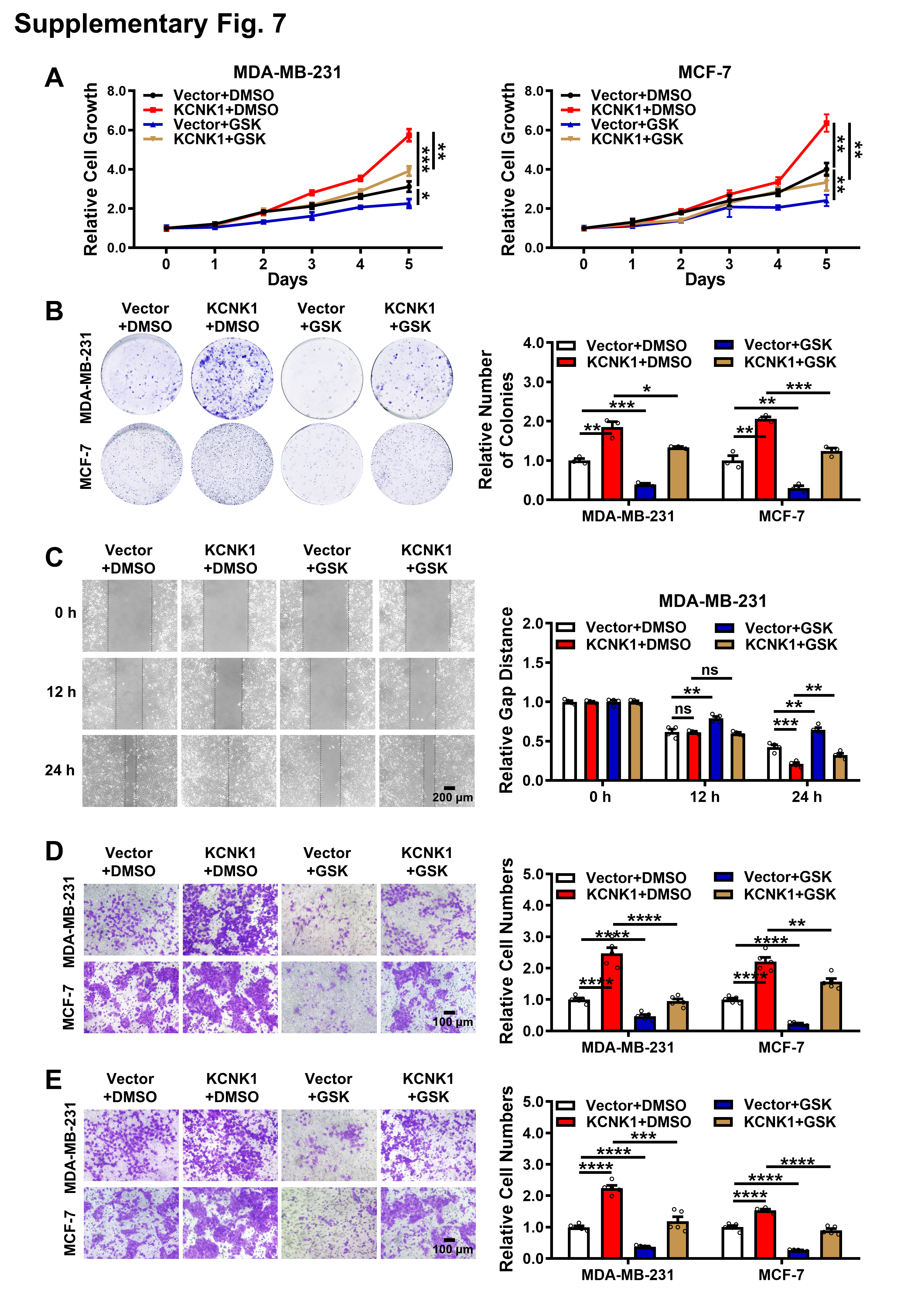

Supplement: S7 Fig — (A–E) MTT assays (A), clone formation assays (B), wound healing (C), Transwell experiments without (D) or with Matrigel (E) were performed after KCNK1 overexpression, treatment with GSK, or treatment with GSK in parallel with KCNK1 overexpression in MDA-MB-231 and MCF-7 cells. All experiments were performed in at least triplicate samples. Data were presented as mean ± SD, unpaired two-sided t tests were used to analyze the data. Source data are provided as S1 Data. (TIF) [file pbio.3002666.s010.tif]

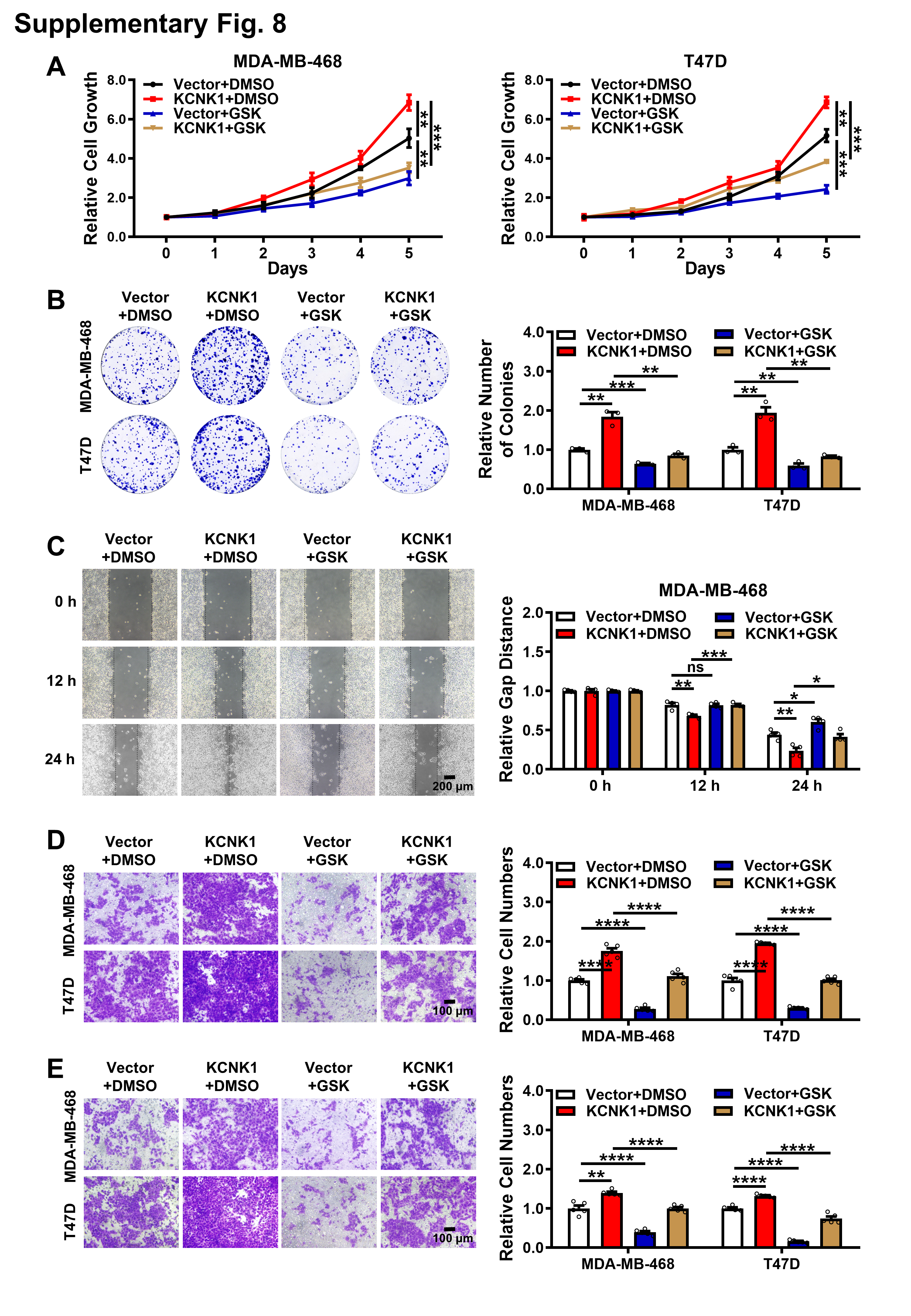

Supplement: S8 Fig — (A–E) MTT assays (A), clone formation assays (B), wound healing (C), Transwell experiments without (D) or with Matrigel (E) were performed after KCNK1 overexpression, treatment with GSK, or treatment with GSK in parallel with KCNK1 overexpression in MDA-MB-468 and T47D cells. All experiments were performed in at least triplicate samples. Data were presented as mean ± SD, unpaired two-sided t tests were used to analyze the data. Source data are provided as S1 Data. (TIF) [file pbio.3002666.s011.tif]

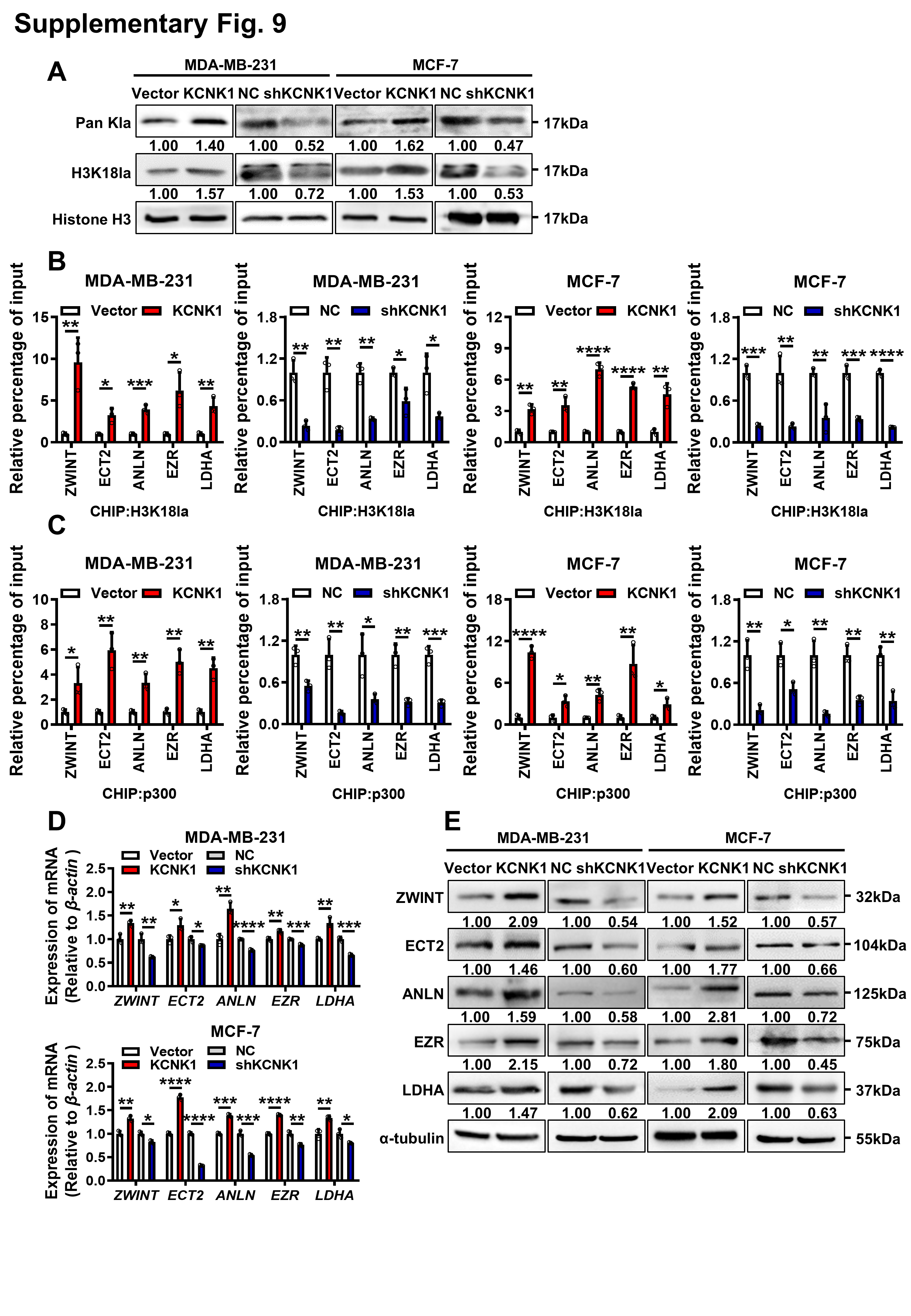

Supplement: S9 Fig — (A) The pan histone and H3K18 site lactylation levels were detected in breast cancer cells after overexpression or knockdown of KCNK1 by western blotting assays. (B) ChIP-qPCR assay showed the H3K18la status in ZWINT, ECT2, ANLN, EZR, and LDHA genomic regions in MDA-MB-231 and MCF-7 cells after overexpression or knockdown of KCNK1. (C) ChIP-qPCR assay showed the p300 enrichment in ZWINT, ECT2, ANLN, EZR, and LDHA genomic regions in MDA-MB-231 and MCF-7 cells after overexpression or knockdown of KCNK1. (D) The expression of ZWINT, ECT2, ANLN, EZR, and LDHA was examined in MDA-MB-231 and MCF-7 cells using qRT-PCR after overexpression or knockdown of KCNK1. (E) The expression of KCNK1 downstream targets was examined in breast cancer cells using western blotting after KCNK1 overexpression or knockdown. All experiments were performed in at least triplicate samples. Data were presented as mean ± SD, unpaired two-sided t tests were used to analyze the data. Source data are provided as S1 Data. (TIF) [file pbio.3002666.s012.tif]

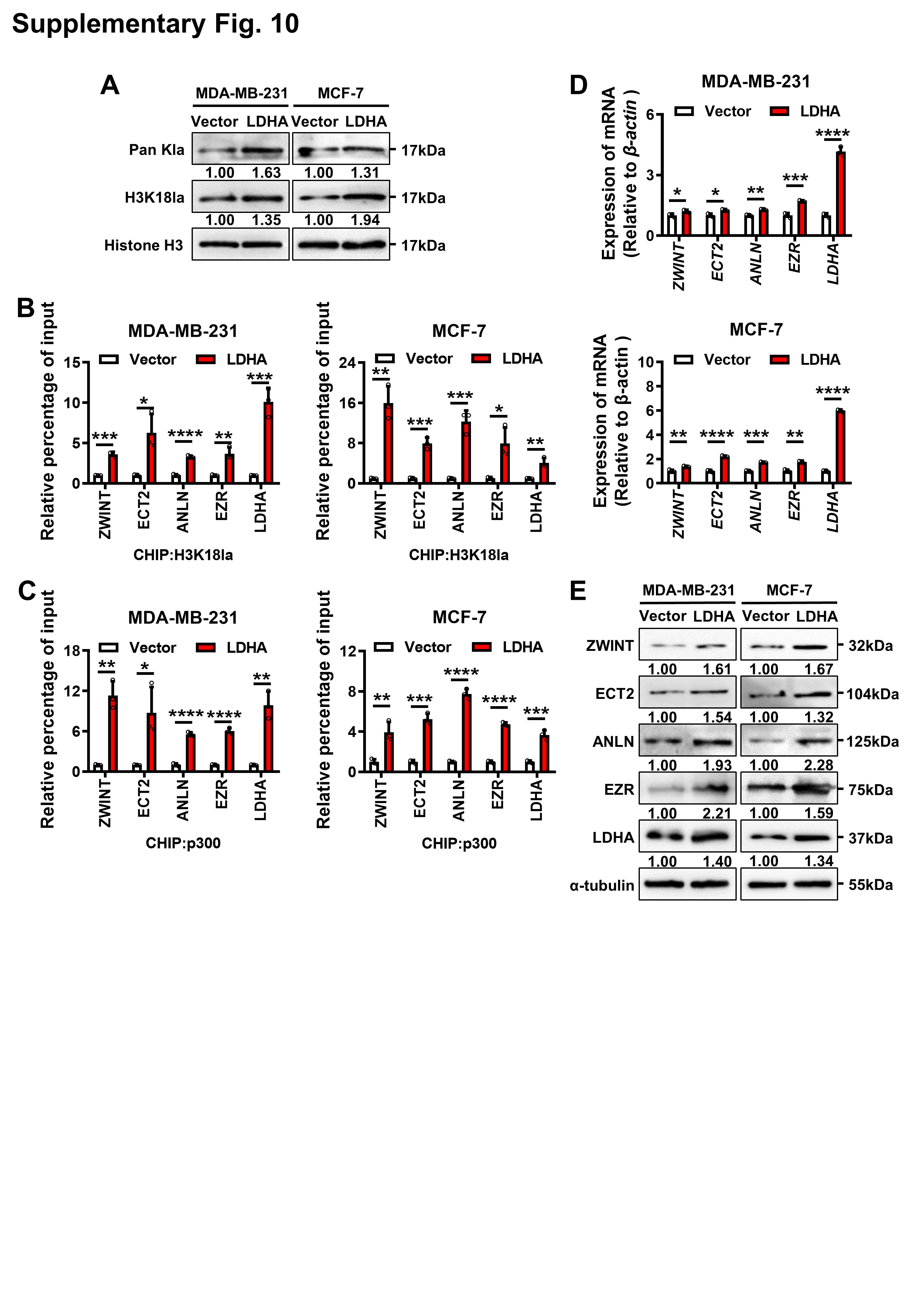

Supplement: S10 Fig — (A) Western blotting was used to detect pan histone and H3K18 site lactylation levels in LDHA overexpressed breast cancer cells. (B) ChIP-qPCR assay showed the H3K18la status in ZWINT, ECT2, ANLN, EZR, and LDHA genomic regions in MDA-MB-231 and MCF-7 cells after overexpression of LDHA. (C) ChIP-qPCR assay showed the p300 enrichment in ZWINT, ECT2, ANLN, EZR, and LDHA genomic regions in MDA-MB-231 and MCF-7 cells after overexpression of LDHA. (D) Expression of ZWINT, ECT2, ANLN, EZR, and LDHA was detected in MDA-MB-231 and MCF-7 cells after overexpression of LDHA using qRT-PCR. (E) Expression of ZWINT, ECT2, ANLN, EZR, and LDHA was detected in breast cancer cells after overexpression of LDHA using western blotting. All experiments were performed in at least triplicate samples. Data were presented as mean ± SD, unpaired two-sided t tests were used to analyze the data. Source data are provided as S1 Data. (TIF) [file pbio.3002666.s013.tif]

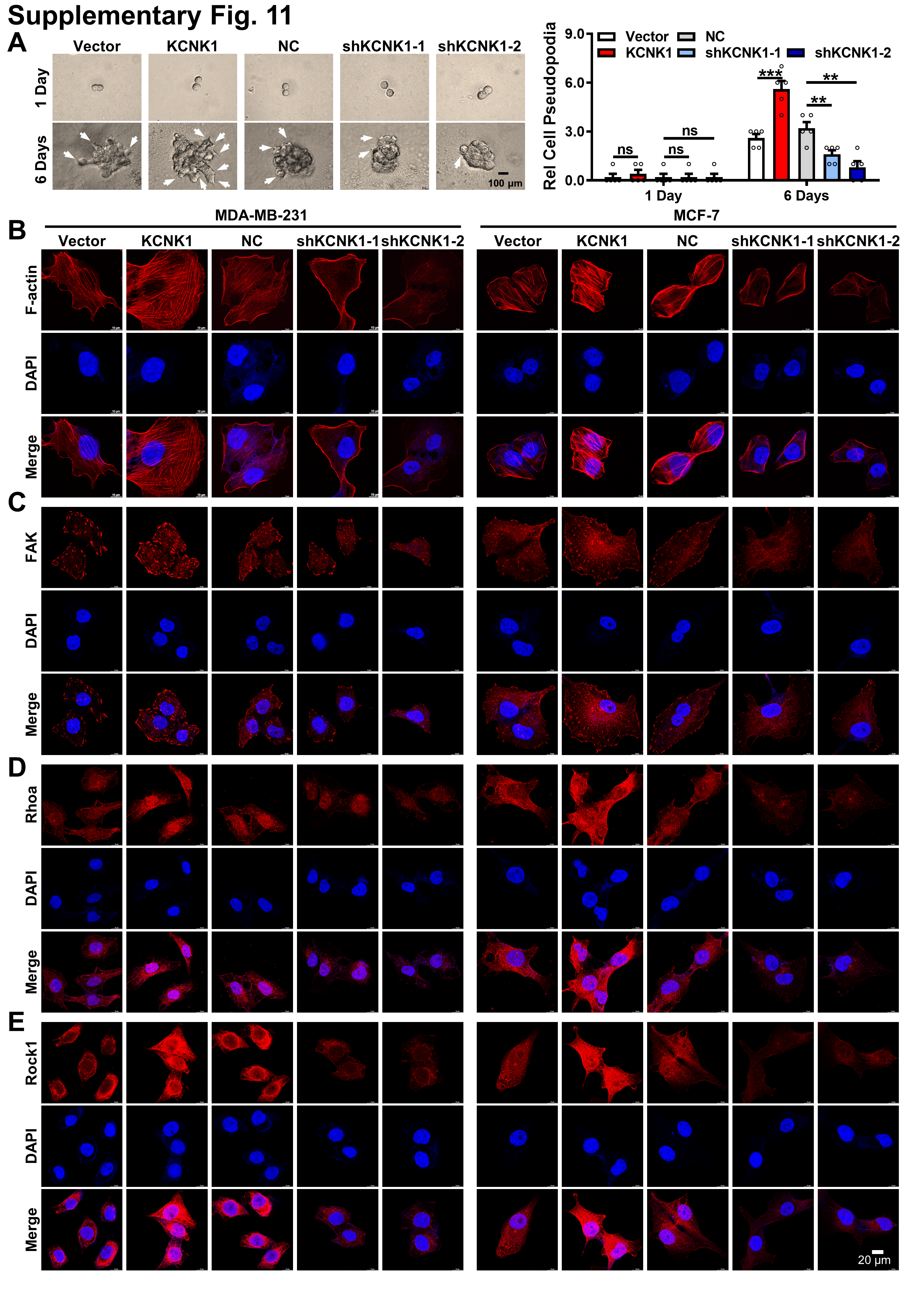

Supplement: S11 Fig — (A) A 3D culture model formed in Matrigel after overexpression or knockdown of KCNK1 in MDA-MB-231 cells. White arrows indicate scattered protrusions formed on the spheroid surfaces. And p-values were calculated by unpaired two-sided t test. (B–E) The F-actin, FAK, RhoA, and Rock1 protein was detected by IF after KCNK1 overexpression or knockdown. DAPI: blue; F-actin, FAK, RhoA, and Rock1: red. (TIF) [file pbio.3002666.s014.tif]

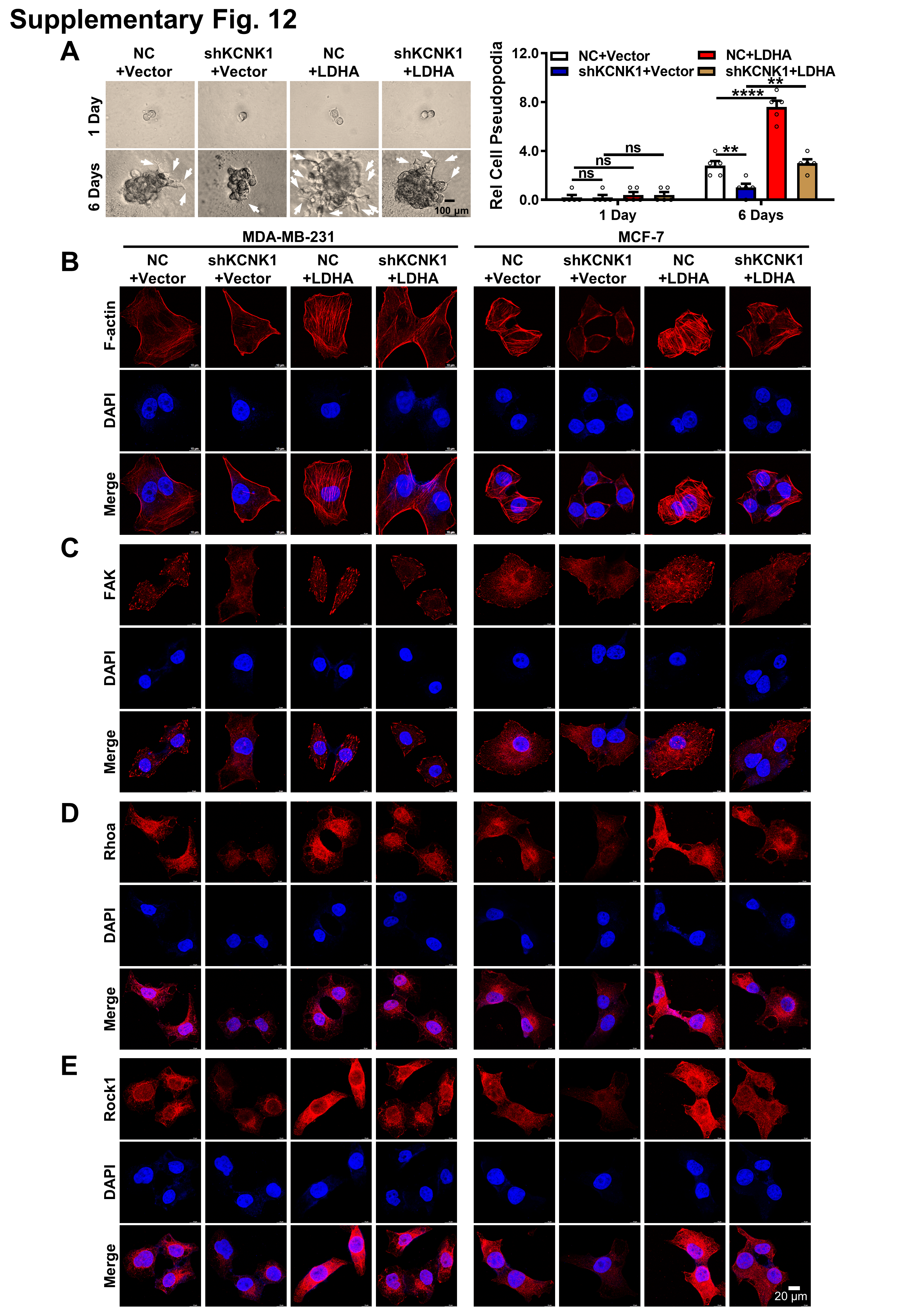

Supplement: S12 Fig — (A) A 3D culture model formed in Matrigel after KCNK1 knockdown, LDHA overexpression, or co-transfection of shKCNK1 and LDHA overexpression vectors in MDA-MB-231 cells. White arrows indicate scattered protrusions formed on the spheroid surfaces. And p-values were calculated by unpaired two-sided t test. (B–E) The F-actin, FAK, RhoA, and Rock1 protein was detected by IF after KCNK1 knockdown, LDHA overexpression, or co-transfection of shKCNK1 and LDHA overexpression vectors. DAPI: blue; F-actin, FAK, RhoA, and Rock1: red. (TIF) [file pbio.3002666.s015.tif]

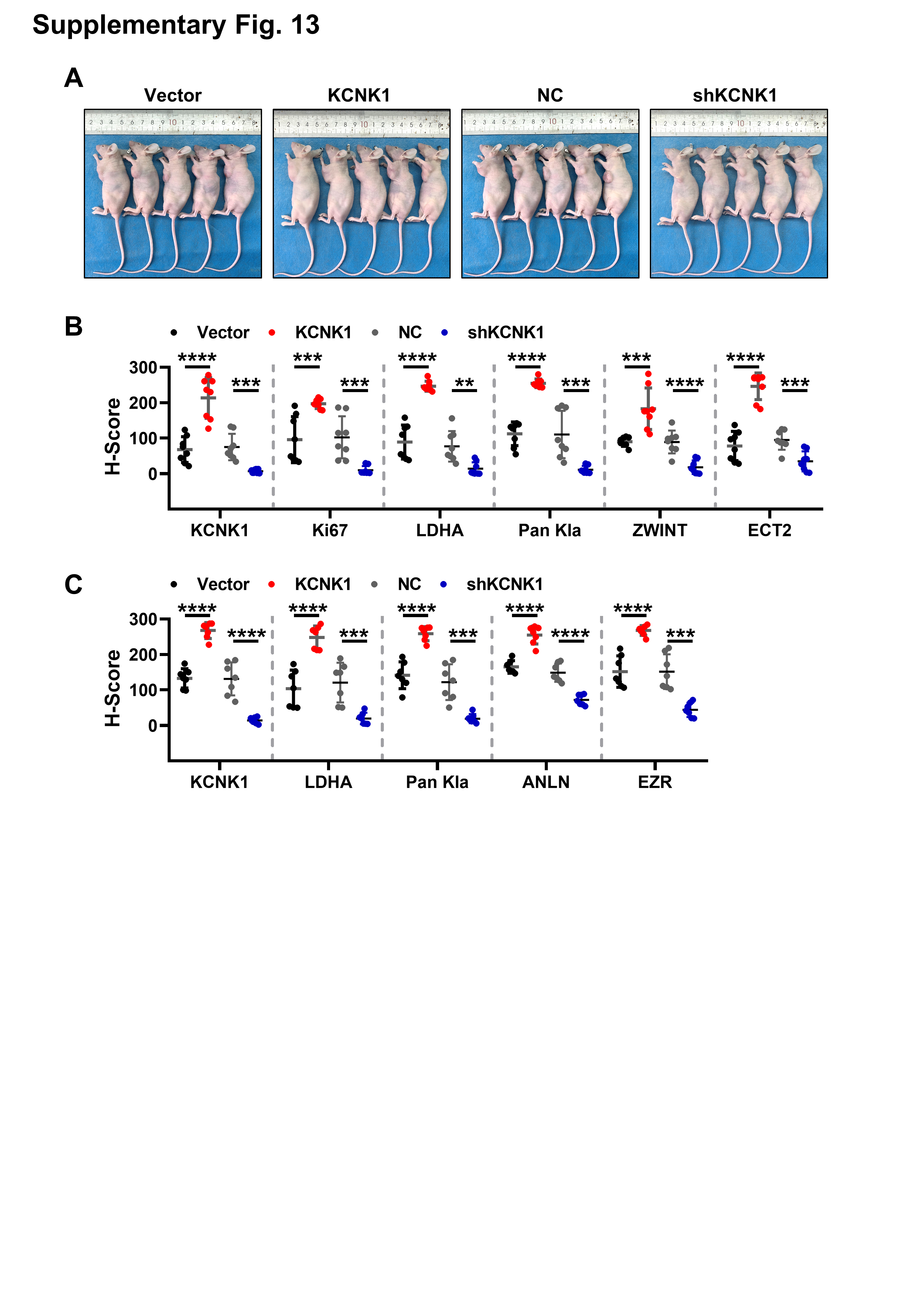

Supplement: S13 Fig — (A) Images of subcutaneous tumorigenic mice after injection with MDA-MB-231 cells transfected with the KCNK1 overexpression or shRNA vectors. (B) The statistical analysis of KCNK1, Ki67, LDHA, Pan Kla, ZWINT, and ECT2 expression in subcutaneous tumors tissues. (C) The statistical analysis of KCNK1, LDHA, Pan Kla, ANLN, and EZR expression in lung metastatic nodules. Data were presented as mean ± SD, unpaired two-sided t tests were used to analyze the data. Source data are provided as S1 Data. (TIF) [file pbio.3002666.s016.tif]
