# Supplementary material for: KCNK1 promotes proliferation and metastasis of breast cancer cells by activating lactate dehydrogenase A (LDHA) and up-regulating H3K18 lactylation
Source: PLoS Biol. 2024 Jun 21;22(6):e3002666. doi: 10.1371/journal.pbio.3002666 (PMC11192366; doi:10.1371/journal.pbio.3002666)

Figure 2b repeat 1

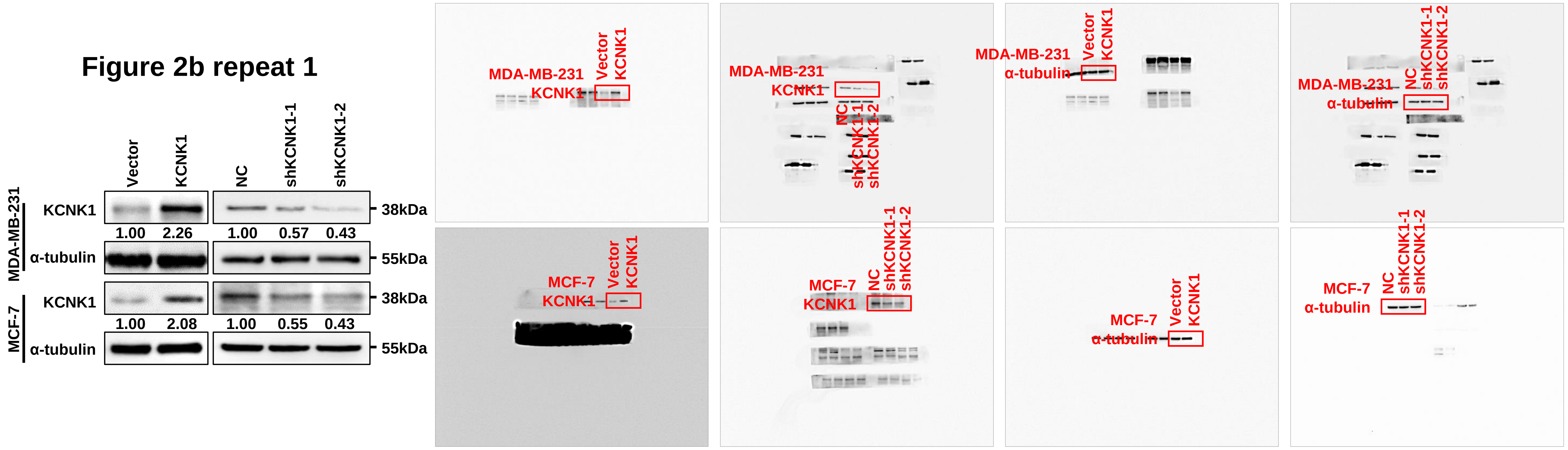

Figure 2b repeat 2

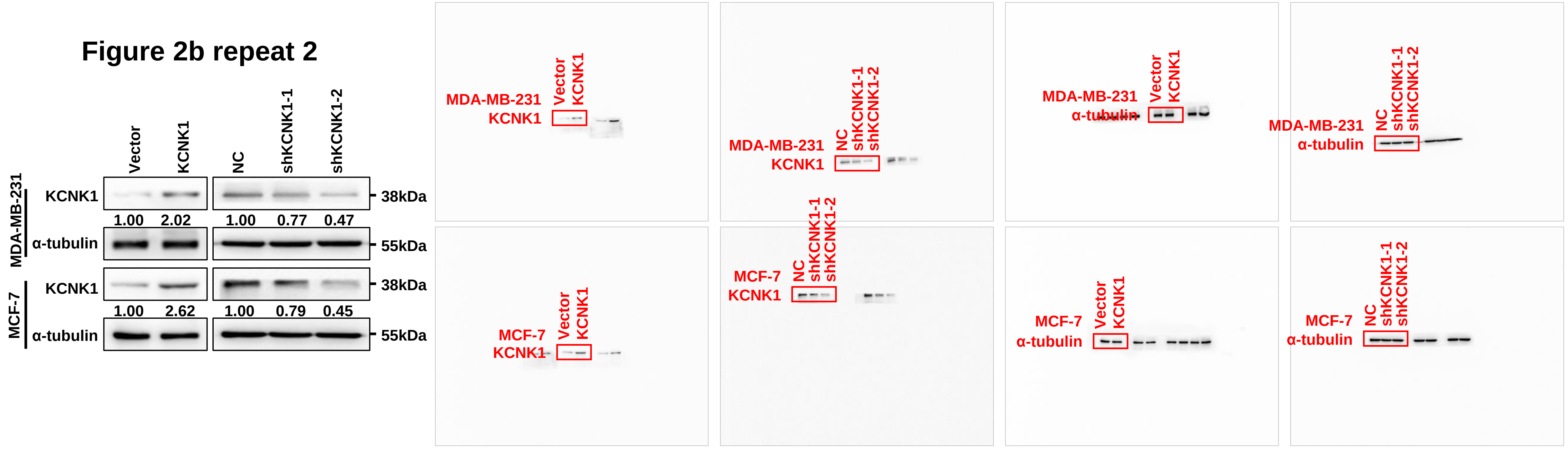

Figure 2b repeat 3

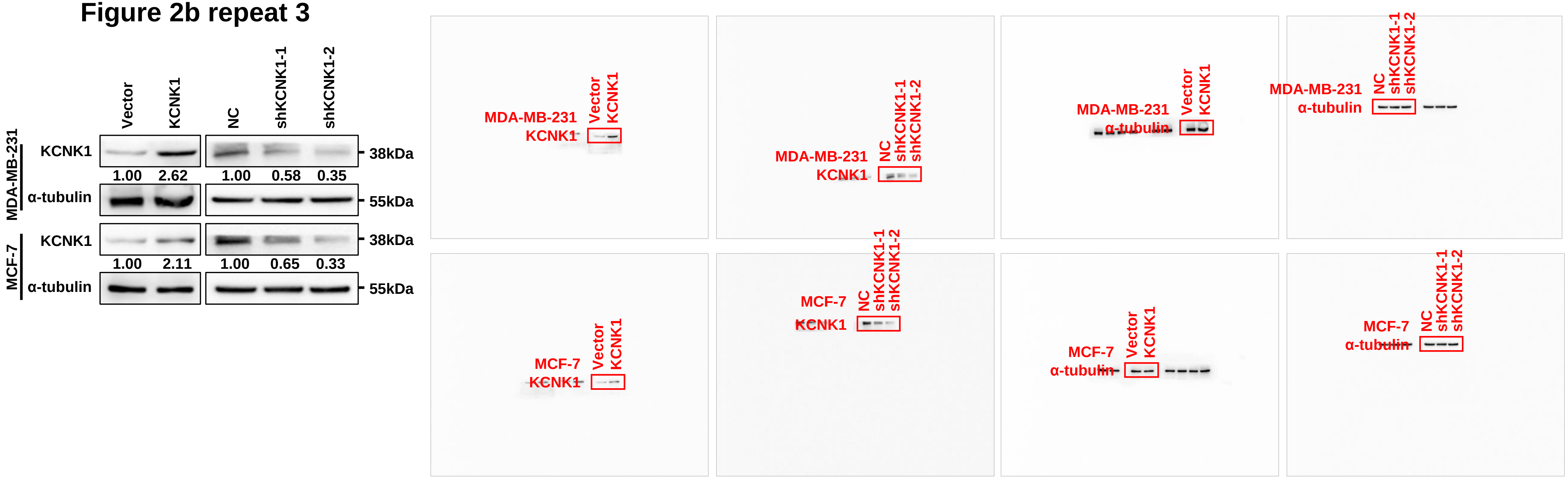

Figure 3c

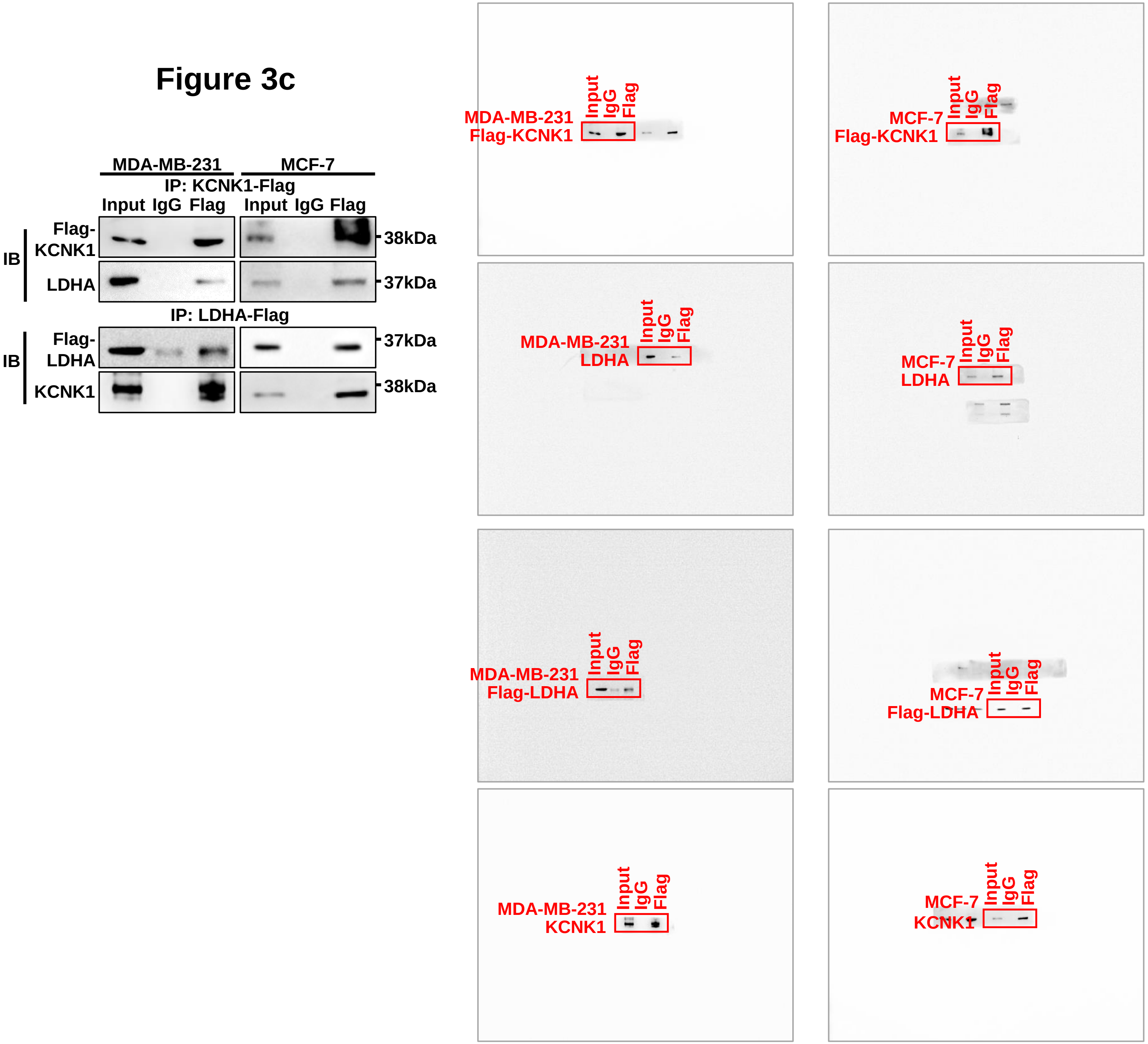
MDA-MB-231
MCF-7
IP: KCNK1-Flag
Input IgG Flag Input IgG Flag
IB
Flag-KCNK1
LDHA
38kDa
37kDa
IP: LDHA-Flag
IB
Flag-LDHA
KCNK1
37kDa
38kDa
MDA-MB-231 Flag-KCNK1
Input IgG Flag
MCF-7 Flag-KCNK1
MDA-MB-231 LDHA
MCF-7 LDHA
MDA-MB-231 Flag-LDHA
MCF-7 Flag-LDHA
MDA-MB-231 KCNK1
MCF-7 KCNK1

Figure 3e repeat 1

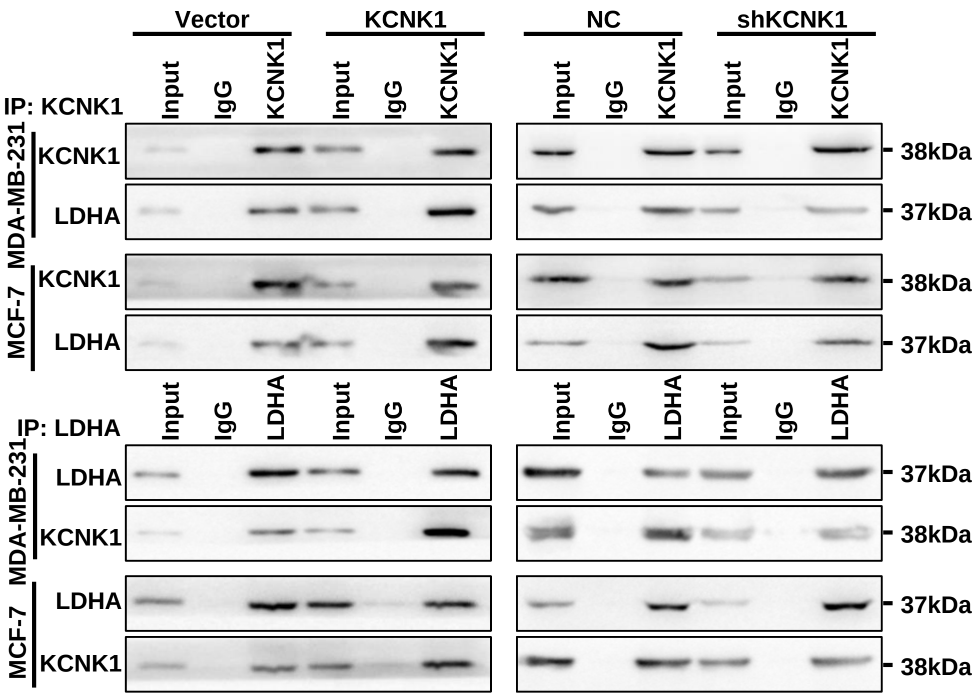

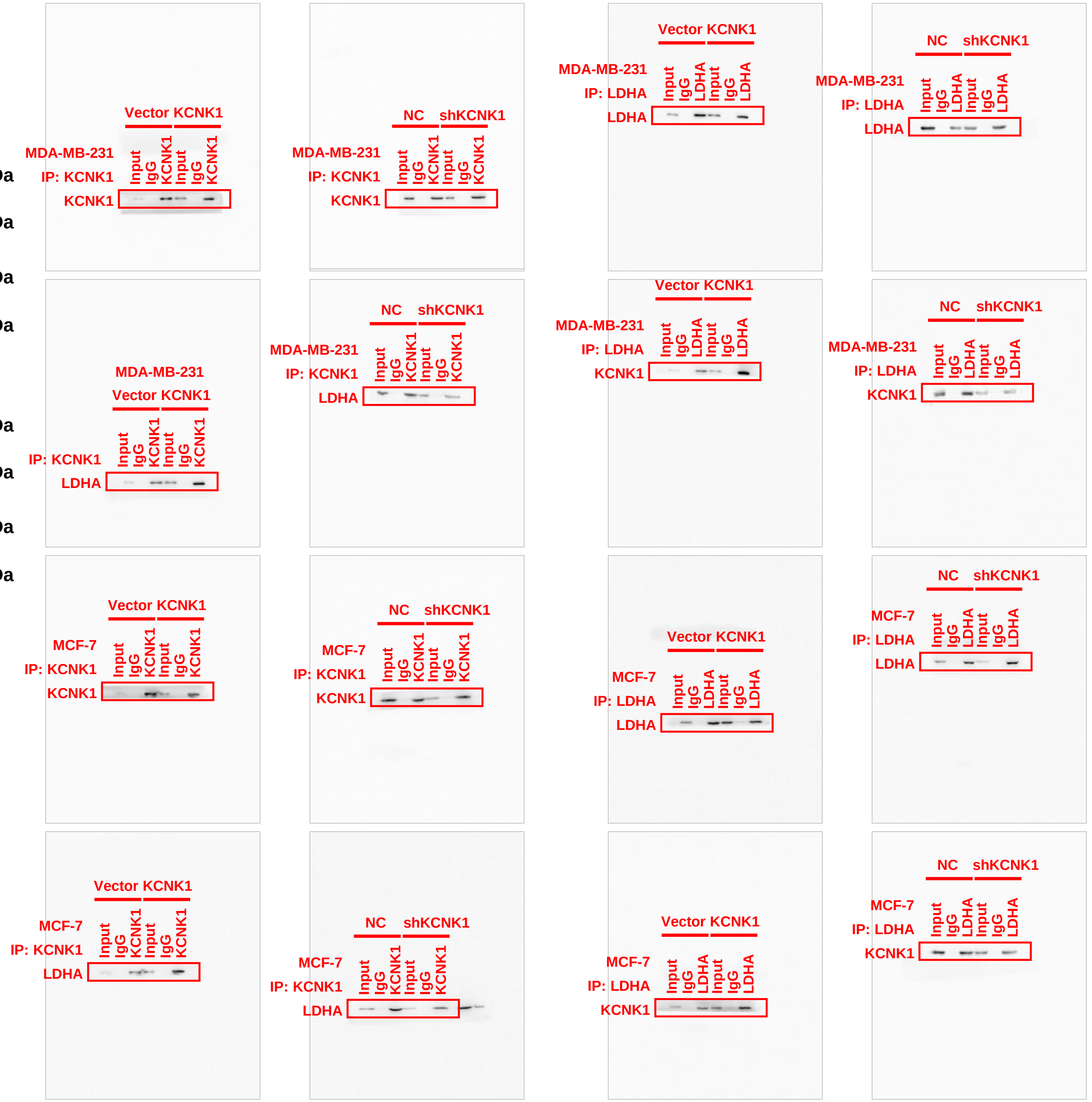

Figure 3e repeat 2

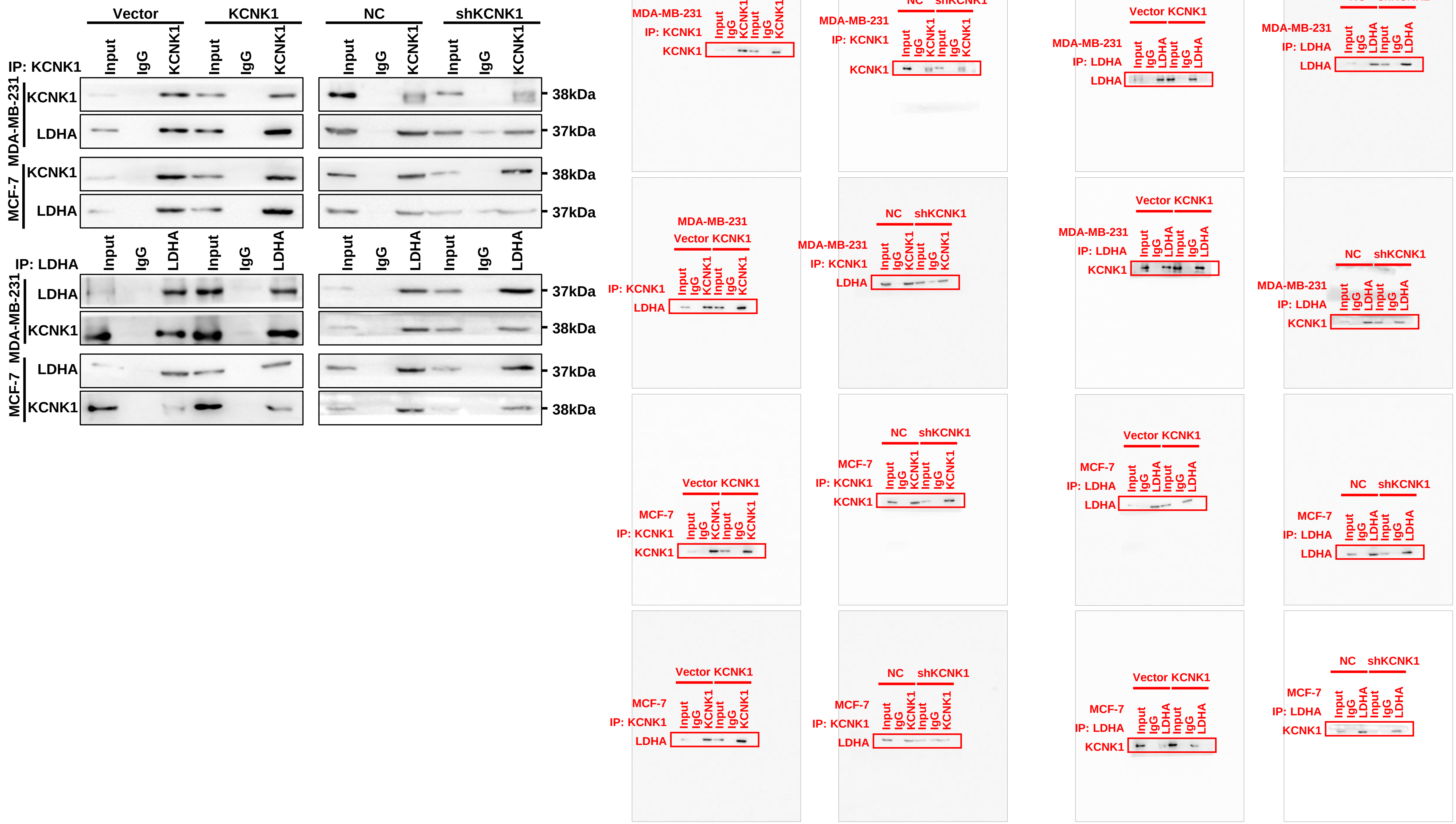

Figure 3e repeat 3

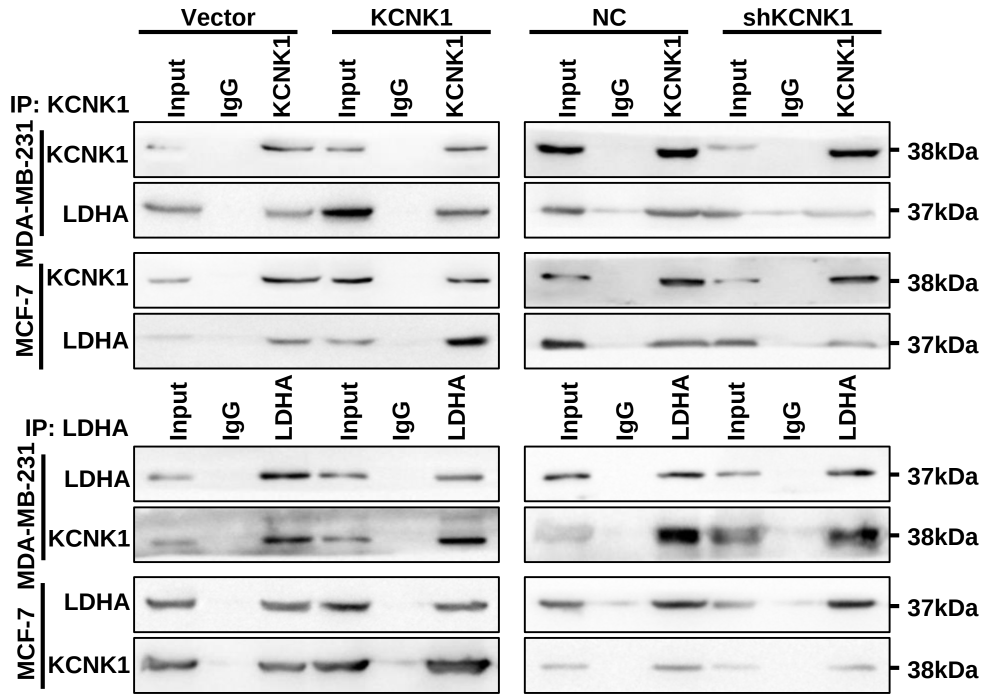

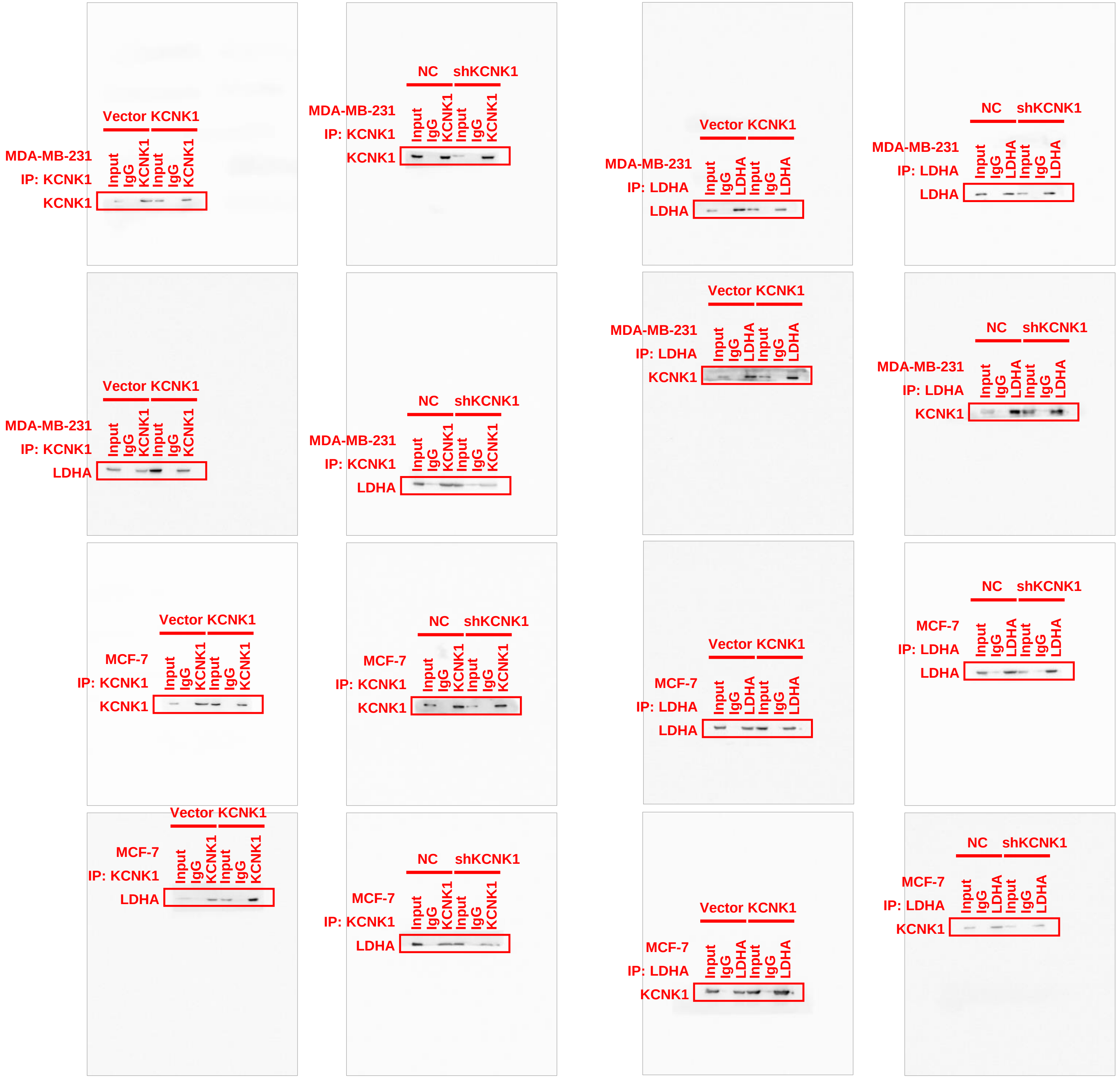

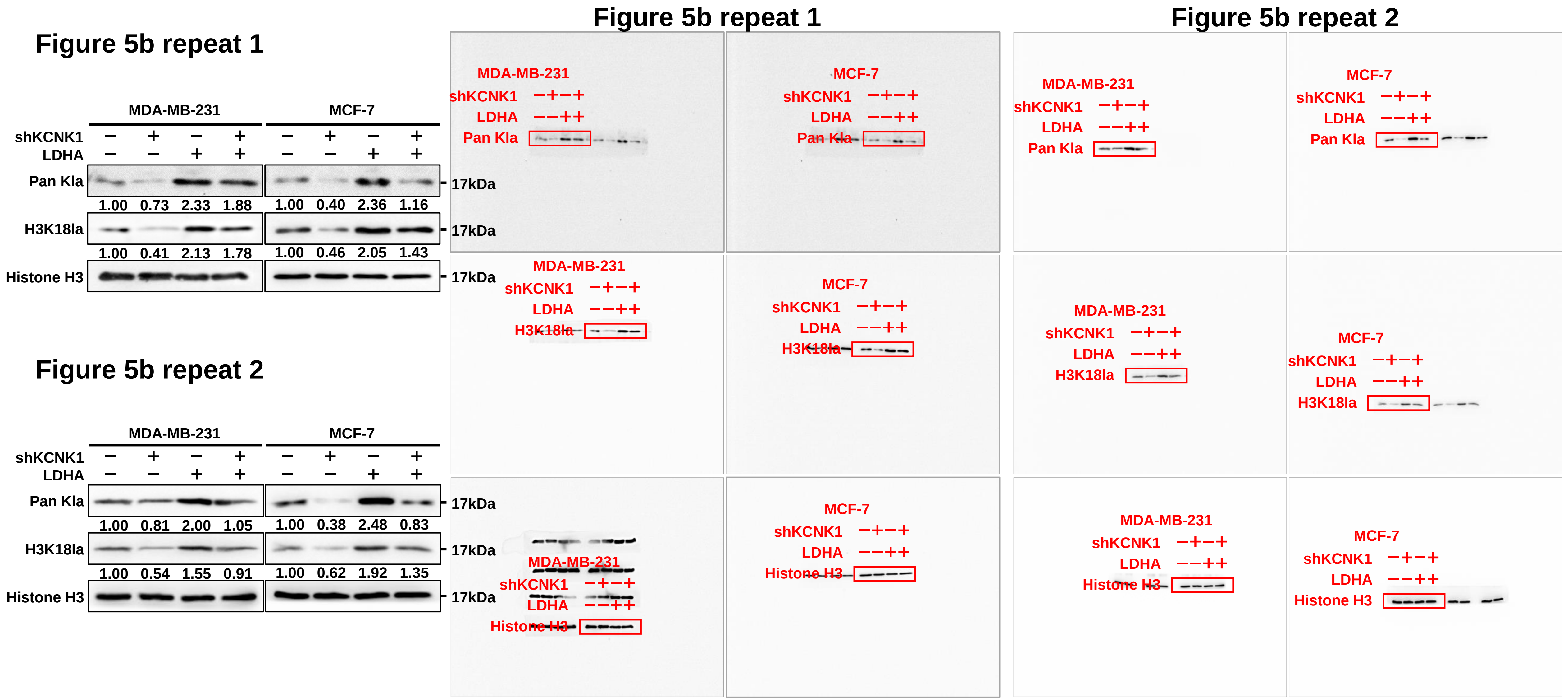
Figure 5b repeat 1
MDA-MB-231
MCF-7
shKCNK1 − + − + − + − +
LDHA − − + + − − + +
Pan Kla
1.00 0.73 2.33 1.88 1.00 0.40 2.36 1.16
H3K18la
1.00 0.41 2.13 1.78 1.00 0.46 2.05 1.43
Histone H3
17kDa
Figure 5b repeat 2
MDA-MB-231
MCF-7
shKCNK1 − + − + − + − +
LDHA − − + + − − + +
Pan Kla
1.00 0.81 2.00 1.05 1.00 0.38 2.48 0.83
H3K18la
1.00 0.54 1.55 0.91 1.00 0.62 1.92 1.35
Histone H3
17kDa
Figure 5b repeat 1
Figure 5b repeat 2
MDA-MB-231
MCF-7
shKCNK1 −+−+
LDHA −−++
Pan Kla
H3K18la
Histone H3

Figure 5b repeat 3

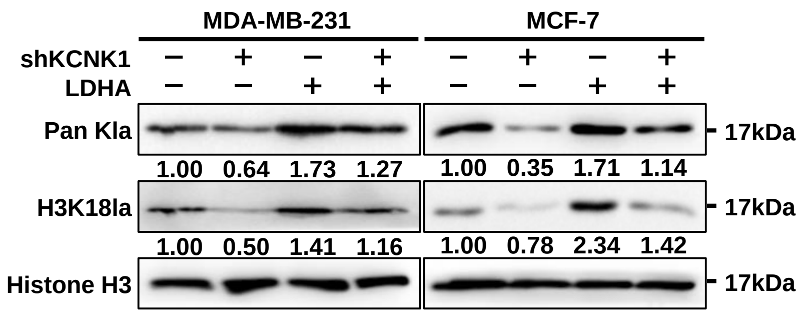

Figure 5b repeat 3

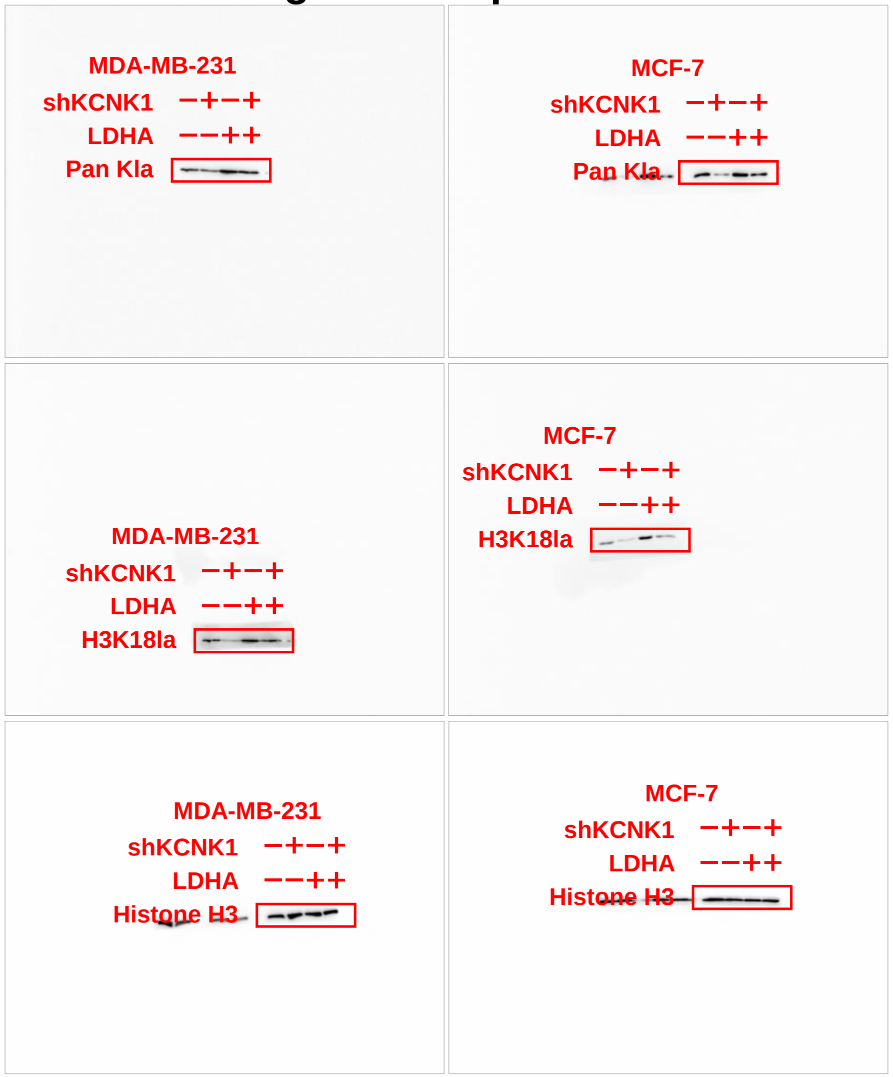

Figure 5f repeat 1

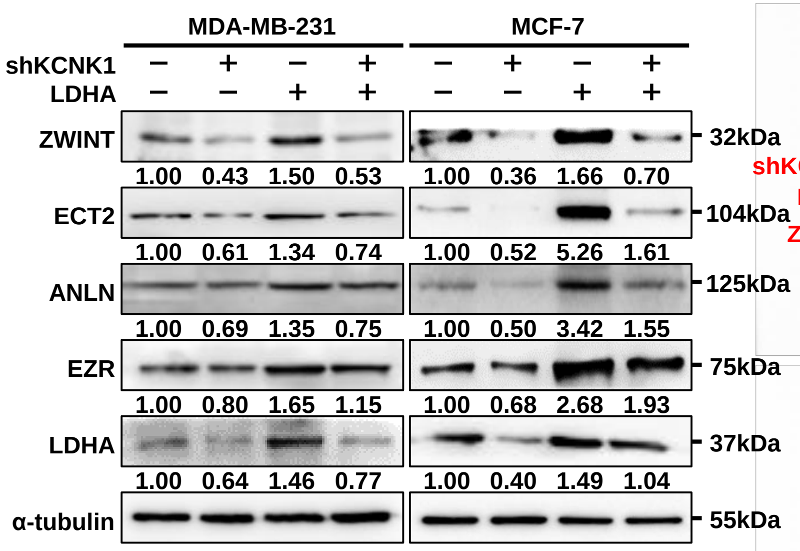

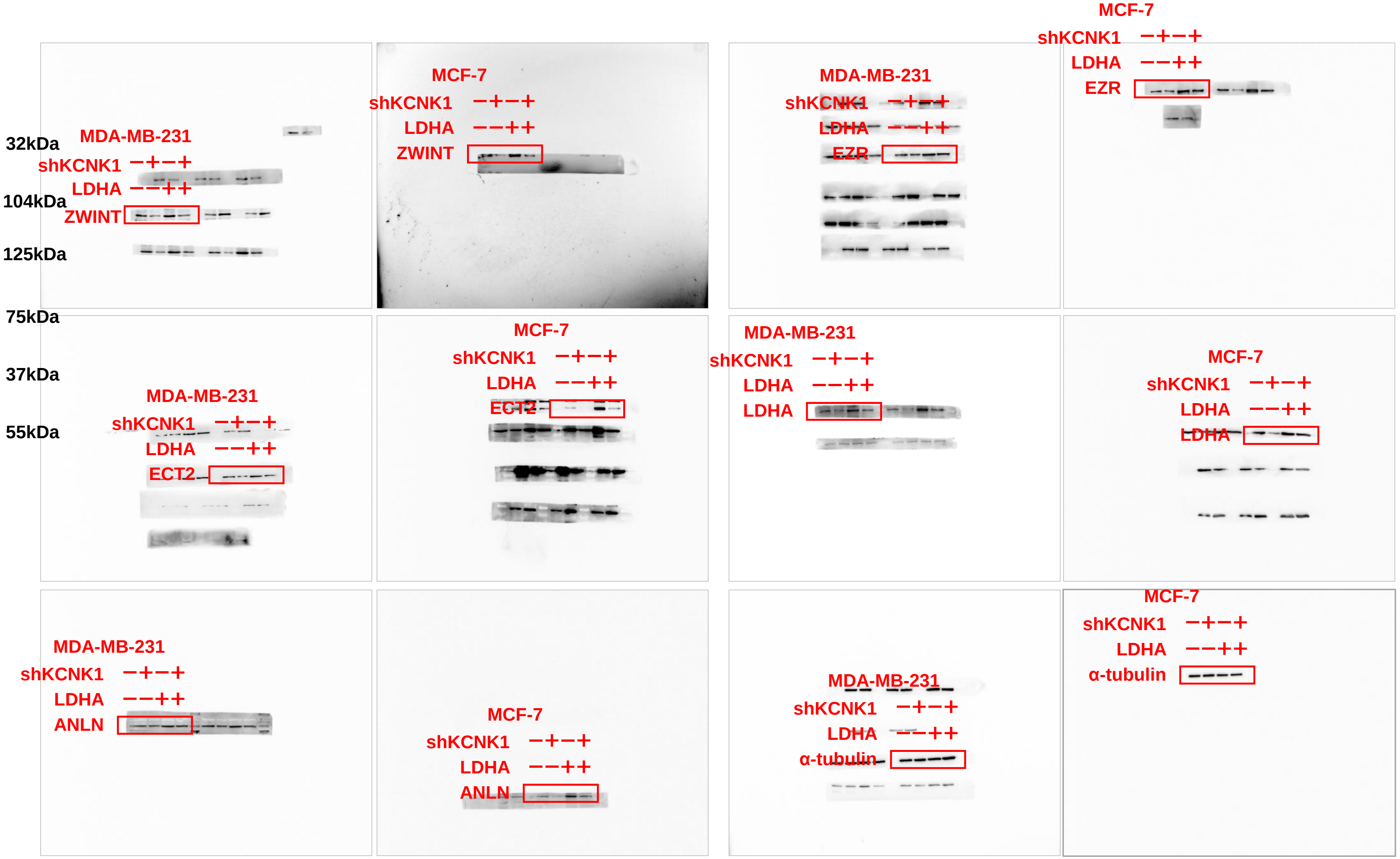

Figure 5f repeat 2

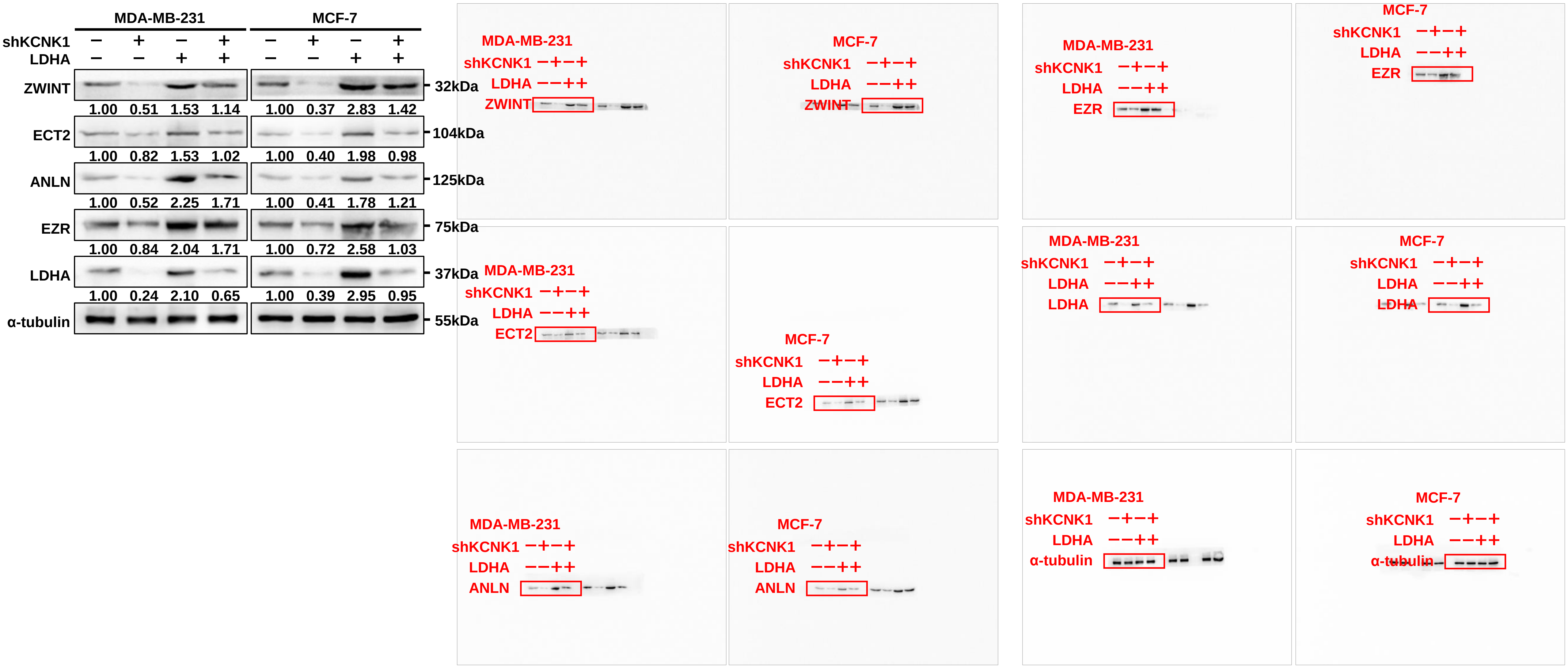

Figure 5f repeat 3

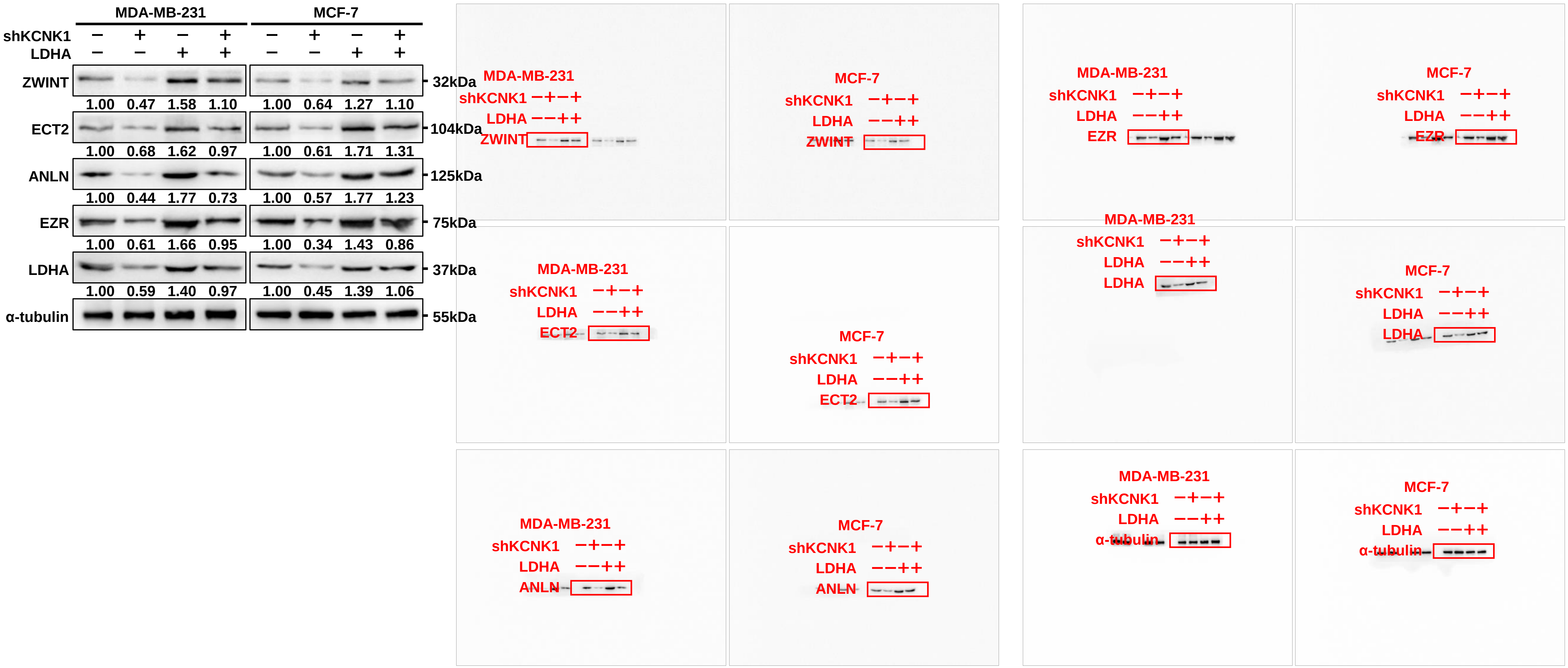

Supplementary Fig. S2b repeat 1

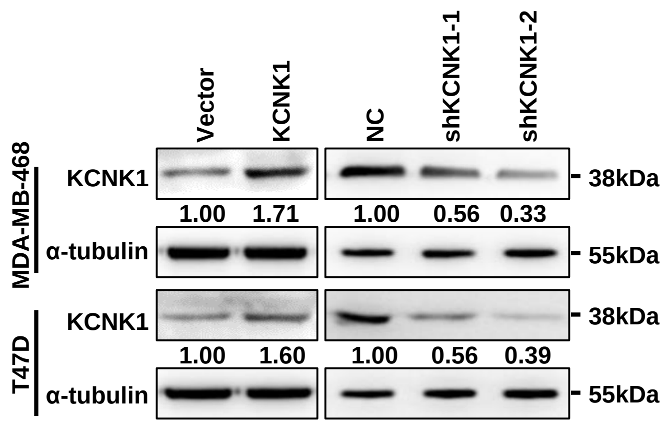

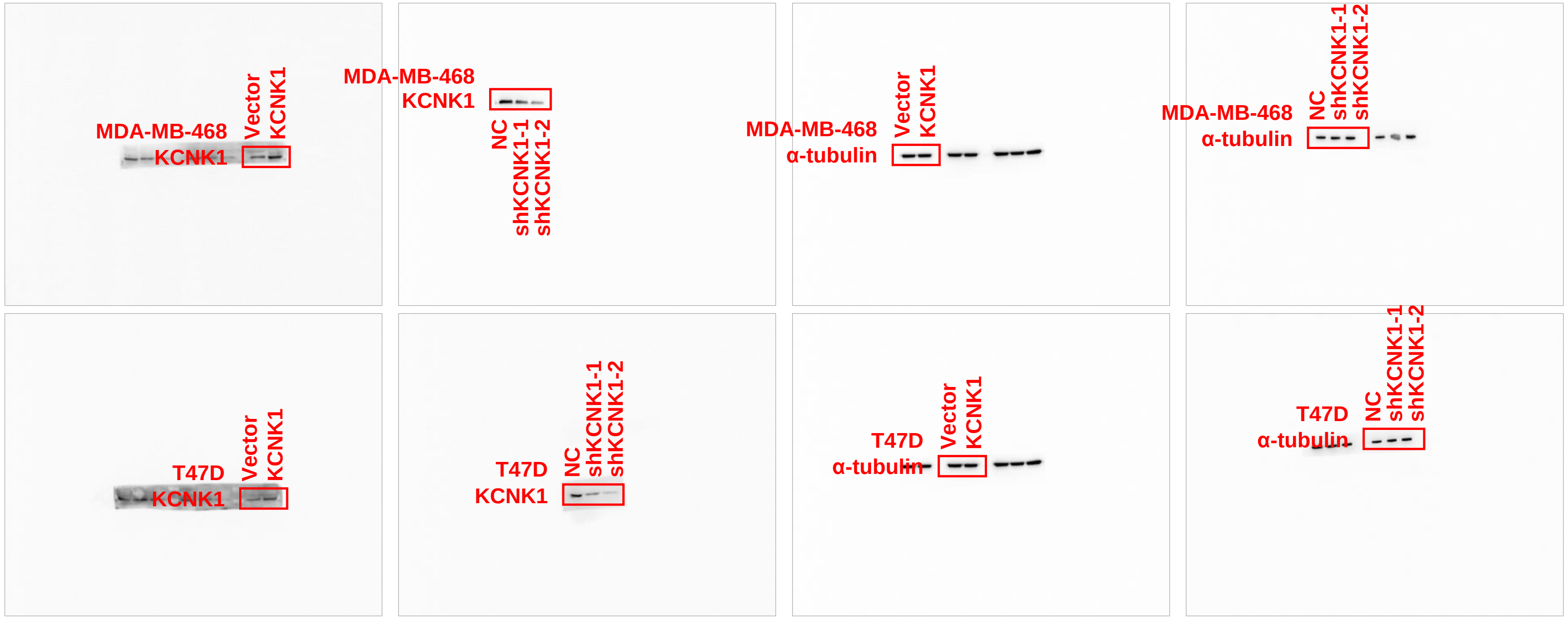

Supplementary Fig. S2b repeat 2

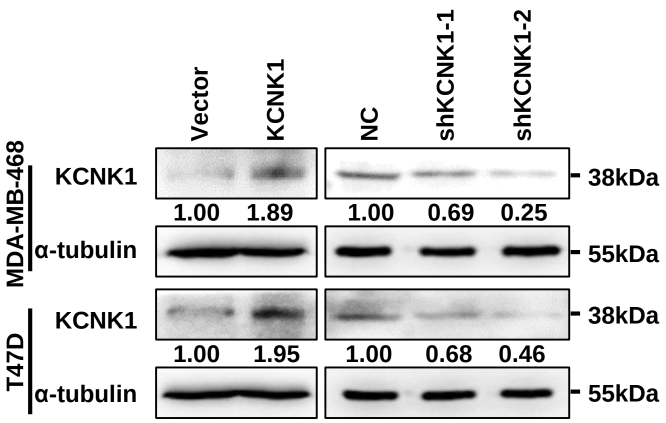

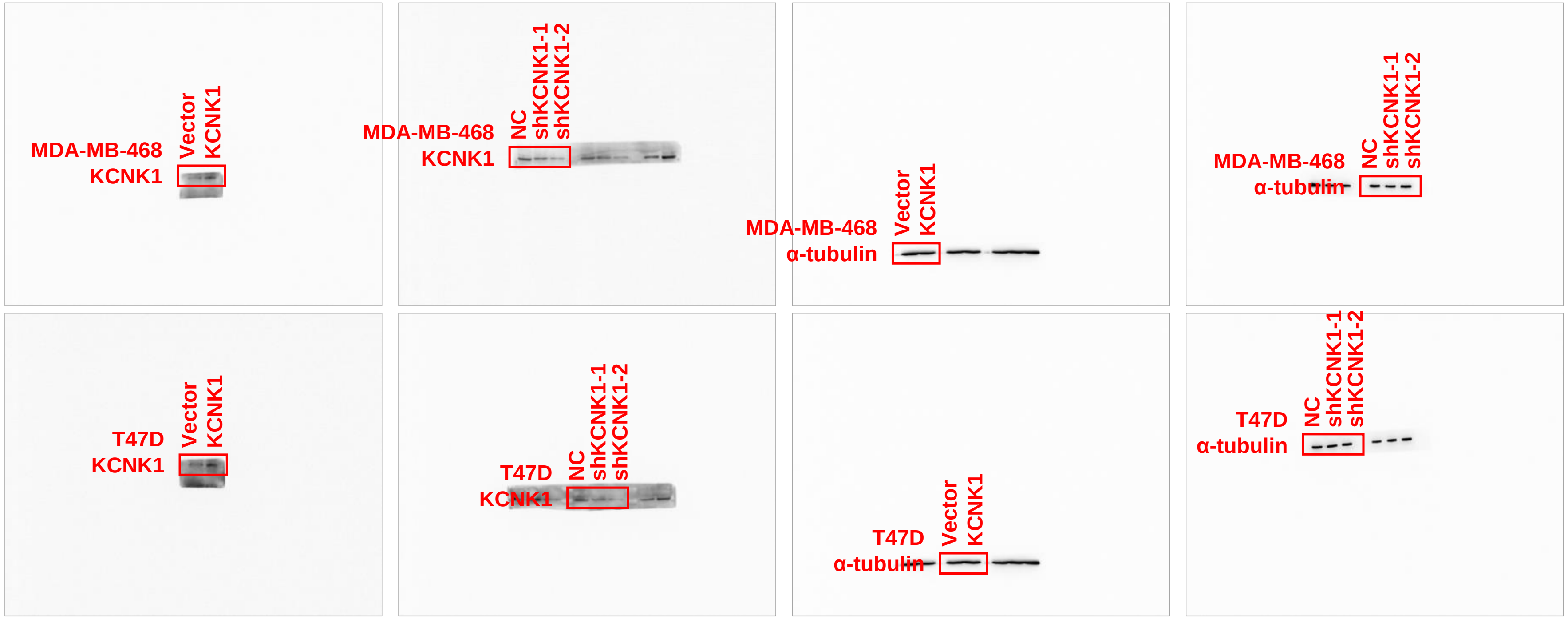

Supplementary Fig. S2b repeat 3

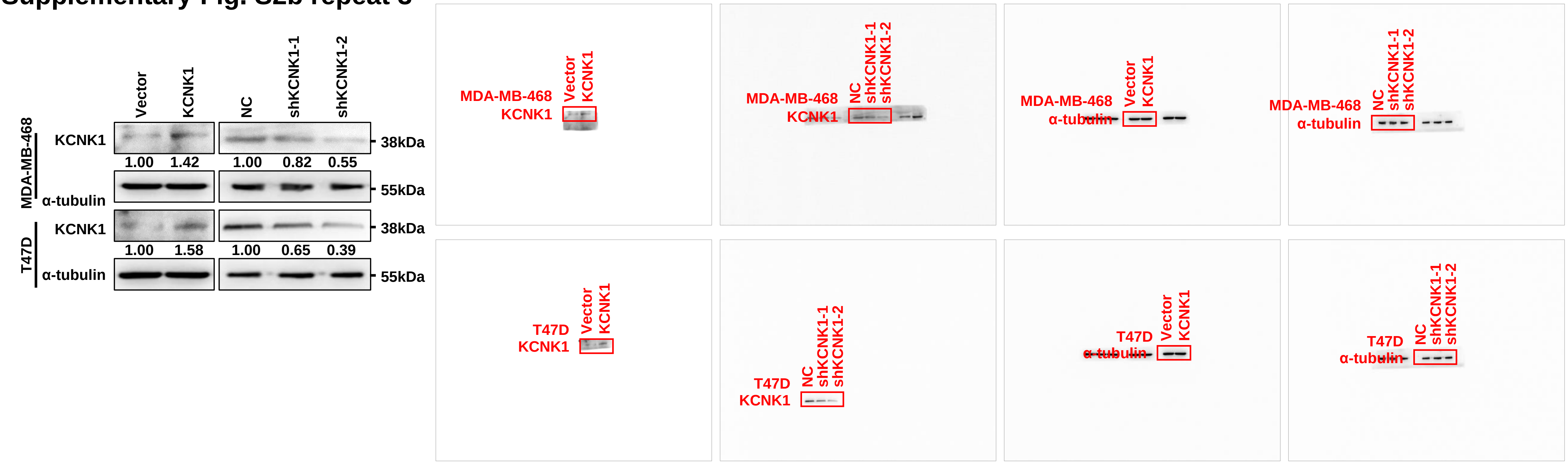

Supplementary Fig. S9a repeat 1

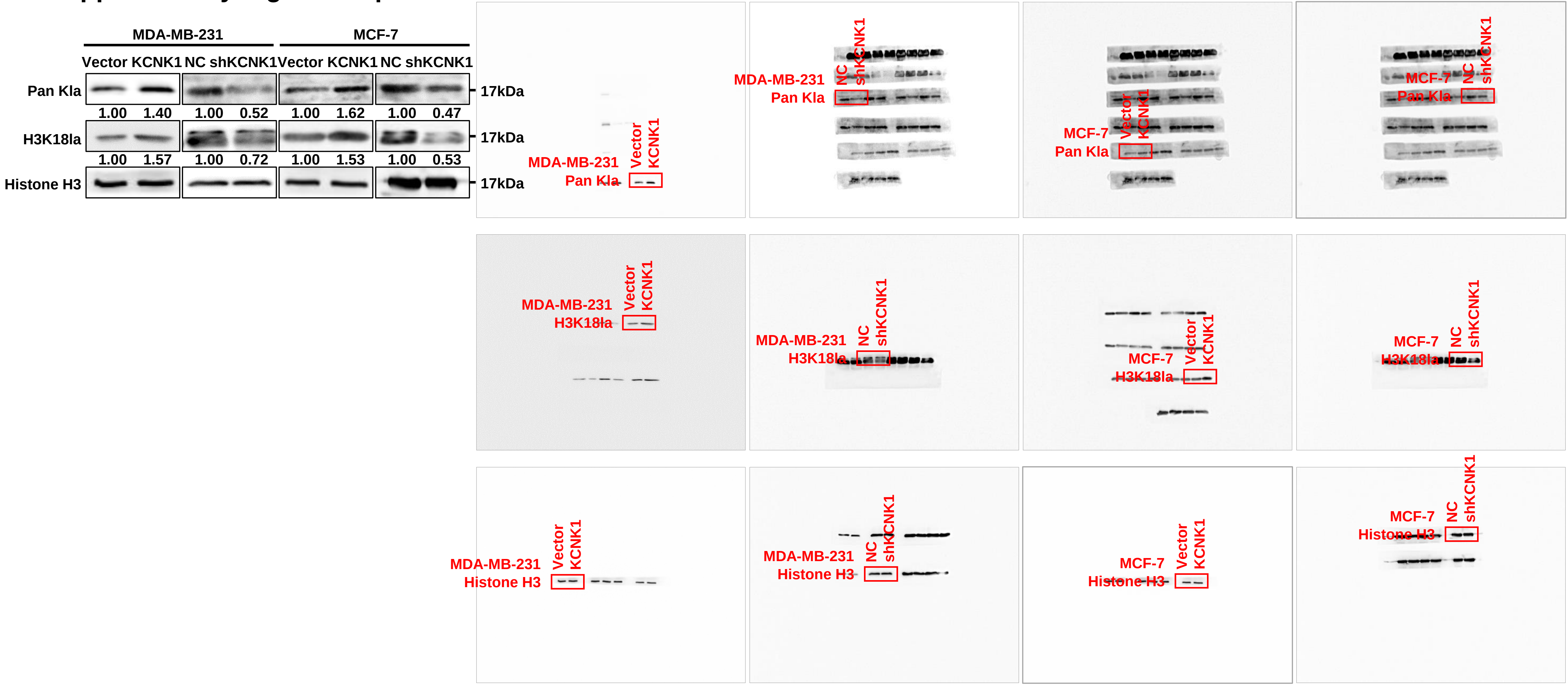

Supplementary Fig. S9a repeat 2

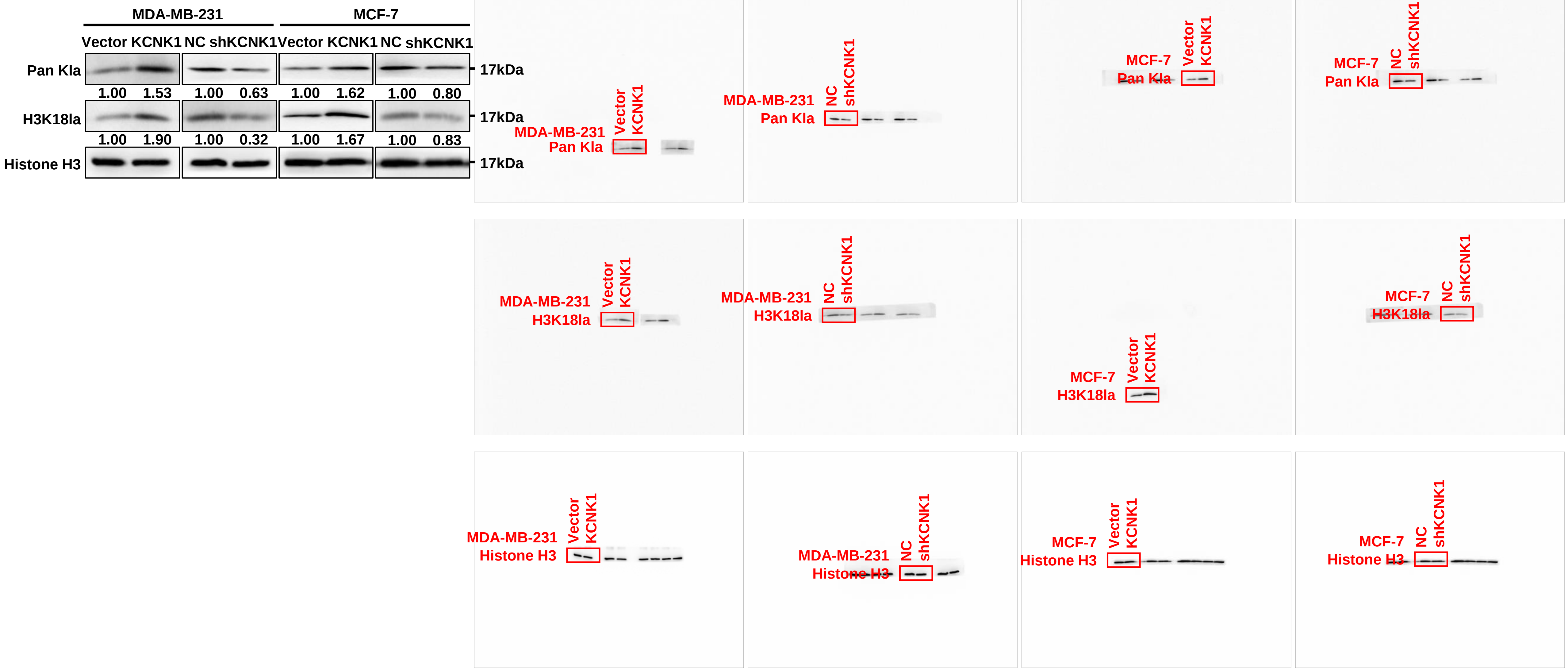

Supplementary Fig. S9a repeat 3

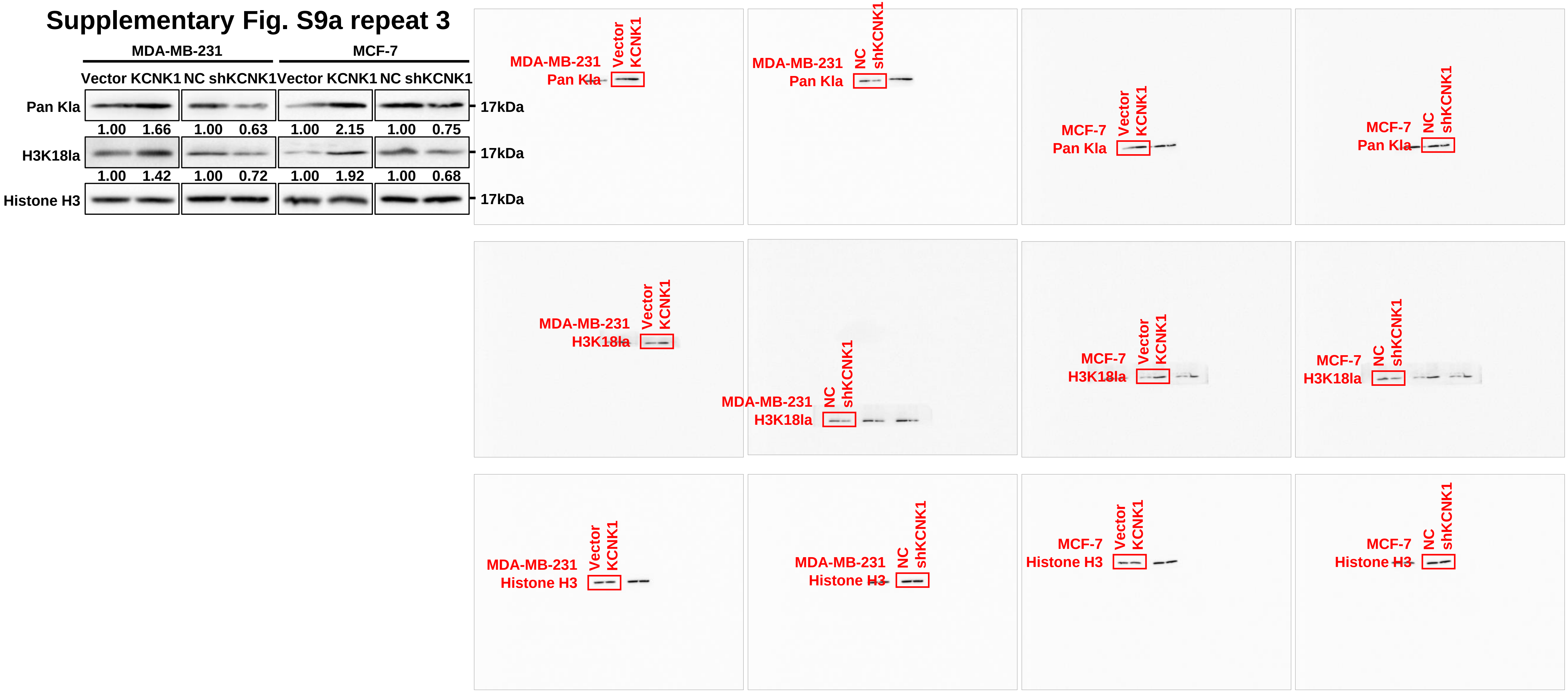

Supplementary Fig. S9e repeat 1

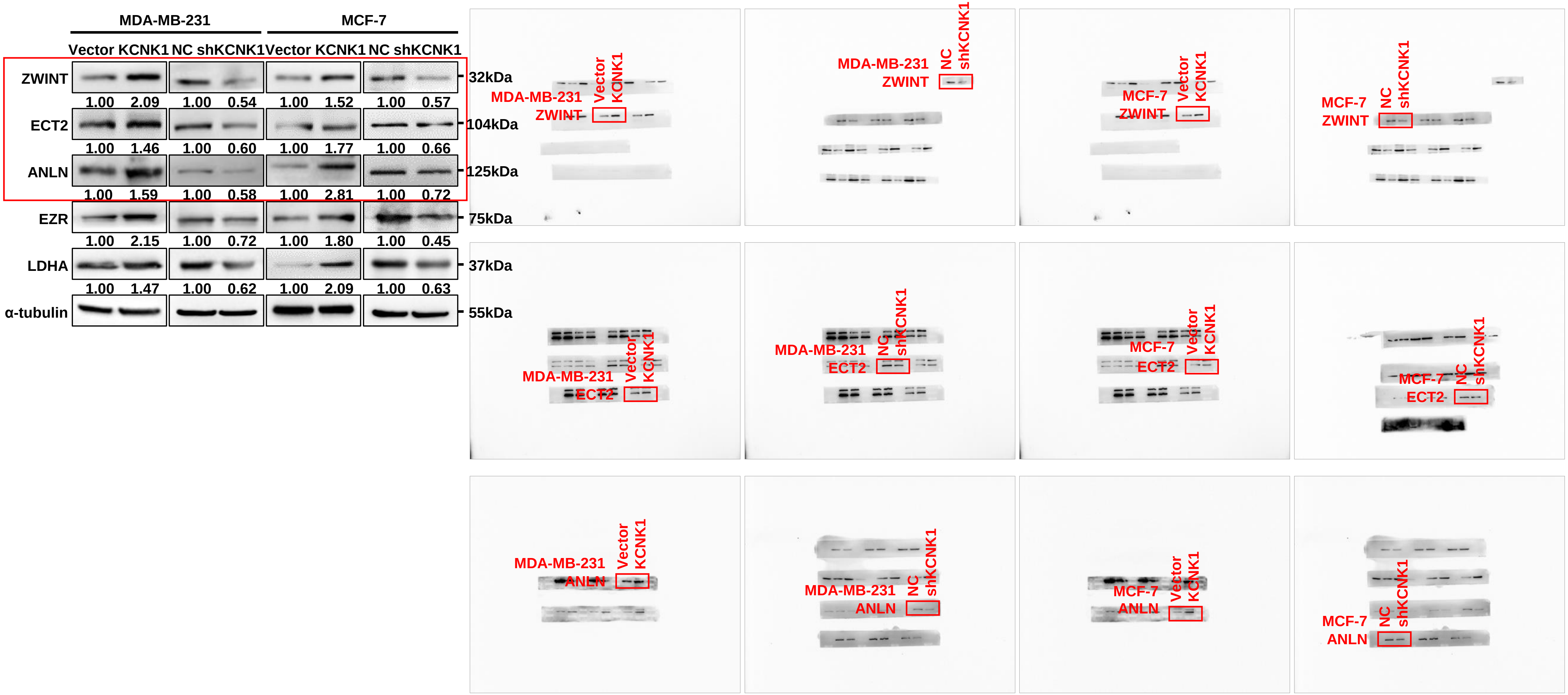

Supplementary Fig. S9e repeat 1

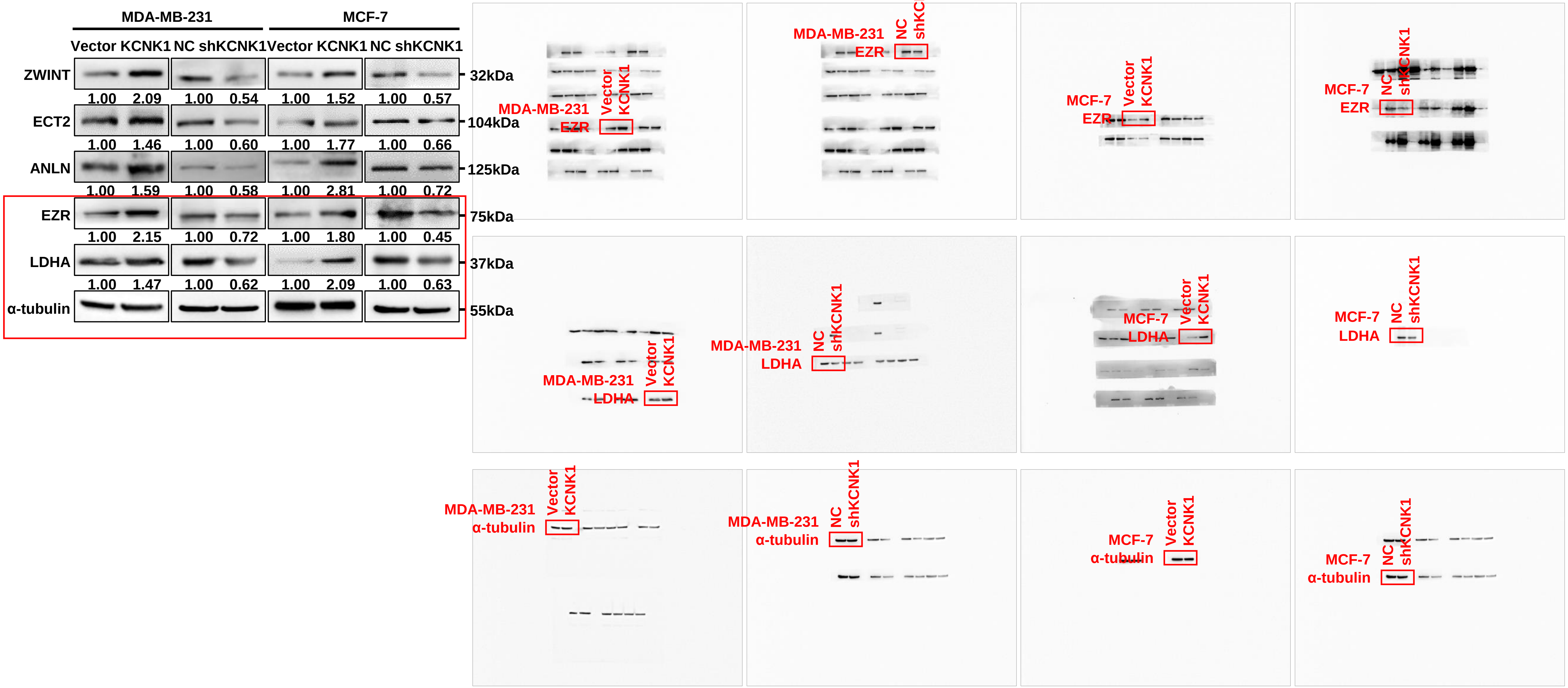

Supplementary Fig. S9e repeat 2

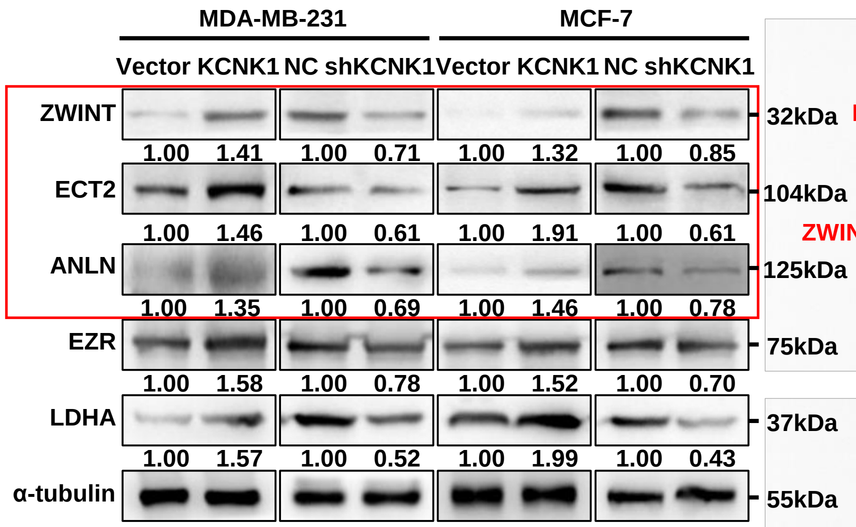

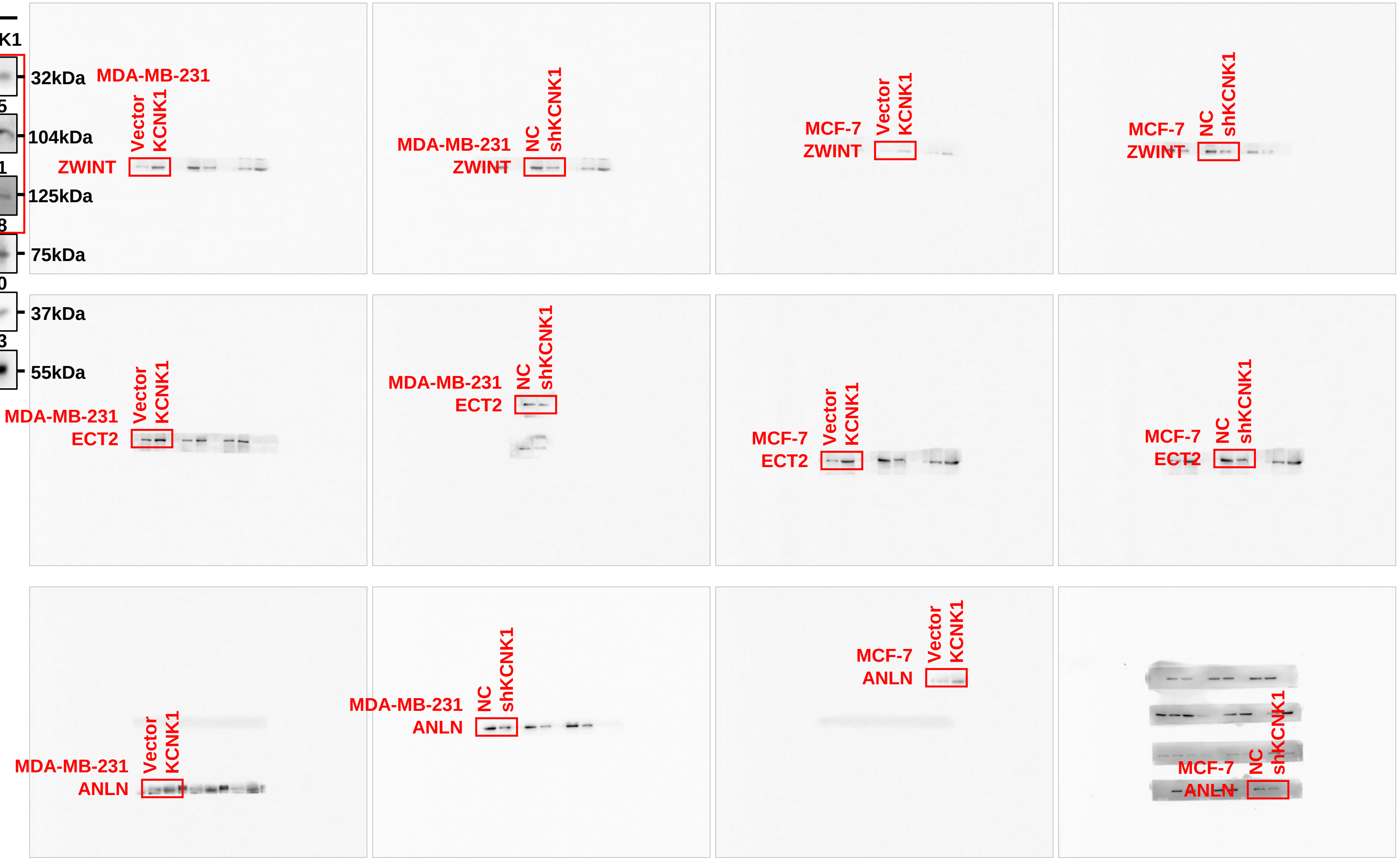

Supplementary Fig. S9e repeat 2

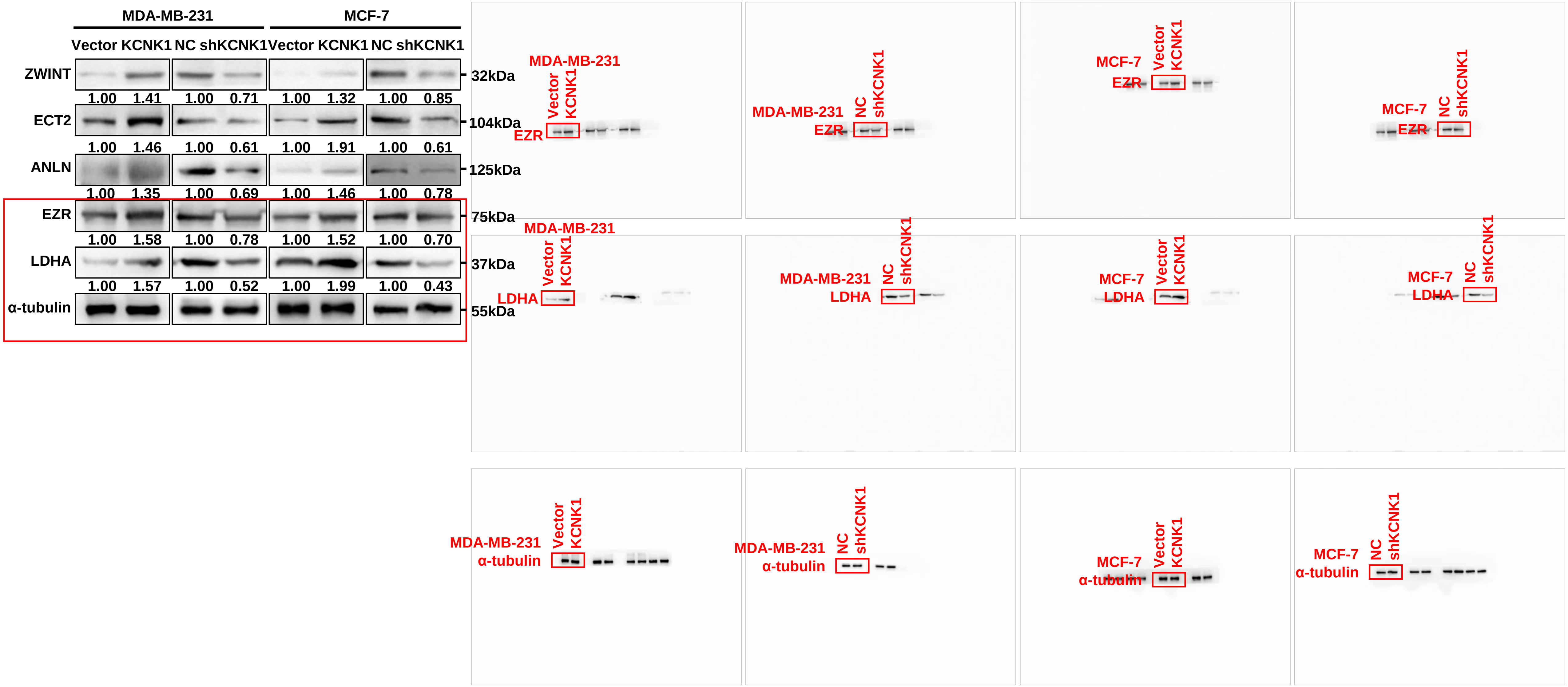

Supplementary Fig. S9e repeat 3

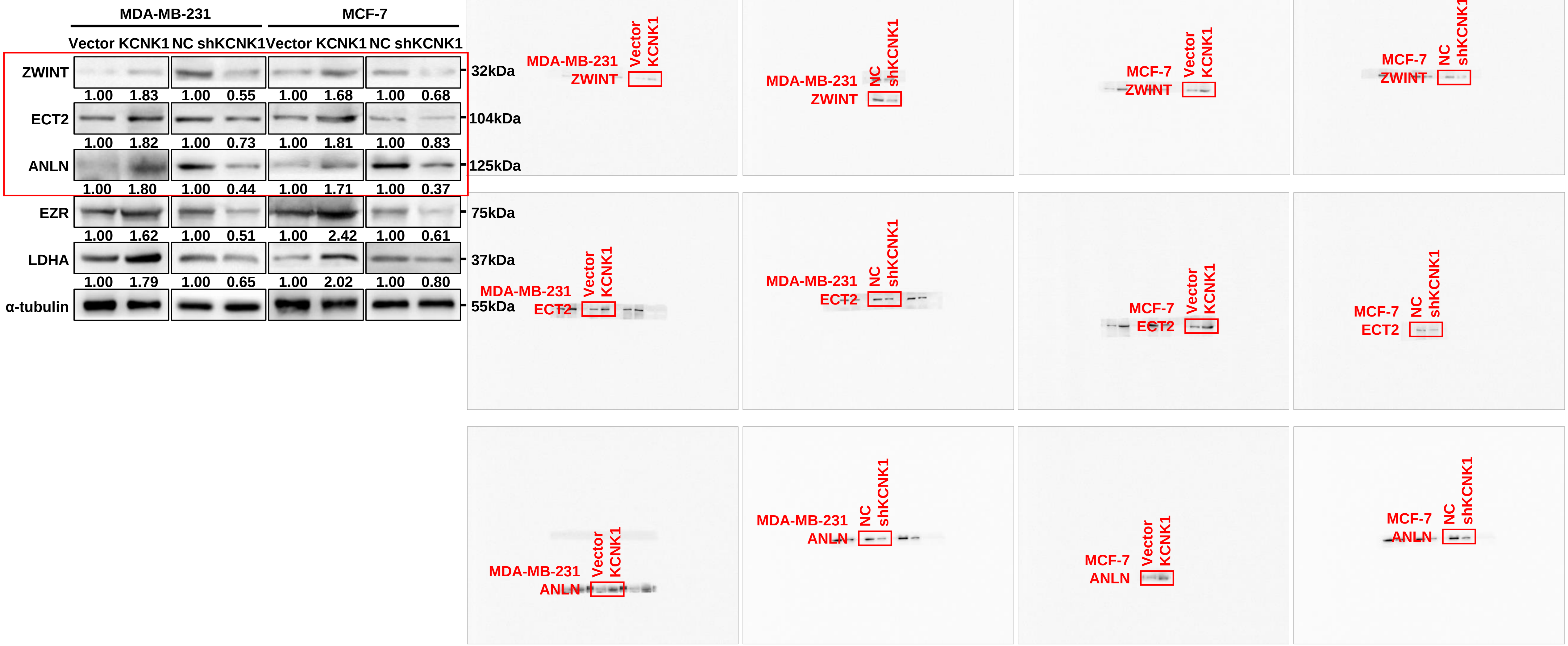

Supplementary Fig. S9e repeat 3

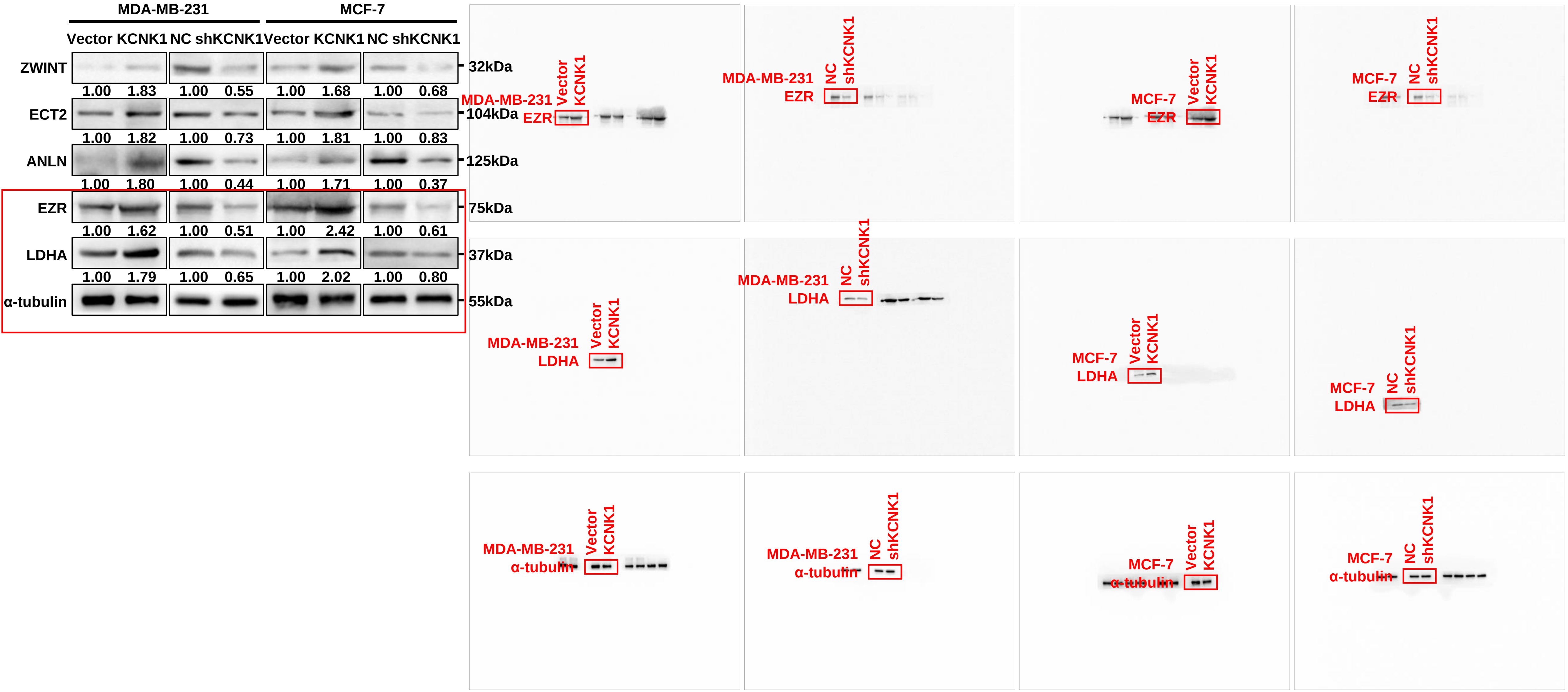

Supplementary Fig. S10a repeat 1

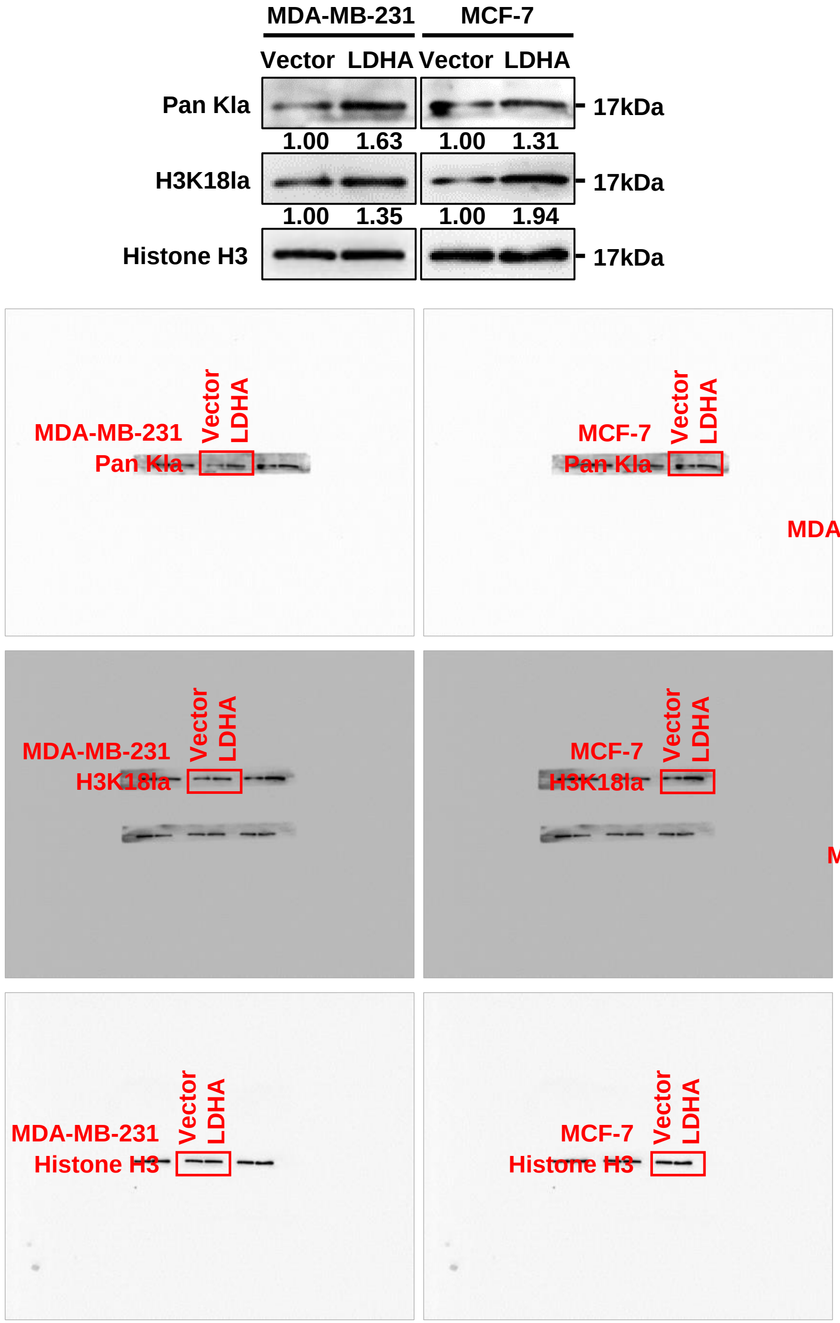

Supplementary Fig. S10a repeat 2

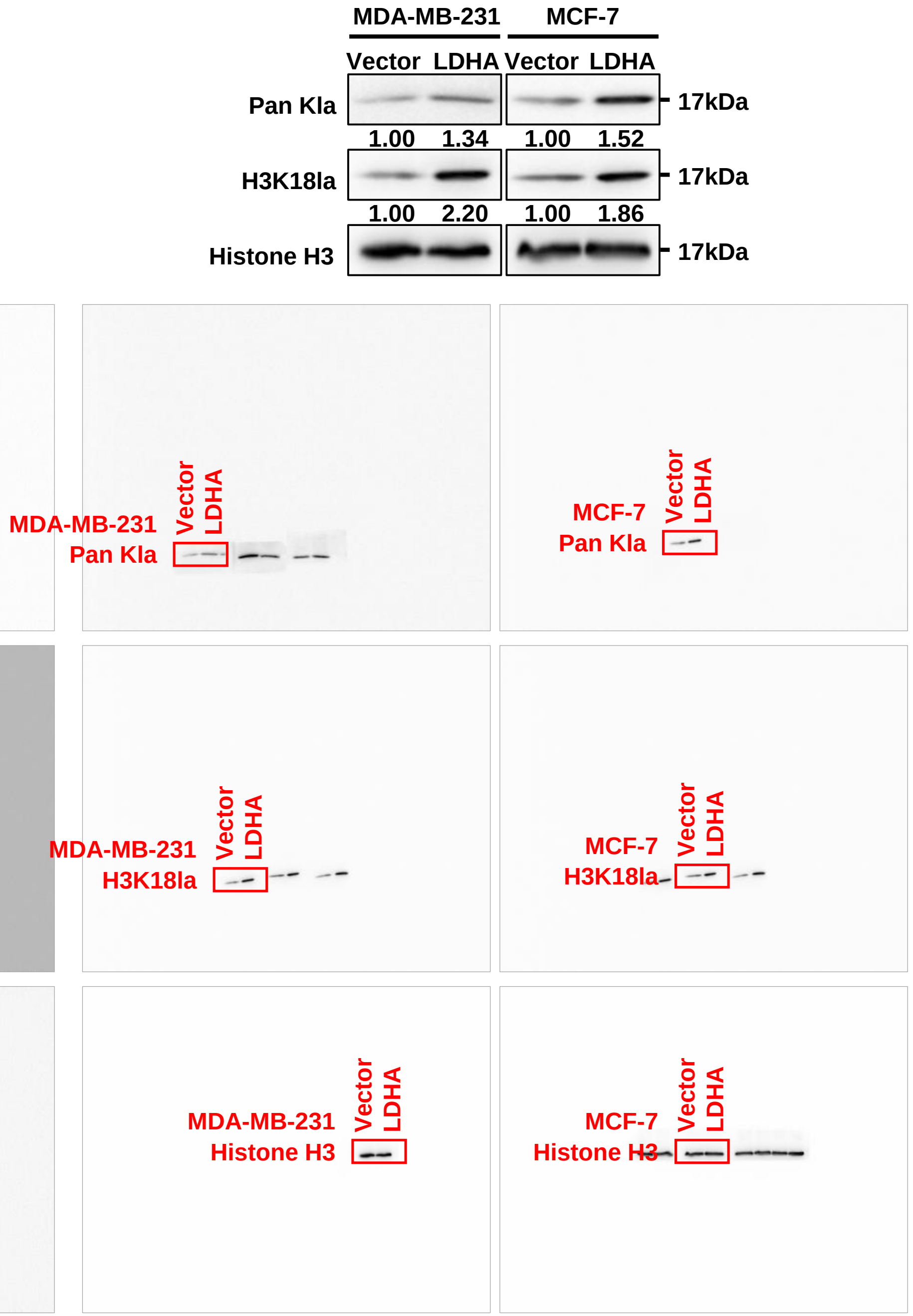

Supplementary Fig. S10a repeat 3

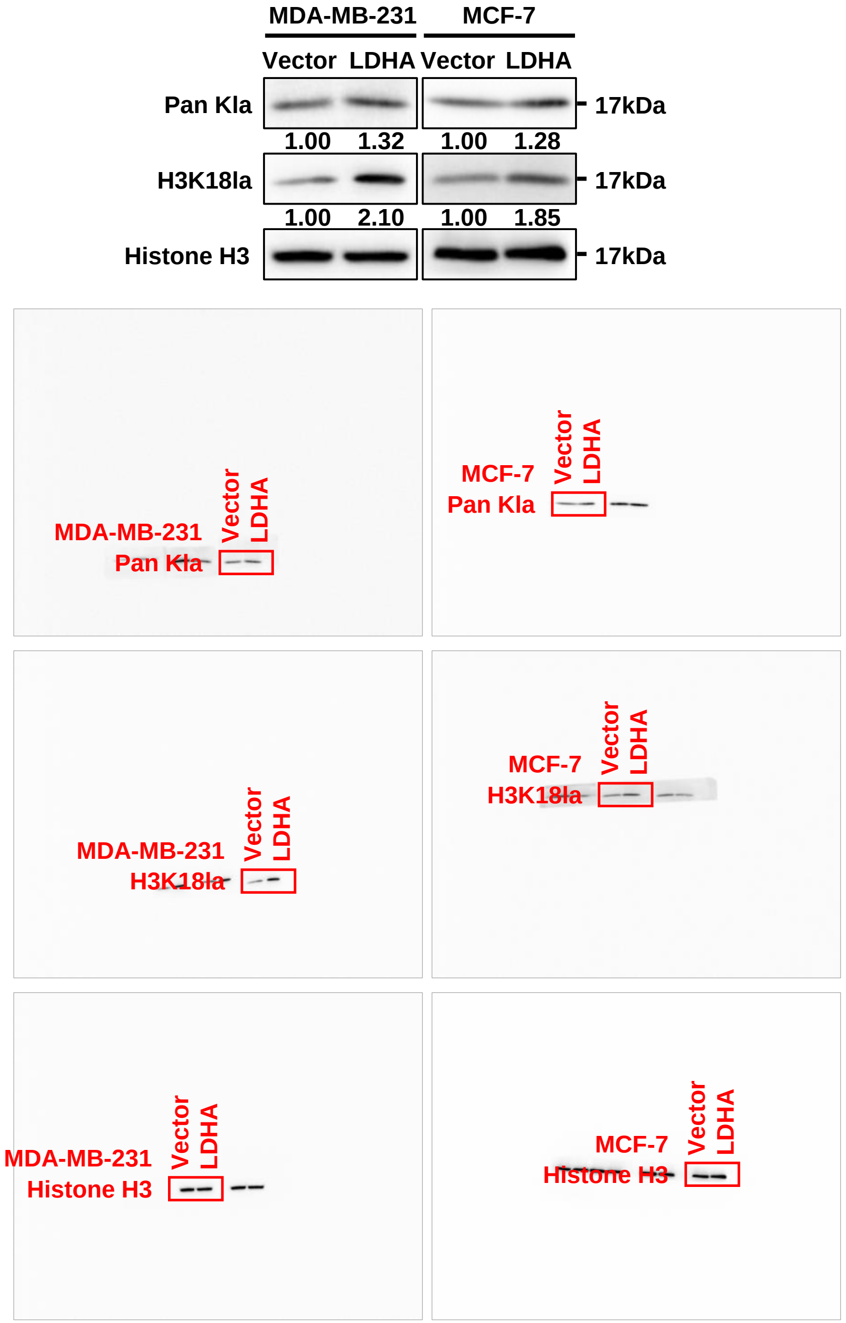

Supplementary Fig. S10e repeat 1

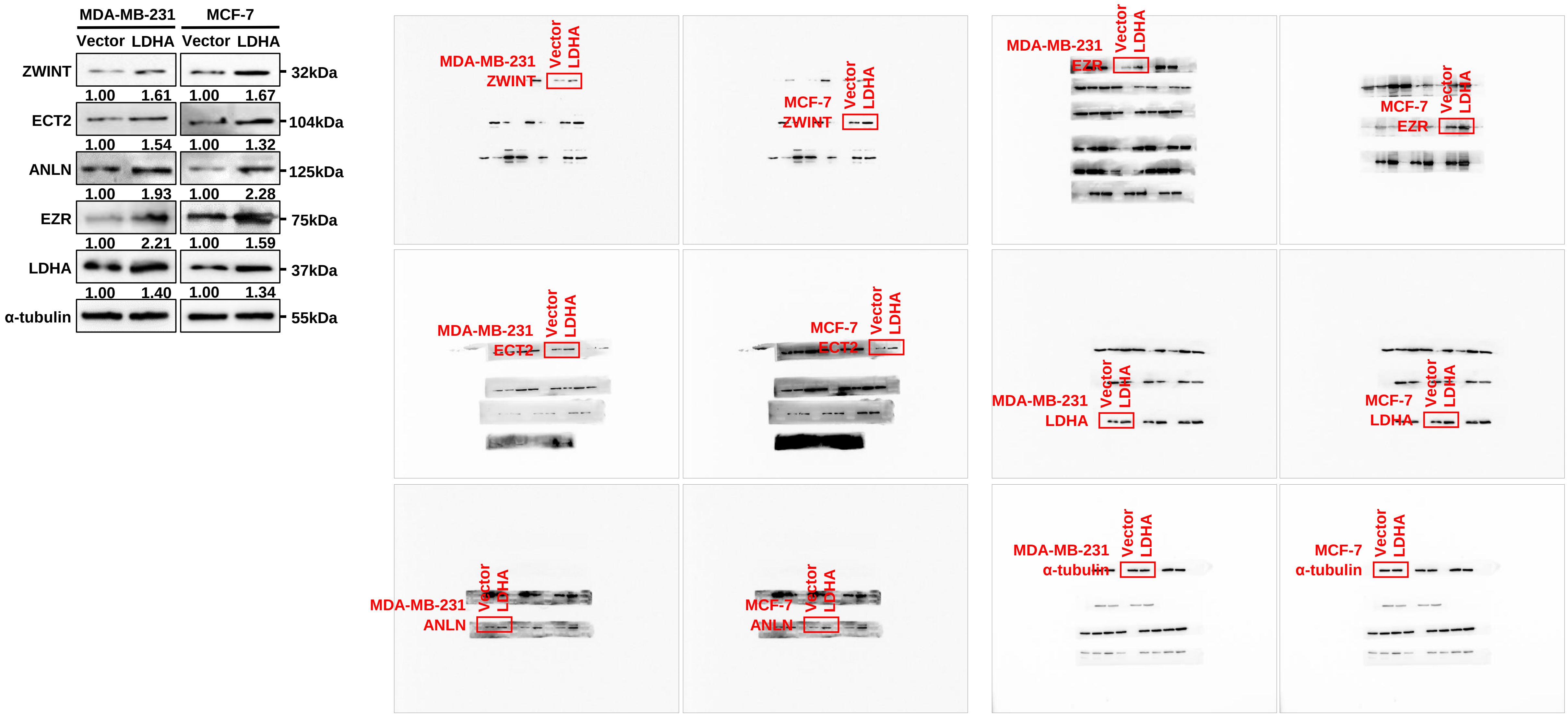

Supplementary Fig. S10e repeat 2

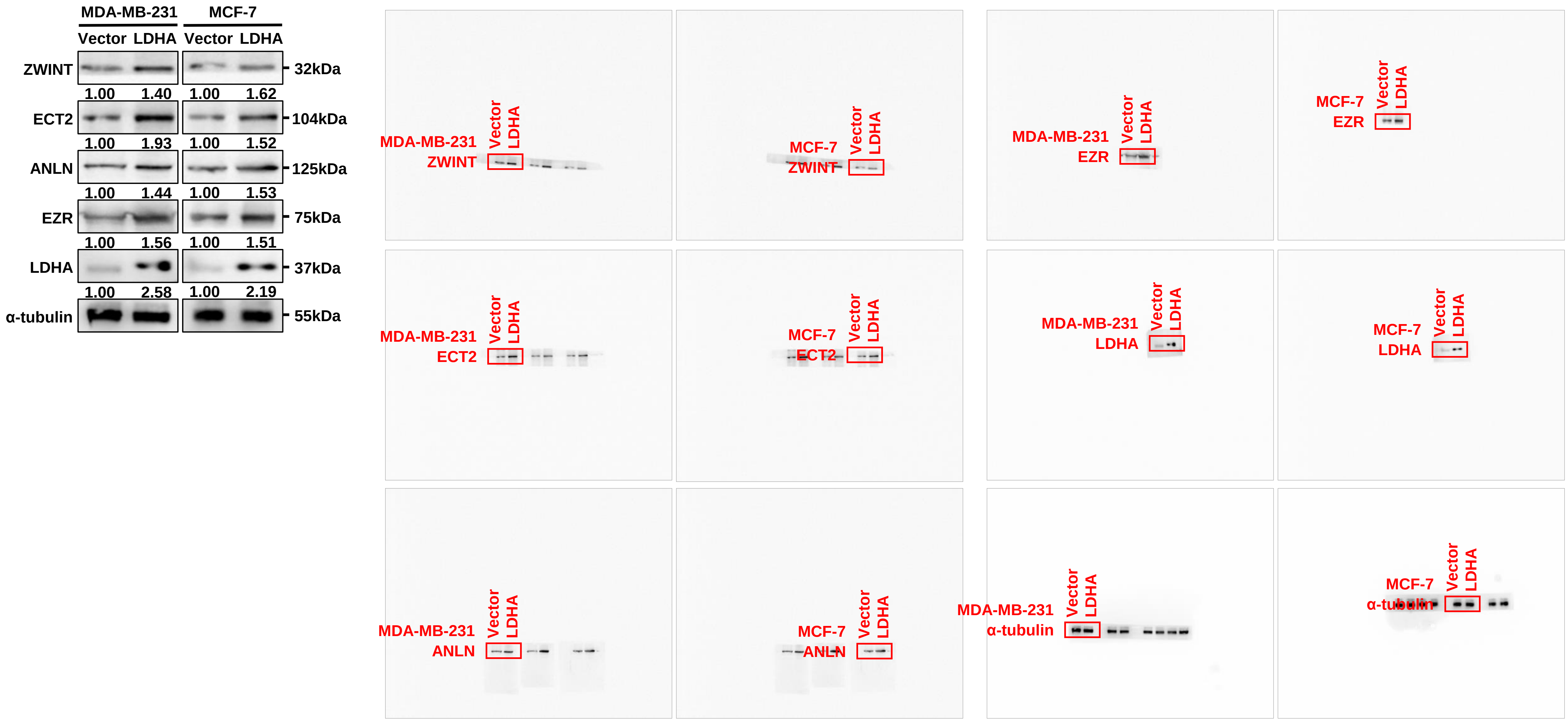

Supplementary Fig. S10e repeat 3

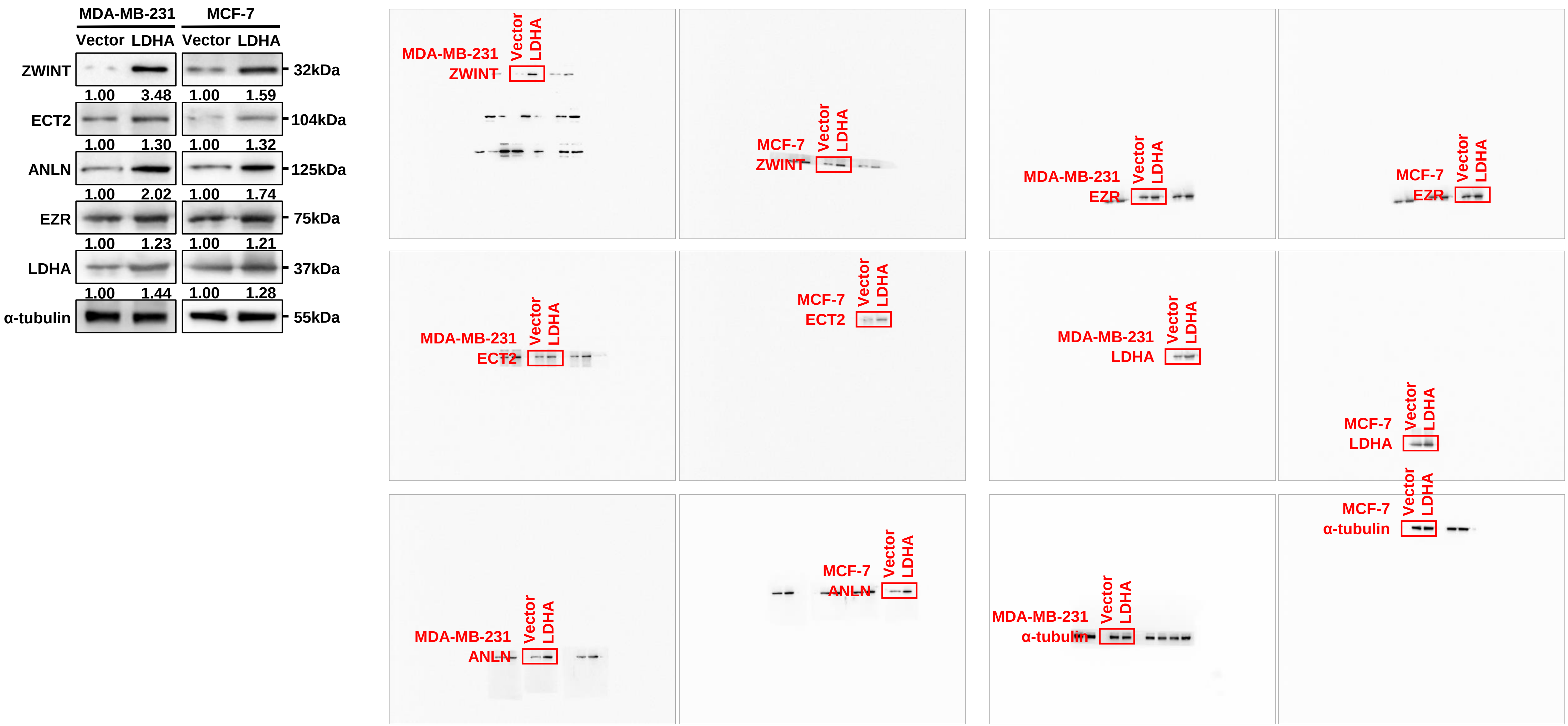

Supplement: S1 Raw Images — (PDF) [file pbio.3002666.s002.pdf]
